# Dynamics in Flexible Pillar[*n*]arenes Probed by Solid-State NMR

**DOI:** 10.1021/acs.jpcc.1c02046

**Published:** 2021-06-15

**Authors:** Ashlea R. Hughes, Ming Liu, Subhradip Paul, Andrew I. Cooper, Frédéric Blanc

**Affiliations:** † Department of Chemistry, University of Liverpool, Crown Street, Liverpool L69 7ZD, United Kingdom; ‡ Materials Innovation Factory, University of Liverpool, 51 Oxford Street, Liverpool, L7 3NY, United Kingdom; § Nottingham DNP MAS NMR Facility, Sir Peter Mansfield Imaging Centre, University of Nottingham, Nottingham NG7 2RD, United Kingdom; ∥ Stephenson Institute for Renewable Energy, University of Liverpool, Crown Street, Liverpool L69 7ZD, United Kingdom

## Abstract

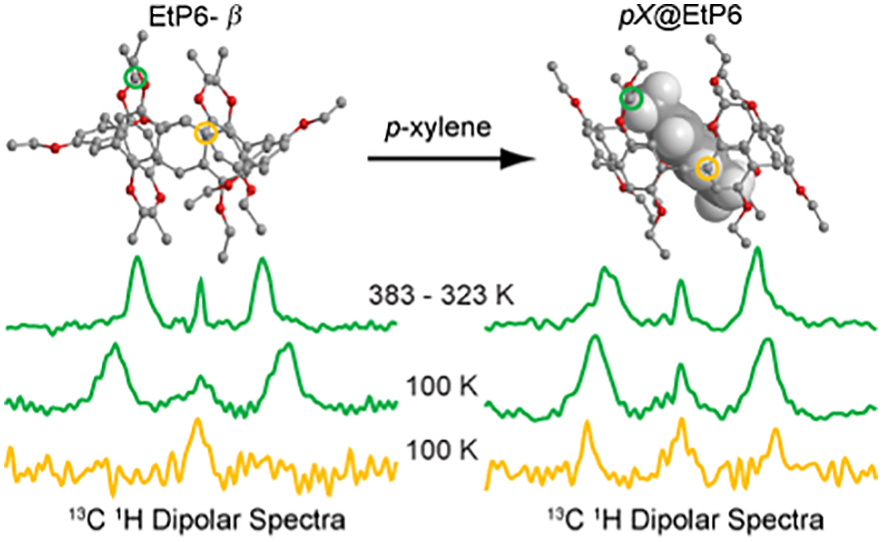

Pillar[*n*]arenes are supramolecular assemblies that can perform a range of technologically important molecular separations which are enabled by their molecular flexibility. Here, we probe dynamical behavior by performing a range of variable-temperature solid-state NMR experiments on microcrystalline perethylated pillar[*n*]arene (*n* = 5, 6) and the corresponding three pillar[6]arene xylene adducts in the 100–350 K range. This was achieved either by measuring site-selective motional averaged ^13^C ^1^H heteronuclear dipolar couplings and subsequently accessing order parameters or by determining ^1^H and ^13^C spin–lattice relaxation times and extracting correlation times based on dipolar and/or chemical shift anisotropy relaxation mechanisms. We demonstrate fast motional regimes at room temperature and highlight a significant difference in dynamics between the core of the pillar[*n*]arenes, the protruding flexible ethoxy groups, and the adsorbed xylene guest. Additionally, unexpected and sizable ^13^C ^1^H heteronuclear dipolar couplings for a quaternary carbon were observed for *p*-xylene adsorbed in pillar[6]arene only, indicating a strong host–guest interaction and establishing the *p*-xylene location inside the host, confirming structural refinements.

## 1 Introduction

**Figure 1:**
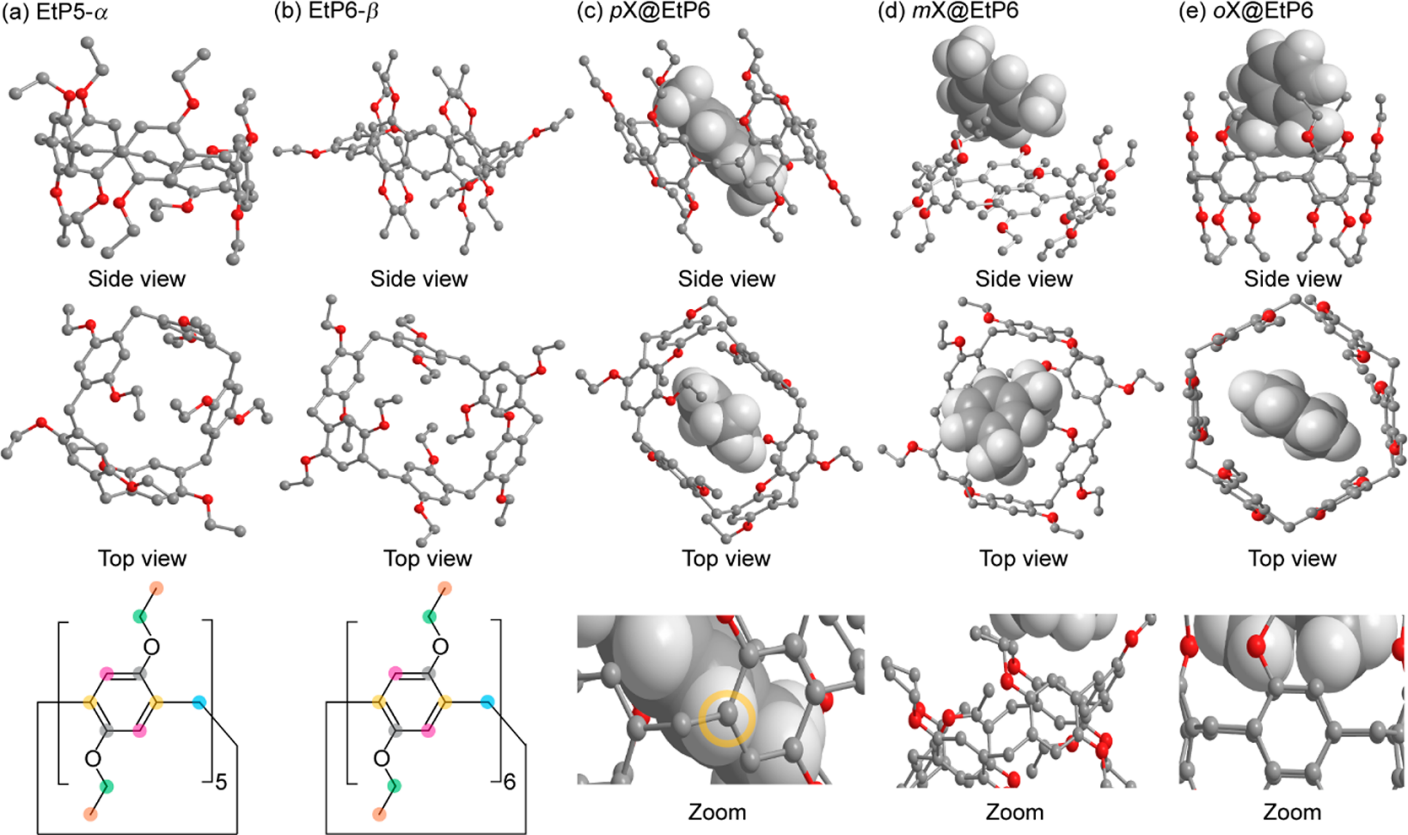
Crystal structures of (a) perethylated pillar[5]arene **EtP5-α** (obtained at 240 K), (b) perethylated pillar[6]arene **EtP6-β** (298 K), (c) *p*-xylene in EtP6 *p***X@EtP6** (240 K), (d) *m*-xylene in EtP6 *m***X@EtP6** (298 K), and (e) *o*-xylene in EtP6 *o***X@EtP6** (100 K).^23^ The side and top views are shown on the first and second rows. The pillar[*n*]arene host and xylene guests are denoted by “ball and stick” and “space filling” models, respectively, with carbons shown in gray, oxygens in red, and protons omitted for clarity in the ball and stick model while shown in white in the space-filling model. The two left panels of the third row provide the chemical structures of both **EtP5-α** and **EtP6-β** using color coding for different carbon environments (*C*H_3_, orange; *C*H_2_, light blue; O*C*H_2_, green; *C*H, pink; CH_2_*C*^IV^, yellow; O*C*^IV^, gray) consistent with those used throughout the NMR spectra assignments. The three right panels of the third row show a magnified view of the through space interaction between the *p*-xylene guest and EtP6 in *p***X@EtP6** (yellow circle) while no interaction is observed for *m***X@EtP6** and *o***X@EtP6** (see text for details).

Host–guest chemistry is an important concept in the field of supramolecular chemistry that is driven by the interactions of molecular assemblies or ions via noncovalent interactions.^1^ These interactions play a vital role in the design of advanced functional materials with exciting physical properties and applications in processes, such as adsorption, catalysis, energy storage, and molecular separations. Consequently, this area has become of increasing importance over the past few decades,^2−5^ and a wide range of supramolecular assemblies that adapt to guests^6^ has been discovered thanks to a large variety of tunable structural motifs and properties (e.g., solubility, functionality, and molecular flexibility). Among those, pillar[*n*]arenes (*n* = 5–15) have emerged as a novel class of easily functionalized supramolecular macrocycles^7−10^ whose structure consists of substituted phenolic moieties repeated *n*-times and connected in the *para* position by methylene linkages (Figure 1). For most values of *n* (except *n* = 7), the resulting architecture is a symmetrical cylindrical structure (side view, Figure 1) leading to a symmetrical polygon (top view) that yields a single pentagonal and hexagonal cavity for *n* = 5 and 6, respectively, and two pentagonal and/or hexagonal cavities for *n* > 7. The cavity plays an important role in hosting appropriately sized guest molecules for capture/molecular separation^11−16^ and controlled delivery systems.^17,18^

Pillar[*n*]arenes (*n* = 5,6) have found the greatest interest, mostly due to their relatively small cavity sizes that enable them to host small molecules,^10^ combined with substituted alkyl and branched chains that strongly affect the host–guest properties.^19−22^ Perethylated pillar[*n*]arene (*n* = 5, EtP5; *n* = 6, EtP6) are examples of these substituted pillar[*n*]arenes that contain ethoxy groups (Figure 1a,b) with EtP6 existing as two polymorphs, a metastable **EtP6-α** phase and a crystalline **EtP6-β** phase.^23^ Due to its large conformational flexibility, EtP6 has been found to adsorb a number of guest molecules,^14,23^ and we have recently shown that **EtP6-β** adapts during adsorption of an *o*-xylene (*o***X**)/*m*-xylene (*m***X**)/*p*-xylene (*p***X**) isomer mixture to efficiently capture *p***X** with a high selectivity of 90% to form *p***X@EtP6** (Figure 1c),^23^ while *m***X@EtP6** (Figure 1d) and *o***X@EtP6** (Figure 1e) are obtained by adsorption of the respective xylene isomer into **EtP6-β**. This is a step forward for the energy efficient separation of the xylene isomers, which are widely used as chemical feedstocks.^23,24^

One important criterion for these supramolecular structures is their adaptivity and flexibility that dictates the adsorption of guest molecules in the cavity space and which are not yet understood. This adaptive behavior is not trivial to probe, especially in the solid state, given the requirements to access experimentally measurable observables that depend on dynamics and which need to be determined at the resolution of each local chemical environment. Solid-state nuclear magnetic resonance (NMR), often in conjunction with computational methods such as crystal structure prediction (CSP) and diffraction-based approaches, provides detailed, element-specific, and structural information on the atomic scale and plays an important role in supramolecular assemblies.^25−31^ For example, we took advantage of the very high spectral resolution of the ^13^C NMR spectra of **EtP5-α**, **EtP6-α** and **EtP6-β** to support the conformational energy landscape exploration and identify the number of different carbons in the asymmetric unit cell.^23^ NMR is also well suited to probe site selective molecular flexibility given its sensitivity to molecular motion over wide time scales from fast processes (subnanoseconds via relaxation measurements) to slower dynamics (milliseconds from line shape analysis), offering a unique access to the qualitative and quantitative description of motion.^28,32^

Recent ^2^H NMR work has focused on the molecular dynamics on *n*-hexane-*d*_14_ in pillar[5]arene that showed that the molecular diversity gave rise to different patterns of guest uptake and release.^33^ Liquid state NMR has previously been used to investigate conformational properties^34−37^ and assess *p*-phenylene unit rotation in pillar[*n*]arenes, but little is known regarding the adaptive behavior of these materials in the solid state.

Here, we determine the dynamics of both guest-free **EtP5-α** and **EtP6-β** and the three xylene-adsorbed perethylated pillar[6]arenes over a range of time scale by probing site selective ^13^C ^1^H heteronuclear dipolar couplings and accessing ^1^H and ^13^C correlation times as a function of temperature (383–100 K). We find that the flexibility of the protruding O*C*H_2_ groups in the guest-free pillar[*n*]arenes is reduced when there are fewer phenolic moieties, or at temperatures below 298 K, as well as by adsorption of xylene isomers; by contrast, other carbon groups have largely similar dynamics over the temperature range studied. We identify intermolecular ^13^C ^1^H dipolar couplings at low temperatures in *p***X@EtP6** which are absent on both *o***X@EtP6** and *m***X@EtP6**, which provides evidence for the location of xylenes in the EtP6 architecture and highlights the host–guest interactions. Finally, we exploit variable temperature spin–lattice relaxation measurements to access dynamics in the MHz regime, which confirm the flexibility of the extruding ethoxy groups of these pillar[*n*]arenes as opposed to the carbon atoms located in the ring core.

## 2 Experimental Section

### 2.1 Materials Synthesis

Guest-free **EtP5-α**(38) and **EtP6-β**(38) and the three xylene-adsorbed perethylated pillar[6]arenes^23^ were synthesized using established literature procedures (Scheme S1).^38^ Prior to adsorption, powder X-ray diffraction (PXRD, Figure S1) and NMR measurements, **EtP5-α** and **EtP6-β** were dried and heated under vacuum at a pressure of 10^–3^ mbar to 433 K for 2 h to ensure no solvation and that the correct phases were obtained. *p***X@EtP6** and *m***X@EtP6** were synthesized using the xylene vapor adsorption method, whereas *o***X@EtP6** was prepared via solvent evaporation with adsorption time longer than 12 h to ensure the presence of one molecule of xylene per EtP6. Differential scanning calorimetry (DSC) data on **EtP6-β** identifies a phase change at 339 K (Figure S2). Thermogravimetric analysis (TGA) data on *p***X@EtP6**,^23^*m***X@EtP6** (Figure S3), and *o***X@EtP6** (Figure S4), combined with time-dependent sorption data from ^1^H solution-state NMR spectroscopy spectra of dissolved crystals and single-crystal X-ray diffraction data on *p***X@EtP6**, *m***X@EtP6**, and *o***X@EtP6**,^23^ establish the stoichiometry of one xylene adsorbed per EtP6.

### 2.2 NMR Experiments

The ^1^H and ^13^C solid-state NMR experiments at an external magnetic field *B*_0_ = 9.4 T were performed on a Bruker Avance III HD NMR spectrometer equipped with a 4 mm HXY triple-resonance magic angle spinning (MAS) probe in double-resonance mode tuned to Larmor frequencies of ν_0_(^1^H) = 400.13 MHz and ν_0_(^13^C) = 100.62 MHz. The *B*_0_ = 14.1 T NMR experiments were performed on a 14.1 T Avance III DNP NMR spectrometer equipped with a low temperature 3.2 mm HXY triple-resonance MAS probe^39^ in double-resonance mode tuned to ν_0_(^1^H) = 600.25 MHz and ν_0_(^13^C) = 150.93 MHz. All experiments were obtained under MAS with the sample spinning at ν_r_ = 12.5 kHz, unless otherwise specified. ^1^H pulses and SPINAL-64 heteronuclear decoupling^40^ during ^13^C acquisition were performed at a radio frequency (rf) field amplitude of 83 kHz for all samples except the room temperature cross-polarization (CP) experiments on the guest-free samples where it was performed at 96 kHz. ^13^C pulses were performed at a rf field of 60 and 70 kHz at 9.4 and 14.1 T, respectively. For all data obtained at 14.1 T, a presaturation block consisting of 100 ^1^H pulses separated by 1 ms was used (all pulse sequences are described in Figure S5 and Section S5 of the SI). For variable-temperature experiments, zirconia drive caps were used at 9.4 T and Vespel caps at 14.1 T. Additional ^1^H one pulse quantitative spectra were obtained at *B*_0_ = 20 T on a Bruker Avance III NMR spectrometer and under MAS at ν_r_ = 60 kHz using a 1.3 mm HXY triple-resonance MAS probe in double resonance mode tuned to a Larmor freuqency of ν_0_(^1^H) = 850.13 MHz; spectra were acquired with a rf field amplitude of 150 kHz.

In the variable-temperature CP experiments, the CP steps were performed with a ^13^C rf field of 41 kHz (at 9.4 T) and 70 kHz (at 14.1 T) while the ^1^H rf field amplitude was ramped to obtain maximum signal at approximately 65 kHz (at 9.4 T) and between 70–96 kHz (at 14.1 T), dependent on samples and temperatures. An optimized contact time of 1.5–3.0 ms was used. Typically, ^13^C CP experiments were accumulated with 2048 scans (at 9.4 T) and 32–2048 scans (at 14.1 T), and used recycle delays of 1.3 × ^1^H *T*_1_(41) (with *T*_1_ being the spin–lattice relaxation times measured as given below) that corresponds to the maximum signal-to-noise per unit time. Note that although ^13^C CP MAS experiments are not quantitative, only ^13^C integration within a chemically distinct carbon environment is given as its similar nature allows comparison of the number of carbons to be estimated.

Variable temperature ^1^H and ^13^C spin–lattice relaxation times *T*_1_’s were obtained with the saturation recovery and *T*_1_ Torchia^42^ pulse programs, respectively. In the saturation recovery experiment, the magnetization is saturated by a presaturation block consisting of 100 ^1^H pulses separated by 10 ms at 9.4 T or 1 ms at 14.1 T, followed by magnetization buildup during a variable τ delay and NMR detection. In the *T*_1_ Torchia sequence,^42^ an initial ^13^C CP step creates ^13^C magnetization which then decays during a variable delay τ and ^13^C detection is achieved using a two-step phase cycle to account for the direct (unenhanced) ^13^C Boltzmann value rather than CP enhanced values. The data obtained via integrated intensities were fitted to stretch exponential functions of the form of 1 – exp[−(τ/*T*_1_)^α^] and exp[−((τ/*T*_1_)^β^] for the ^1^H and ^13^C *T*_1_ data, respectively, where α (between 0.75 and 0.96) and β (between 0.60 and 0.88) are the respective stretch exponential factors. Errors associated from the *T*_1_ values are quoted to a 95% confidence level and are smaller than the symbol sizes in all figures.

Variable-temperature 2D proton detected local field (PDLF) spectra correlating ^13^C NMR spectra in the direct frequency dimension ω_2_ with ^13^C ^1^H dipolar coupling spectra in the indirect ω_1_ dimension were obtained using the windowed^43^ sequence (wPDLF)^44^ and R-type recoupling blocks.^45^ The sequence starts with the reintroduction of the heteronuclear ^13^C ^1^H dipolar coupling under MAS during the rotor synchronized evolution period *t*_1_ using the symmetry-based R18_2_^5^^1^H recoupling block^46^ which was optimized for maximum signal around the ^1^H rf field amplitude of approximately 9 × ν_r_ (112.5 kHz). R18_2_^5^ also removes the homonuclear ^1^H ^1^H dipolar coupling^46^ and the 180° phase shift in the recoupling block refocuses the (small) ^1^H chemical shift anisotropy (CSA), while the synchronized 180° ^13^C pulse applied in the middle of *t*_1_ prevents the same refocusing from occurring for the heteronuclear ^13^C ^1^H dipolar coupling and refocuses the ^13^C chemical shift. The ^13^C CSA is averaged out over two rotor periods. The ^13^C magnetization is therefore only modulated by the ^13^C ^1^H dipolar coupling in *t*_1_ that yields a ^13^C ^1^H dipolar coupling spectra in ω_1_. Polarization transfer to ^13^C is subsequently achieved using the rotor synchronized PRinciples of Echo Shifting using a Train of Observations (PRESTO)^47^ pulse sequence optimized for maximum signal for the protonated resonances to a length of  × τ_r_ (142 μs), where τ_r_ is the rotor period (80 μs), and by varying the recoupling length of the R18_1_^7^^1^H recoupling block (which is also optimized to a similar ^1^H rf field of approximately 9 x ν_r_ (112.5 kHz)). PRESTO is preferred to CP for polarization transfer as ^1^H spin diffusion in the latter results in an increase of the signal intensity for the zero frequency signal.^44^ Following Fourier transformation in the F1 dimension, an effective dipolar coupling constant κ_R_*d*_CH_ (with κ_R_ the scaling factor of the wPDLF sequence and *d*_CH_ the dipolar coupling constant, see SI Section S6 including Table S1 and Figure S6 for the experimental determination of κ_R_) is obtained in the ω_1_ frequency dimension.^44,48,49^ The (scaled) ^13^C ^1^H dipolar coupling spectra are then extracted at each ^13^C isotropic chemical resonances (δ_iso_) and the dipolar coupling values are obtained from the distance between the outer singularities to yield site-specific motional averaged dipolar coupling ⟨*d*_CH_⟩ values. Note that the small variation of these values obtained from each carbon resonance for a particular carbon subgroup (an example of which is given in Figure S7 for the *C*H_3_ resonance of **EtP6-β**) has been used to provide estimated errors and we have chosen to give a single averaged ⟨*d*_CH_⟩ value for each carbon subgroup.

Static dipolar coupling constants *d*_CH_ were calculated from eq 1 and carbon proton bond lengths. These were obtained from computed CSP^23^ data for the **EtP5-α** and **EtP6-β** conformers or experimental low temperature high resolution powder neutron diffraction data from *o*-xylene^50^ and *m*-, *p*-xylenes^51^ crystal structures for the xylenes.

Temperature calibrations were preformed prior to NMR data acquisition using either the ^207^Pb chemical shift thermometer of Pb(NO_3_)_2_^52,53^ or the ^79^Br *T_1_*s^54^ of KBr (extracted from polarization build-up curves using the saturation recovery pulse sequence) according to procedures outlined in the literature. All temperatures reported are actual sample temperatures and have an estimated accuracy of ±10 K. NMR data were processed with TopSpin and MATLAB R2019a.^55^^1^H and ^13^C spectra were referenced to H_2_O at 4.8 ppm and the CH of adamantane at 29.45 ppm,^56^ respectively, both relative to TMS primary reference at 0 ppm. Small deviations in the observed isotropic chemical shifts (±0.7 ppm in ^13^C CP MAS NMR spectra) is likely attributed to small changes in shim coil temperatures during variable temperature experiments.

## 3 Results and Discussion

### 3.1 NMR Structural Analysis

**Figure 2:**
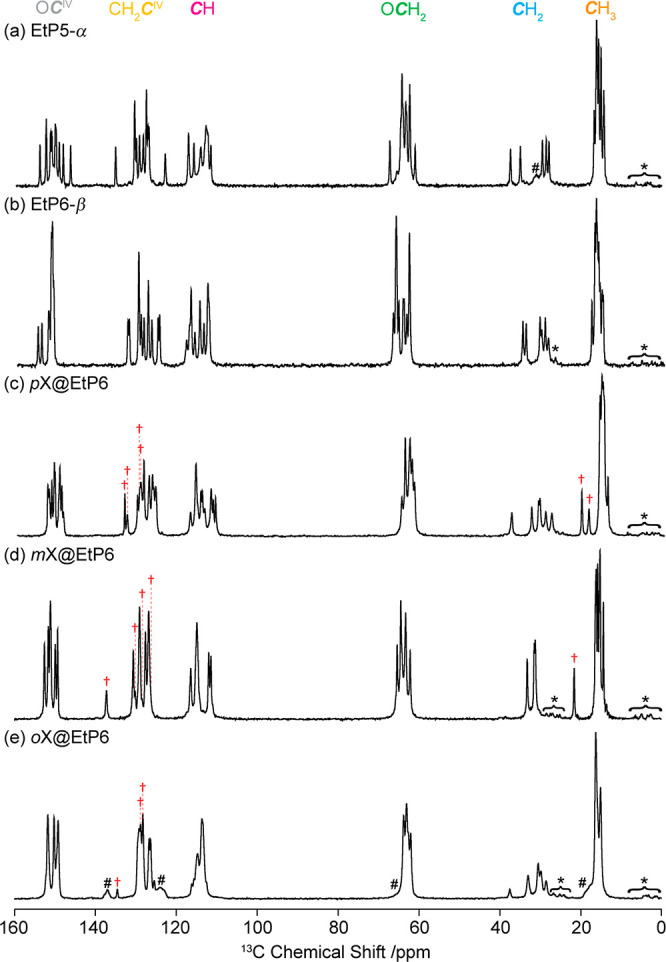
^13^C CP MAS NMR spectra of (a) **EtP5-α**, (b) **EtP6-β**, (c) *p***X@EtP6**, (d) *m***X@EtP6**, and (e) *o***X@EtP6** obtained at a magnetic field of 9.4 T. The spectra for **EtP5-α** and **EtP6-β** are identical to those previously published.^23^ Spectral assignments are given in the figure (see Figure 1) and are obtained from known δ_iso_, ^13^C-edited CP experiments (Figures S8–S10), spectral deconvolution (Figures S11–S13), and 2D PDLF data (see below). The red daggers (†) denote signals arising from the xylene guests. The *C*H_3_ originating from the *o*-xylene guest in (e) is unidentifiable due to spectral broadening and overlapping resonances with the *C*H_3_ signals of the EtP6 host. Asterisks (*) and hashes (#) denote spinning sidebands and amorphous impurities, respectively.

**Figure 3:**
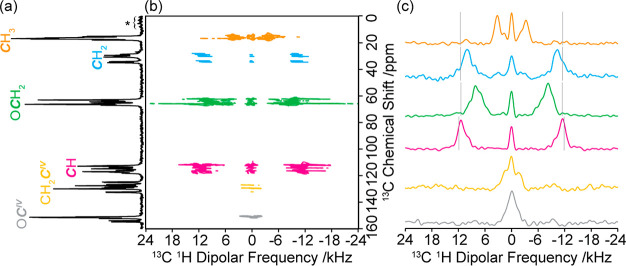
(a) ^13^C CP MAS spectrum, (b) PDLF spectrum and (c) selected site-specific ^13^C ^1^H dipolar spectra for guest-free **EtP6-β**. Spectral assignments are given in the figure and correspond to those previously published.^23^ The data presented above was obtained at 298 K and 9.4 T. ⟨*d*_CH_⟩ is measured using the outer singularities of the dipolar coupling spectra as highlighted in the Experimental Section. Vertical light gray lines indicate the static limit dipolar coupling constants *d*_CH_ calculated from eq 1 and the computed CH distances obtained at the DFT level on the various conformers identified by CSP.^23^ Asterisks (*) denote spinning sidebands.

The ^13^C CP MAS NMR spectra of guest-free **EtP5-α** and **EtP6-β** (Figure 2a,b,^23^Table 1) collected under MAS at 12.5 kHz and at a magnetic field of 9.4 T are extremely well resolved with full width at half-maximum lines typically around 30 Hz (or 0.3 ppm at 9.4 T), in agreement with the excellent crystallinity of these samples. Each different chemical subgroup can be readily assigned, and the remarkable resolution obtained enables the observation of all nonequivalent magnetically distinct carbon atoms in the asymmetric unit cells.^23^ The ^13^C CP MAS NMR spectra of all xylene-adsorbed EtP6 adducts (Figure 2c–e) are all different from **EtP6-β** and from each other, as previously identified by CSP of the molecular conformational space. Therefore, the spectral identification of the xylenes resonances (red daggers in Figure 2c–e, Table 1) is not straightforward and is obtained based on comparisons with well-established isotropic chemical shift (δ_iso_) values,^57−59^^13^C-edited NMR experiments (Figures S8–S10) employing CP steps of various contact times, including their spectral deconvolution (Figures S11–S13), and the existence of CH dipolar couplings (Figures 3 and S20–S22). A detailed discussion on the spectral assignment of the xylenes-adsorbed EtP6 is available in Section S7 of the SI.

**Table 1:** ^13^C NMR Assignments, ^13^C Isotropic Chemical Shifts δ_iso_ from Spectral Deconvolution, Calculated Static Dipolar Coupling Constants *d*_CH_, Experimentally Found Motional Averaged Dipolar Coupling Constants ⟨*d*_CH_⟩, and Order Parameters ⟨*S*_CH_⟩ for Protonated Carbons in **EtP5-α**, **EtP6-β**, *p***X@EtP6**, *m***X@EtP6**, and *o***X@EtP6** at 298 and 243 K

			298 K, 9.4 T		243 K, 14.1 T	
assignment	^13^C δ_iso_ (ppm)^a^	*d*_CH_ (kHz)^b^	⟨*d*_CH_⟩ (kHz)^c^	⟨*S*_CH_⟩^d^	⟨*d*_CH_⟩ (kHz)^c^	⟨*S*_CH_⟩^d^
**EtP5-α**						
*C*H_3_	14–17	–23.1	–7.2 ± 0.5	0.31 ± 0.02	–7.5 ± 0.5	0.32 ± 0.02
*C*H_2_	28–38	–23.0	–23.3 ± 0.8	1.01 ± 0.04	–22.8 ± 0.8	0.98 ± 0.04
O*C*H_2_	61–68	–22.8	–18.4 ± 0.7	0.81 ± 0.03	–19.8 ± 0.7	0.87 ± 0.03
*C*H	111–124	–23.8	–23.8 ± 0.8	1.00 ± 0.03	–23.4 ± 0.8	0.98 ± 0.04
**EtP6-β**						
*C*H_3_	14–18	–23.1	–7.2 ± 0.5	0.31 ± 0.02	–7.2 ± 0.5	0.31 ± 0.02
*C*H_2_	27–35	–23.0	–22.4 ± 0.8	0.97 ± 0.04	–21.7 ± 0.8	0.94 ± 0.04
O*C*H_2_	62–67	–22.8	–18.1 ± 0.7	0.79 ± 0.03	–18.3 ± 0.7	0.80 ± 0.03
*C*H	111–118	–23.8	–23.9 ± 0.8	1.00 ± 0.04	–23.4 ± 0.8	0.98 ± 0.03
*p***X@EtP6**						
*C*H_3_	13–16	–23.1	–6.9 ± 0.5	0.30 ± 0.02	–7.3 ± 0.5	0.32 ± 0.03
*C*H_3_^e^	18–21	–23.7	–7.0 ± 0.5	0.30 ± 0.03	–7.1 ± 0.5	0.30 ± 0.02
*C*H_2_	27–38	–23.1	–21.6 ± 0.8	0.94 ± 0.04	–21.3 ± 0.8	0.92 ± 0.04
O*C*H_2_	61–65	–22.9	–18.4 ± 0.7	0.80 ± 0.03	–19.2 ± 0.7	0.84 ± 0.03
*C*H	110–118	–23.8	–22.4 ± 0.8	0.94 ± 0.03	–22.5 ± 0.8	0.95 ± 0.04
*C*H^e^	129–130	–23.7	–21.6 ± 0.8	0.91 ± 0.04	–22.6 ± 0.8	0.95 ± 0.04
*m***X@EtP6**						
*C*H_3_	14–17	–23.1	–6.9 ± 0.5	0.30 ± 0.02	–7.0 ± 0.5	0.30 ± 0.02
*C*H_3_^e^	21–22	–24.4	–6.2 ± 0.5	0.25 ± 0.02	–6.7 ± 0.5	0.27 ± 0.03
*C*H_2_	31–34	–23.1	–22.2 ± 0.8	0.96 ± 0.04	–21.2 ± 0.8	0.92 ± 0.04
O*C*H_2_	62–66	–22.8	–19.2 ± 0.7	0.84 ± 0.03	–20.0 ± 0.7	0.88 ± 0.03
*C*H	111–117	–23.7	–23.5 ± 0.8	0.99 ± 0.03	–23.2 ± 0.8	0.98 ± 0.03
*C*H^e^	126–131	–24.0	–22.5 ± 0.8	0.94 ± 0.04	–22.3 ± 0.8	0.93 ± 0.03
*o***X@EtP6**						
*C*H_3_	14–17	–23.1	–7.1 ± 0.5	0.31 ± 0.02	–7.0 ± 0.5	0.30 ± 0.02
*C*H_3_^e^^f^		–23.7				
*C*H_2_	28–38	–23.2	–23.8 ± 0.8	1.03 ± 0.04	–22.2 ± 0.8	0.96 ± 0.04
O*C*H_2_	62–66	–22.7	–18.3 ± 0.7	0.81 ± 0.03	–18.8 ± 0.7	0.83 ± 0.03
*C*H	111–116	–23.9	–23.8 ± 0.8	1.00 ± 0.04	–22.8 ± 0.8	0.96 ± 0.04
*C*H^e^^f^	128–129	–23.6	–17.0 ± 0.7	0.72 ± 0.03	–18.8 ± 0.7	0.80 ± 0.03

a Range of ^13^C δ_iso_ obtained at room temperature are given for each carbon subgroup. Exact δ_iso_ for all individual carbons are provided in Tables S2–S6.

b Static dipolar coupling constants were calculated as described in the text in Section 3.2 and eq 1.

c Only the short-range ⟨*d*_CH_⟩ constants are given (see text for details). Errors are estimated from the uncertainty in the determination of the position of the outer singularities of the ^13^C ^1^H dipolar coupling spectra.

d Estimated errors are calculated from the errors in ⟨*d*_CH_⟩.

e Signals from xylenes.

f Overlapping resonances between the guest and the host in the ^13^C CP MAS NMR spectrum of *o***X@EtP6** prevents spectral assignment of the *C*H_3_ and only allow tentative assignment of the xylene CH carbons.

Variable–temperature ^13^C CP MAS NMR spectra (Figures S14–S18) for all five materials were performed in the 383–100 K temperature range (down to only 243 K for **EtP5-α**). Upon cooling, significantly broader ^13^C NMR resonances are observed at low temperature (e.g., from 30 Hz at 298 K to 60 Hz at 100 K for the *C*H resonance of **EtP6-β** at 14.1 T) as anticipated from the macrocycles being trapped in a variety of conformations and leading to inhomogeneous broadening. There is minimal change in the intensity of the spinning sidebands, which likely indicates that the ^13^C CSA is largely unchanged in the temperature range studied here while also suggesting that accessing ^13^C CSAs is likely not a suitable method to obtain dynamics information in the kHz regime in these materials. There is also no evidence of signal coalescence due to chemical exchange.

Upon heating above 323 K, the ^13^C CP MAS NMR spectrum of **EtP6-β** remains very well resolved and the number of resonances halves (Figure S19) vs spectra at 298 K, indicating a crystal structure of higher symmetry. This change is in agreement with both the DSC data (Figure S2) that shows an endothermic peak at 339 K, and with refined XRD data at 433 K that indicated a transition from triclinic *P*1**EtP6-β** at room temperature to a metastable triclinic *P*1 state at 339 K with a half unit cell volume.^23^ In contrast, the ^13^C CP MAS NMR spectrum of **EtP5-α** up to 383 K remains unchanged upon heating (Figure S14), and no polymorphic transition is observed. No change is also observed in the ^13^C CP MAS NMR spectra of *p***X@EtP6** or *o***X@EtP6** up to 323–330 K (Figures S16 and S18), which is consistent with TGA results that show that the adsorbed xylenes are only lost from the pores at temperatures exceeding about 348 K for *p***X@EtP6**(23) and 340 K for *o***X@EtP6** (Figure S4).

The ^13^C CP MAS NMR spectrum of *m***X@EtP6** at 323 K (Figure S17), however, shows the disappearance of the adsorbed xylene peaks and accounts for *m*-xylene desorption from the pores (Figure S3). Upon cooling this sample back to room temperature, the ^13^C CP MAS NMR spectrum (data not shown) indicates that the material has not returned back to **EtP6-β** as this polymorph is only formed above 433 K.^23^ We ascribe this difference of behaviors between *m***X@EtP6** and *p***X@EtP6**/*o***X@EtP6** to the smaller cavity of the former preventing the *m*-xylene guest (Figure 1) to be fully accommodated in the pores and facilitating this removal upon heating.

### 3.2 Temperature-Dependent Motional Averaged Site-Selectivity in Guest-Free Pillar[*n*]arenes and Xylene-Adsorbed Pillar[6]arenes

Heteronuclear dipolar couplings are dependent on distance and motion,^60^ and the magnitude of this dipole–dipole coupling is given by the following expression for ^13^C ^1^H
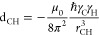
1where *d*_CH_ is the dipolar coupling constant between the ^13^C and ^1^H nuclei in Hz, μ_0_ is the vacuum permittivity, ℏ is the reduced Planck constant, γ_C_ and γ_H_ are the respective gyromagnetic ratios for the ^13^C and ^1^H nuclei, and *r*_CH_ is the distance between the carbon and hydrogen atoms. Motional averaged dipolar couplings ⟨*d*_CH_⟩ can be obtained by two-dimensional (2D) proton detected local field (PDLF)^43,44,61^ experiments that correlate the ^13^C isotropic chemical shifts with their corresponding ^13^C ^1^H dipolar spectra, providing site-selective heteronuclear dipolar coupling constants (see the Experimental Section for further details).

The room-temperature ^13^C CP MAS NMR spectrum of **EtP6-β** is given in Figure 3a with the corresponding 2D PDLF spectrum (Figure 3b) showing dipolar coupling for all protonated carbons as expected (Figure 3c) and allowing the corresponding ^13^C ^1^H dipolar spectra to be extracted at each ^13^C shifts which revealed significant ⟨*d*_CH_⟩ differences between each carbon subgroup. For example, smaller ⟨*d*_CH_⟩ of −7.2 ± 0.5 and −18.1 ± 0.7 kHz are obtained for the *C*H_3_ and O*C*H_2_ carbons of the ethoxy group, respectively, while larger values of −22.4 ± 0.8 and −23.9 ± 0.8 kHz are extracted for the *C*H_2_ and *C*H carbons of the pillar[6]arene backbone ring (Tables 1 and S3). While no dipolar coupling splitting is apparent for the quaternary O*C*^IV^ carbons, partially resolved small couplings of −2.9 ± 0.3 kHz are obtained for the CH_2_*C*^IV^ carbons and is likely due long-range through space coupling to the nearby methylene *C*H_2_ ring group. Similar long-range dipolar couplings (−4.0 ± 0.3 and −6.1 ± 0.5 kHz) can also be observed for the *C*H_3_ and *C*H_2_ environments, respectively, which arise from spatial proximity with protons on the nearby carbons.

**Figure 4:**
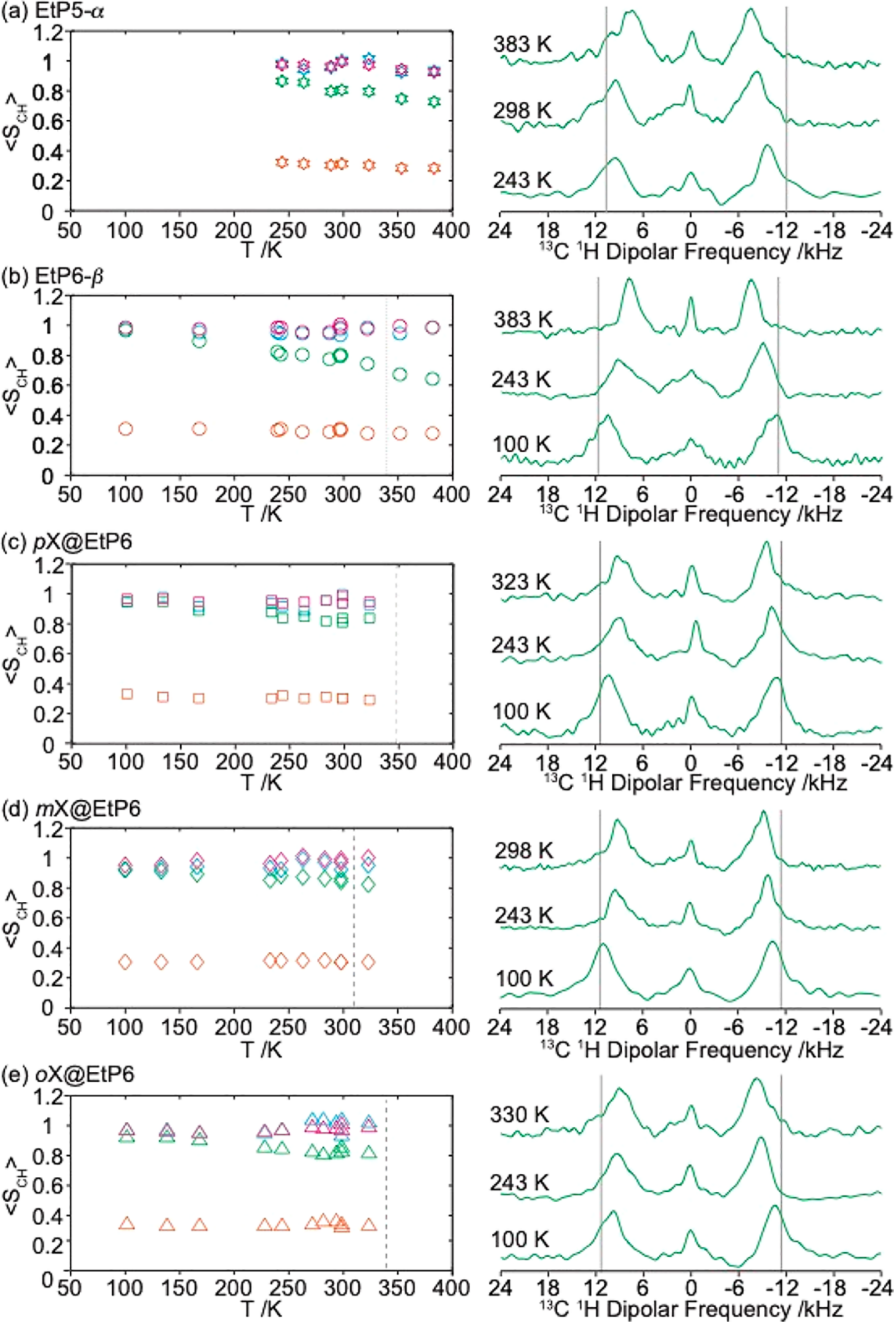
(Left) Temperature dependency of the motional averaged CH dipolar coupling order parameters ⟨*S*_CH_⟩ and (right) selected ^13^C ^1^H dipolar coupling spectra of the O*C*H_2_ signals at various temperatures for (a) guest-free **EtP5-α** (stars), (b) guest-free **EtP6-β** (circles), (c) *p***X@EtP6** (squares), (d) *m***X@EtP6** (diamonds), and (e) *o***X@EtP6** (triangles). The different carbon subgroups can be identified with the following color coding for *C*H_3_ (orange), *C*H_2_ (light blue), O*C*H_2_ (green), and *C*H (pink) (Figure 1). Data recorded at room temperature have been collected at both 9.4 and 14.1 T. Error bars in ⟨*S*_CH_⟩ (Δ*S*_CH_) are consistently smaller than 0.04 and are obtained from estimated errors in the determination of ⟨*d*_CH_⟩ and small variations in the dipolar coupling values across one carbon subgroup (see Figure S7); these errors are less than the symbol size. Data below 243 K were not recorded for **EtP5-α**. The dotted line in (b) indicates a polymorphic transition in **EtP6-β** from triclinic P1 to a metastable triclinic *P*1 state with higher symmetry at 339 K (see Figure S2). Dashed lines in (c) and (d) represent the onset temperatures at which xylenes are lost as identified by the TGA data for *p***X@EtP6**(23) and *o***X@EtP6** (Figure S4) and both TGA data (Figure S3) and changing NMR spectrum (Figure S17) for *m***X@Et6**. Vertical light gray lines in the dipolar coupling spectra indicate the static limit dipolar coupling constants *d*_CH_.

Motion can be quantified by a site-specific order parameter ⟨*S*_CH_⟩ (eq 2, Table 1 and Figure 4) that compares ⟨*d*_CH_⟩ with the static limit dipolar coupling constants *d*_CH_ in the absence of motion and ranges from 0 for isotropic motion to 1 for a rigid system
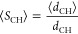
2⟨S_CH_⟩ obtained for each carbon subgroup in **EtP6-β** are found to be 0.31 ± 0.02 for *C*H_3_, 0.97 ± 0.04 for *C*H_2_, 0.79 ± 0.03 for O*C*H_2_, and 1.00 ± 0.04 for *C*H (Table 1) at room temperature. There is therefore no (or limited) motion for the *C*H_2_ and *C*H carbons situated in the arene core of the pillar[*n*]arene ring. However, both *C*H_3_ and O*C*H_2_ carbons in the ethoxy group show motional averaging caused by dynamics which is ascribed to rotational and librational motions of these carbons. While this effect is fairly small for the O*C*H_2_ carbon (⟨*S*_CH_⟩ = 0.79), motion is particularly pronounced for the *C*H_3_ group which ⟨*d*_CH_⟩ is approximately one-third of the *d*_CH_ yielding ⟨*S*_CH_⟩ = 0.31 and indicates an increase of motion further away from the arene core.

The temperature dependency of ⟨*S*_CH_⟩ was obtained by measuring site selective ⟨*d*_CH_⟩ for **EtP5-α** from 383 K down to 243 K (Figure 4a, Table 1) and for **EtP6-β** over an extended temperature range from 383 K down to 100 K (Figure 4b). In **EtP6-β** (and for all samples), the ⟨*S*_CH_⟩ values for the *C*H_3_ groups remain largely constant at 0.31 ± 0.02, indicating that this group still possesses significant motion even at 100 K. This is consistent with temperatures lower than 100 K required to “freeze” the rapid 3-site hopping motion of *C*H_3_ in various biomolecules.^62−64^ In contrast, the ⟨*S*_CH_⟩ values of the O*C*H_2_ increase significantly upon cooling from 0.79 ± 0.03 at 298 K to 0.95 ± 0.03 at 100 K, supporting reduction in motion and lower flexibility by the pillar[*n*]arene at lower temperatures. In **EtP5-α**, while the room temperature ⟨*S*_CH_⟩ values for the *C*H_3_, *C*H_2_, and *C*H carbon subgroups are virtually identical to those determined for **EtP6-β** (Table 1), a difference was observed for the O*C*H_2_ group upon cooling. An increase in ⟨*d*_CH_⟩ from −18.3 ± 0.7 kHz in **EtP6-β** to −19.8 ± 0.7 kHz in **EtP5-α** is observed as evidenced by larger splitting of the outer singularities in the ^13^C ^1^H dipolar spectra at 243 K (Figure 4b) and results in slightly larger ⟨*S*_CH_⟩ values in **EtP5-α** (0.87 ± 0.03) than in **EtP6-β** (0.80 ± 0.03). Similarly, at higher temperature (383 K), the ^13^C ^1^H dipolar coupling spectra of the O*C*H_2_ group yield larger ⟨*d*_CH_⟩ values (−16.6 ± 0.7 and −14.5 ± 0.6 kHz) and smaller ⟨*S*_CH_⟩ values (0.73 ± 0.03 vs 0.64 ± 0.03) in **EtP5-α** than in **EtP6-β**, respectively. This indicates more restricted motion and increased hindrance which is likely due to the reduced void space of the smaller **EtP5-α** cavity versus **EtP6-β**.

Variable-temperature 2D PDLF NMR experiments were also recorded on the three guest-adsorbed xylene adducts in EtP6 to access ⟨*d*_CH_⟩ and ⟨*S*_CH_⟩ (Table 1, Figure 4c,d,e for *p***X@EtP6**, *m***X@EtP6**, and *o***X@EtP6**, respectively). There, the trends are largely similar to **EtP6-β** with temperature independent ⟨*S*_CH_⟩ around 1 for the *C*H_2_ and *C*H carbons in the pillar[*n*]arene core, around 0.3 for the *C*H_3_, and increasing toward 1 for the O*C*H_2_ group as temperatures are lowered into the static regime. Although the room temperature ⟨*S*_CH_⟩ values for the *C*H_3_, *C*H_2_, and *C*H carbons are within error of each other for **EtP6-β** and the xylene-adsorbed adducts, there is a slight increase in the room temperature ⟨*S*_CH_⟩ values obtained for the O*C*H_2_ group in **EtP6-β**/*o***X@EtP6**/*p***X@EtP6** vs *m***X@EtP6** (Table 1). This small difference is enhanced further upon cooling to 243 K, and the data therefore seems to suggest marginally slower dynamics of the O*C*H_2_ group in *m***X@EtP6** than in **EtP6-β**, *o***X@EtP6**/*p***X@EtP6**. In contrast to the latter two phases, the xylene in *m***X@EtP6** lies on top of the EtP6 host rather than within the void space, as illustrated in Figure 1; therefore, the interaction of the *m*-xylene with the protruding ethoxy groups is likely to cause slower dynamics, at least for the O*C*H_2_ subgroup. These experiments therefore highlight small change in structure flexibility between guest-free and guest-adsorbed EtP6 assemblies.

The room-temperature PDLF data on the three xylene adducts (Figures S20–S22) also partially resolved the dipolar coupling observed in the xylenes themselves. While the corresponding ⟨*d*_CH_⟩ for the xylene *C*Hs in *p***X@EtP6** and *m***X@EtP6** indicate limited motion with ⟨*S*_CH_⟩ values found in the 0.91–0.94 ± 0.04 range (Table 1), the xylene CHs in *o***X@EtP6** show considerably more motion with smaller ⟨*S*_CH_⟩ values of 0.72 ± 0.04 at 298 K. This indicates that the *o*-xylene has a significant amount of spatial freedom to allow for mobility and that the *C*H and *C*H_3_ motion of the xylene is not completely limited upon loading into the EtP6 cavity at room temperature.

### 3.3 Host–guest Interaction Probed by Dipolar Coupling in Xylene-Adsorbed Pillar[6]arenes

**Figure 5:**
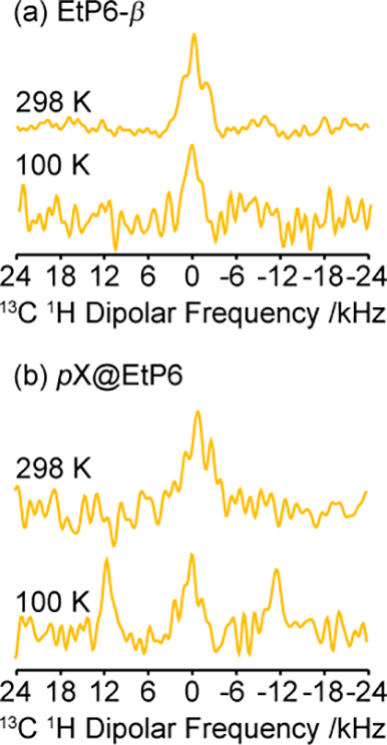
Comparison of selected CH_2_*C*^IV 13^C ^1^H dipolar spectra for (a) **EtP6-β** and (b) *p***X@EtP6** obtained at 298 K and 9.4 T and at 100 K and 14.1 T. The polarization transfer to ^13^C during the PRESTO block of 2D PDLF sequence was optimized for maximum signal on the protonated resonances (see Section 2.2), which accounts for the signal-to-noise of these quaternary carbon resonances.

No large dipolar coupling is observed at room or low temperatures for the quaternary carbons of either xylenes or pillar[6]arene host as expected (Figures 5a and S20–S23); however, surprisingly, upon cooling *p***X@EtP6** to 100 K, strong dipolar couplings of −23.4 ± 0.8 kHz were observed for the CH_2_*C*^IV^ carbon (Figures 5b and S24c). These couplings in *p***X@EtP6** do not originate from either of the *C*Hs in the xylene (at 129.4 and 129.8 ppm) or a long-range interaction in the **EtP6** architecture (no coupling is observed in the CH_2_*C*^IV^ of **EtP6-β** as revealed in Figure 3 at room temperature and Figure S23 at 100 K) but rather from the CH_2_*C*^IV^ carbons (125–131 ppm). Therefore, this coupling was ascribed to intermolecular heteronuclear dipolar coupling between the quaternary CH_2_*C*^IV^ carbon of the EtP6 host and protons of *p*-xylene identifying EtP6 *p*-xylene spatial interaction and strong host–guest interaction. These results are in sharp contrast to the 100 K PDLF data for *m***X@EtP6** and *o***X@EtP6** adducts (Figures S25 and S26, respectively) for which no coupling is observed for CH_2_*C*^IV^s suggesting an absence of host–guest interaction or that the coupling is still averaged out at 100 K.

These results strongly support the structures illustrated in Figure 1. In particular, in *p***X@EtP6**, *p*-xylene is located in the center of the EtP6 cavity which is stabilized by strong π–π stacking with two aromatic rings from EtP6 (Figure 1c), yielding strong ⟨*d*_CH_⟩ between the CH_2_*C*^IV^ carbons of the pillar[6]arene backbone with *p*-xylene protons. It is also likely that this coupling arises preferentially from the aromatic protons of *p*-xylene rather than the methyl protons due to methyl group rotation as discussed above. In *o***X@EtP6**, similar rotational dynamics prevent coupling of the methyl protons of *o*-xylene located inside the cavity to the EtP6 backbone while the aromatic protons are positioned outside the cavity (Figure 1e) from which a small static dipolar coupling would only be expected (0.44 kHz based on the smallest 4.1 Å distance with the EtP6 CH_2_*C*^IV^ carbon). The small EtP6 cavity in *m***X@EtP6** is too small to host *m*-xylene (Figure 1d), resulting in this xylene to be excluded and the absence of dipolar coupling interaction with EtP6.

### 3.4 Temperature-Dependent Relaxation Studies of Guest-Free and -Absorbed Pillar[*n*]arenes

*T*_1_ relaxation is a measure of the time for the spin population to recover to equilibrium after a perturbation and is mediated by fluctuations of the local magnetic fields, as quantified by the correlation times of the motion τ_c_ with corresponding frequencies τ_c_^–1^ on the order of the Larmor frequency, i.e., MHz. Site-specific ^13^C spin–lattice relaxation rates *T*_1_^–1^ for all carbons have been obtained versus temperatures in the 383–243 K and 298–100 K temperature range at 9.4 T (Figures S27) and 14.1 T (Figures S28), respectively, for **EtP6-β**, all xylene-adsorbed EtP6 adducts and **EtP5-α** (data only available at 9.4 T for this phase). ^1^H *T*_1_^–1^ were also obtained (Figures S27–S29) and suggest the same similar motional process likely due to the lack of resolution (for a discussion of the ^1^H data, Section S10 of the SI). Illustration of the ^13^C *T*_1_s obtained at room temperature and 9.4 T are given in Tables S2–S6 for **EtP5-α**, **EtP6-β**, *p***X@EtP6**, *m***X@EtP6**, and *o***X@EtP6**, respectively, and we have chosen to give a single *T*_1_ value (with associated errors) for each carbon subgroup as these are within errors of each other. The following general trend is observed in all of the guest-free and xylene-adsorbed pillar[*n*]arenes: the *C*H_3_ group has the shortest *T*_1_ (approximately 2 s at room temperature) of all the carbon environments, as it is well-known that methyl groups are relaxation sinks due to their facile three-site hopping motions and efficient ^13^C ^1^H heteronuclear dipole–dipole coupling relaxation; the ^13^C *T*_1_ of the O*C*H_2_ moieties are also relatively short (approximately 20–40 s) and likely due to rotation around the O–C bond; these *T*_1_ are in contrast with the ones of the *C*H_2_/*C*H groups that are in the 10^2^ s range and suggest limited motional freedom and rigidity of these pillar[6]arene core groups; O*C*^IV^ and CH_2_*C*^IV^ carbons yield the longest *T*_1_ as the dominant relaxation mechanism of CSA (see below) is less efficient than dipolar coupling to ^1^H for these nonprotonated carbons. Note that upon loading of *p*- and *m*-xylene, the O*C*H_2_ group shows an increase in *T*_1_ at room temperature, suggesting that guest addition lowers the flexibility of the pillar[*n*]arenes. *o***X@EtP6** shows a reduction in nearly all *T*_1_ in comparison to **EtP6-β**; however, this is likely attributed to the more amorphous nature of this material.

The ^13^C *T*_1_^–1^ rates for each carbon subgroup in **EtP5-α**, **EtP6-β**, and the xylene-adsorbed adducts typically increase with increasing temperatures (Figure S28b), pass through maxima at 165–168 K (at 14.1 T) for the majority of resonances (excluding the *C*H_2_ in *p***X@EtP6**, *C*H in *o***X@EtP6** and the *C*H_3_, *C*H_2_, *C*H, and O*C*^IV^ in *m***X@EtP6**), and then decrease. At these *T*_1_^–1^ maxima, the motion is near the ^13^C Larmor frequency ω_0,C_ (in rad·s^–1^) with the following expression eq 3 being satisfied^65^
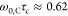
3leading to a τ_c_ value of 6.5 × 10^–10^ s for these materials.

Assuming negligible contribution from spin-rotation and scalar coupling relaxation mechanisms, ^13^C *T*_1_^–1^ rates can generally be expressed (eq 4) 
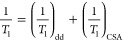
4as the sum of both ^13^C ^1^H heteronuclear dipolar coupling (eq 5) 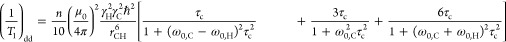
5and ^13^C CSA relaxation (eq 6)^65,66^
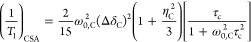
6mechanisms with *n* the number of protons attached to ^13^C, Δδ_C_ the (reduced) anisotropy (sensitivity of the chemical shift interaction to the orientation), and η_C_ asymmetry parameter (deviation from axial symmetry) of the second rank ^13^C chemical shift tensor with principal components δ_11_, δ_22_ and δ_33_ as defined in Section S12 of the SI (all other terms are defined above). The local magnetic fields fluctuation term of the CSA expression is magnetic field dependent and proportional to the square of the Larmor frequency and anisotropy.

^13^C relaxation generally arises from ^13^C–^1^H heteronuclear dipole–dipole coupling for protonated carbons with small CSA, i.e., *C*H_3_, O*C*H_2_, and *C*H_2_, and from ^13^C CSA for quaternary aromatic carbons, i.e., O*C*^IV^ and CH_2_*C*^IV^, as confirmed by comparing the magnitude of the local dipolar and CSA magnetic fields term in eqs 5 and 6. For example, in *m***X@EtP6** (similar observations were made on the other materials), the calculated local dipolar magnetic fields term for *C*H_3_ (6 × 10^9^ s^–2^) is 2 orders of magnitude larger than the calculated CSA term (8 × 10^7^ s^–2^ at 14.1 T assuming a typical ^13^C Δδ_C_ for this carbon of 25 ppm),^67^ while for O*C*^IV^, the CSA term largely dominates even at the lower magnetic field (1 × 10^9^ s^–2^ at 9.4 T with a ^13^C Δδ_C_ of −142 ppm vs 4 × 10^7^ s^–2^ for dipolar). However, for the remaining aromatic *C*H sites, ^13^C relaxation derives from cross terms between dipolar and CSA interactions^68^ as both local magnetic field contributions are comparable (2 × 10^9^ s^–2^ for dipolar vs 1–3 × 10^9^ s^–2^ at 9.4–14.1 T for CSA using an aromatic CH with a ^13^C Δδ_C_ of −147 ppm)^69^ and is further suggested by the slight magnetic field dependency of the *T*_1_.

**Figure 6:**
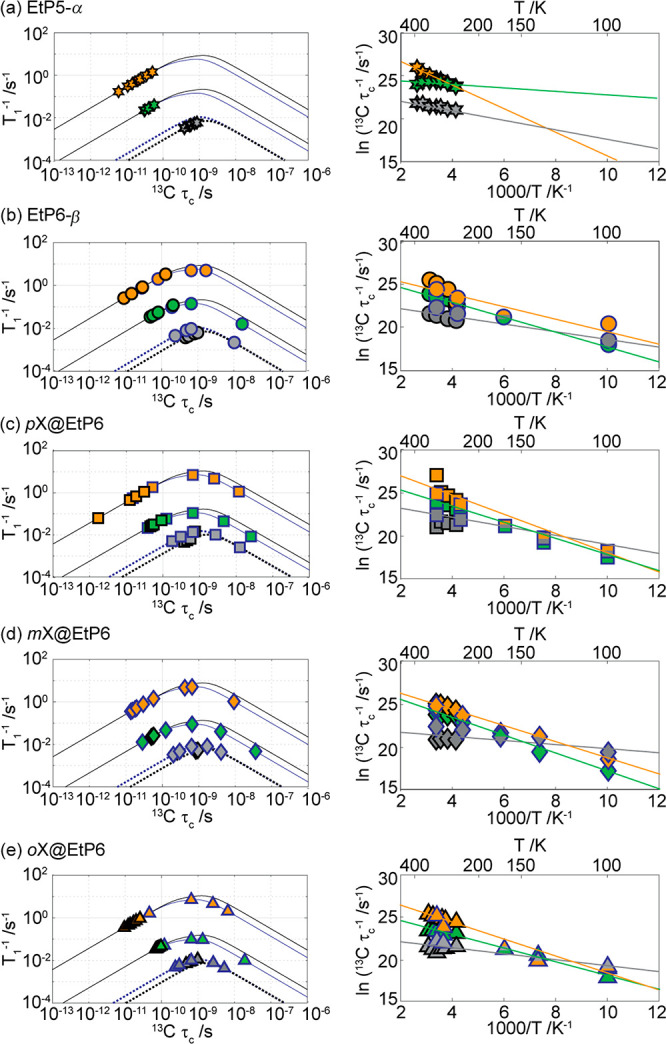
(Left) ^13^C spin–lattice relaxation rates *T*_1_^–1^ against correlation times τ_c_ and (right) corresponding ^13^C correlation frequencies τ_c_^–1^ Arrhenius plots. Data shown in black and blue outlines were obtained at 9.4 and 14.1 T, respectively, for (a) guest-free **EtP5-α** (stars), (b) guest-free **EtP6-β** (circles), (c) *p***X@EtP6** (squares), (d) *m***X@EtP6** (diamonds), and (e) *o***X@EtP6** (triangles). Selected carbon subgroups have been plotted here with the following color coding for *C*H_3_ (orange), O*C*H_2_ (green), and O*C*^IV^ (gray) (Figure 1) while plots giving the three other carbons are given in Figure S30. The associated errors are smaller than the symbol sizes. In the left panels, the solid (−) lines are those obtained from a dipolar coupling relaxation mechanism (eq 5) for *C*H_3_ (orange) and O*C*H_2_(green) and the dotted (··) lines from a CSA relaxation mechanism (eq 6) for O*C*^IV^(gray) at both fields, using the experimentally determined local magnetic fields terms (values from **EtP6-β** were used for **EtP5-α** as no *T*_1_ minimum was found in the temperature range studied). A *T*_1_ minima was found for O*C*^IV^ in *o***X@EtP6** in the temperature range studied at 9.4 T; therefore, this data was used to extract correlation times and is plotted for this series. In the right panels, the lines are fit to the experimental data using the Arrhenius equation.

^13^C *T*_1_^–1^ maxima and dominant relaxation mechanism(s) allow experimental access to the local magnetic fields term by combining eq 3 and either eq 5 (for heteronuclear dipolar coupling relaxation), eq 6 (for CSA relaxation), or eq 4 (for both mechanisms). For example, in keeping with *m***X@EtP6**, the experimentally determined local dipolar magnetic fields term for *C*H_3_ (3 × 10^9^ s^–2^) compares well with the calculated value (6 × 10^9^ s^–2^). These equations were then used to obtain τ_c_ for all materials (*C*H_3_, O*C*H_2_, and O*C*^IV^ in Figure 6a–e; *C*H_2_, *C*H, and CH_2_*C*^IV^ in Figure S30a–e) and the room-temperature τ_c_ are the shortest for the *C*H_3_ and O*C*H_2_ groups supporting motion. The temperature dependence of the correlation frequencies τ_c_^–1^ was subsequently modeled with an Arrhenius equation of the form
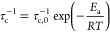
7with τ_c,0_^–1^, *E*_a_, and *R* the attempt frequency, activation energy of the thermally activated motional process, and universal gas constant, respectively, and are given in Table 2 for ^13^C and Table S7 for ^1^H. The *E*_a_ for *C*H_3_ in **EtP6-β** (6 kJ mol^–1^) is significantly smaller than in **EtP5-α** (11 kJ mol^–1^) and is likely due to the smaller ring size of the latter hindering molecular rotation. Upon addition of any guest of **EtP6-β**, the *E*_a_ for *C*H_3_ increases to 8–10 kJ mol^–1^ which suggests restricted motion caused by their spatial proximities. No significant difference is however observed between the different guest-adsorbed materials or for O*C*H_2_ groups. There, much smaller changes in *T*_1_ and τ_c_ times are measured and would therefore indicate that, within the temperature range probed, all materials experience the same motional processes.

**Table 2:** Comparison of the Attempted Frequencies τ_c,0_^–1^ and Activation Energy Barriers *E*_a_ of Guest-Free **EtP5-α**, Guest-Free **EtP6-β**, *p***X@EtP6**, *m***X@EtP6**, and *o***X@EtP6** Obtained from the Arrhenius Plots of the ^13^C Correlation Frequencies τ_c_^–1^

carbon subgroup	τ_c,0_^–1^ (s^–1^)	*E*_a_ (kJ mol^–1^)^a^
**EtP5-α**^b^		
*C*H_3_	5 × 10^12^	11
*C*H_2_	6 × 10^10^	5
O*C*H_2_	6 × 10^10^	2
*C*H	4 × 10^10^	5
CH_2_*C*^IV^	5 × 10^12^	4
O*C*^IV^	4 × 10^10^	5
**EtP6-β**		
*C*H_3_	4 x10^11^	6
*C*H_2_	6 × 10^10^	5
O*C*H_2_	2 × 10^11^	7
*C*H	3 × 10^10^	4
CH_2_*C*^IV^	8 × 10^9^	3
O*C*^IV^	1 × 10^10^	4
*p***X@EtP6**		
*C*H_3_	5 × 10^12^	10
*C*H_3_^c^	5 × 10^12^	3
*C*H_2_	2 × 10^11^	7
O*C*H_2_	7 × 10^11^	8
*C*H	7 × 10^10^	5
CH_2_*C*^IV^	1 × 10^10^	4
*C*H^c^^d^^e^		
O*C*^IV^	2 × 10^10^	4
*m***X@EtP6**		
*C*H_3_	2 × 10^12^	8
*C*H_3_^c^	4 × 10^12^	1
*C*H_2_	5 × 10^10^	5
O*C*H_2_	1 × 10^12^	9
*C*H	8 × 10^9^	3
CH_2_*C*^IV^	7 × 10^9^	3
*C*H^c^^d^^e^		
O*C*^IV^	5 × 10^9^	2
*o***X@EtP6**		
*C*H_3_	2 × 10^12^	8
*C*H_3_^c^^d^		
*C*H_2_	5 × 10^9^	2
O*C*H_2_	2 × 10^11^	7
*C*H	3 × 10^9^	2
CH_2_*C*^IV^	5 × 10^9^	3
*C*H^c^^d^^e^		
O*C*^IV^	7 × 10^9^	3

a Errors are in the order of 1 kJ mol^–1^.

b Data in the 383–243 K temperature range only available.

c Denotes signals from xylenes.

d Overlapping resonances between guest and host in the ^13^C CP MAS NMR spectra prevent measurement of *T*_1_ times.

e Signal to noise of some of the signals are also too weak for accurate determination of *T*_1_ times.

τ_c_ were also extracted for the methyl groups of the xylene guests in both *p***X@EtP6** and *m***X@EtP6**, and the temperature dependency of their frequencies was used to extract *E*_a_ values (Figure S31a,b). Activation energies are small (1–3 kJ mol^–1^, Table 2) and significantly less than the ones determined for the *C*H_3_ groups of the host (10 kJ mol^–1^ in *p***X@EtP6**; 8 kJ mol^–1^ in *m***X@EtP6**), indicating that the *C*H_3_ groups have significantly higher degree of motion in the xylenes than pillar[6]arene. Additionally, further comparison between the xylene *C*H_3_’s in *p***X@EtP6** vs *m***X@EtP6** reveals higher *E*_a_ in the former and supports the xylene location inside the arene core.

## 4 Conclusions

We employed variable-temperature multinuclear NMR experiments to provide detailed understanding of the dynamics in guest-free perethylated pillar[*n*]arene (*n* = 5,6) and xylenes-adsorbed pillar[6]arenes. Site-selective ^13^C ^1^H dipolar spectra, enabled by the highly resolved ^13^C CP MAS NMR spectra, permit the quantification of order parameters that reveal differential dynamics properties. Protruding carbons were found to have faster dynamics than those in the core, while the larger void size of **EtP6-β** than **EtP5-α** results in a less restricted OCH_2_ motion. ^13^C ^1^H dipolar spectra also identified spatial proximity in *p***X@EtP6**, not detected in *o***X@EtP6** and *m***X@EtP5**, demonstrating significantly strong π–π stacking of *p*-xylene located in the center of the void validating structural models. Temperature-dependent correlation frequencies from relaxation times measurements tentatively suggest *o***X@EtP6** to have the largest size conformation and show extensive motional dynamics of the perethylated and xylene methyl carbons.

This work demonstrates the capture of structural transformations resulting from host–guest interactions and motional effects in adaptative pillar[*n*]arene materials, which could have implications for processes such as competitive loading, molecular separation, and drug release. This adds to our understanding of motion in flexible molecular solid state systems and opens up new perspectives in the rational design of materials with enhanced physical properties.

## Supporting Information Available

NMR data, additional method details, synthesis details and their supporting data (PDF)
