# Supplementary material for: Dynamics in Flexible Pillar[n]arenes Probed by Solid-State NMR
Source: J Phys Chem C Nanomater Interfaces. 2021 Jun 15;125(24):13370–81. doi: 10.1021/acs.jpcc.1c02046 (PMC8237263; doi:10.1021/acs.jpcc.1c02046)
Supplement: Supplementary file 1 — jp1c02046_si_001.pdf [file jp1c02046_si_001.pdf]

Supporting Information for:

Dynamics in Flexible Pillar[n]arenes Probed by Solid-state NMR

Ashlea R. Hughes,[a] Ming Liu,[a, b] Subhradip Paul,[c] Andrew I. Cooper[a, b] and Frédéric Blanc[*] [a, d]

[a] Department of Chemistry, University of Liverpool, Crown Street, Liverpool, L69 7ZD.

[b] Materials Innovation Factory, University of Liverpool, 51 Oxford Street, Liverpool, L7 3NY, United Kingdom.

[c] Nottingham DNP MAS NMR Facility, Sir Peter Mansfield Imaging Centre, University of Nottingham, NG7 2RD, United Kingdom.

[d] Stephenson Institute for Renewable Energy, University of Liverpool, Crown Street, Liverpool, L69 7ZD, United Kingdom.

*Corresponding Author: Frédéric Blanc, E-mail: frederic.blanc@liverpool.ac.uk

Table of Contents

S1. Synthesis scheme for perethylated pillar[n]arenes (n = 5, EtP5; n = 6, EtP6)

S2. Powder X-ray diffraction (XRD) data

S3. Differential Scanning Calorimetry (DSC) data

S4. ThermoGravimetric Analysis (TGA) data

S5. NMR pulse programs

S6. Scaling factor determination

S7. ^{13}C NMR spectra assignments

S8. Variable temperature ^{13}C NMR spectra

S9. Additional proton detected local field NMR spectra

S10. Additional ^{1}H and ^{13}C spin lattice relaxation rates T_1^{-1} vs. temperatures

S11. Very high field and variable temperature ^{1}H NMR spectra

S12. Expression of the ^{13}C chemical shift tensors

S13. References

S1. Synthesis scheme for perethylated pillar[n]arenes (n = 5, EtP5; n = 6, EtP6)

EtP5 and **EtP6** were synthesised by reaction of 1,4-diethoxybenzene with paraformaldehyde in the presence of boron trifluoride diethyl etherate followed by separation from the other pillar[n]arenes, EtPn, by column chromatography (**Scheme S1**).[1] **EtP5-α** was obtained by recrystallisation from tetrahydrofuran followed by a drying step while **EtP6-β** was prepared by heating the as synthesis EtP6. Subsequently, both ***p*X@EtP6** and ***m*X@EtP6** were prepared using the solid-vapour diffusion method and ***o*X@EtP6** *via* solvent evaporation as recently published.[2] The PXRD patterns of all materials are given in **Figure S1** and agree with the reported ones.[2]

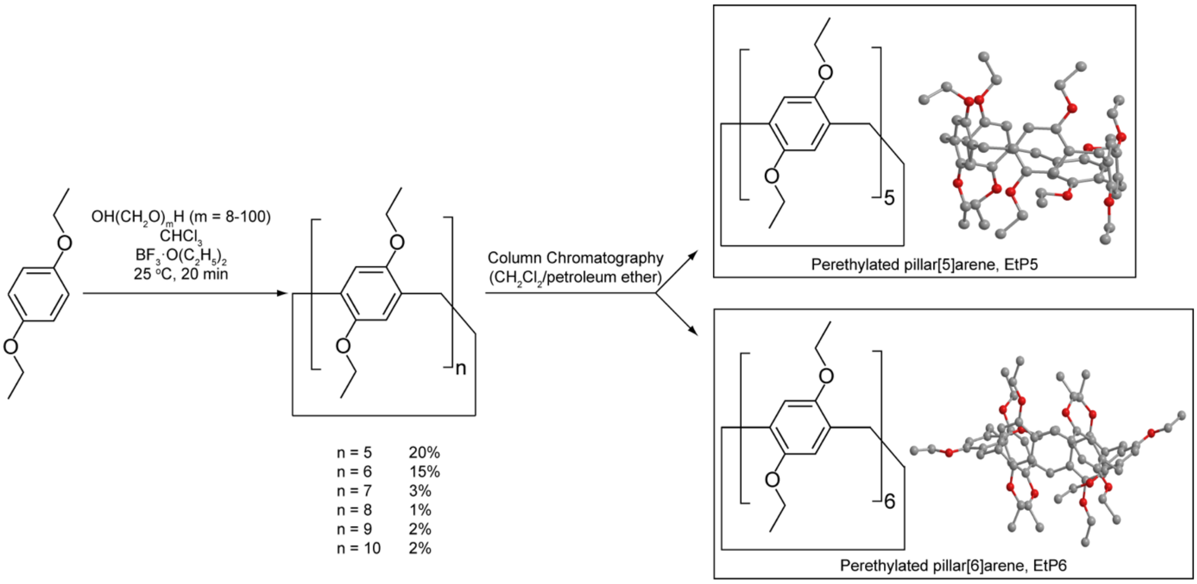

Scheme S1. Synthesis of perethylated pillar[n]arenes from 1,4-diethoxybenzene and paraformaldehyde.[1] The perethylated pillar[n]arenes were purified by column chormatograph to separate EtP5 and EtP6. The crystal structures of the pillar[n]arenes (5 and 6) are denoted by 'ball and stick' models, with carbons shown in grey, oxygens in red and protons omitted for clarity. Resulting yields obtained *via* this synthesis method are also given.[1]

S2. Powder X-ray diffraction (PXRD) data

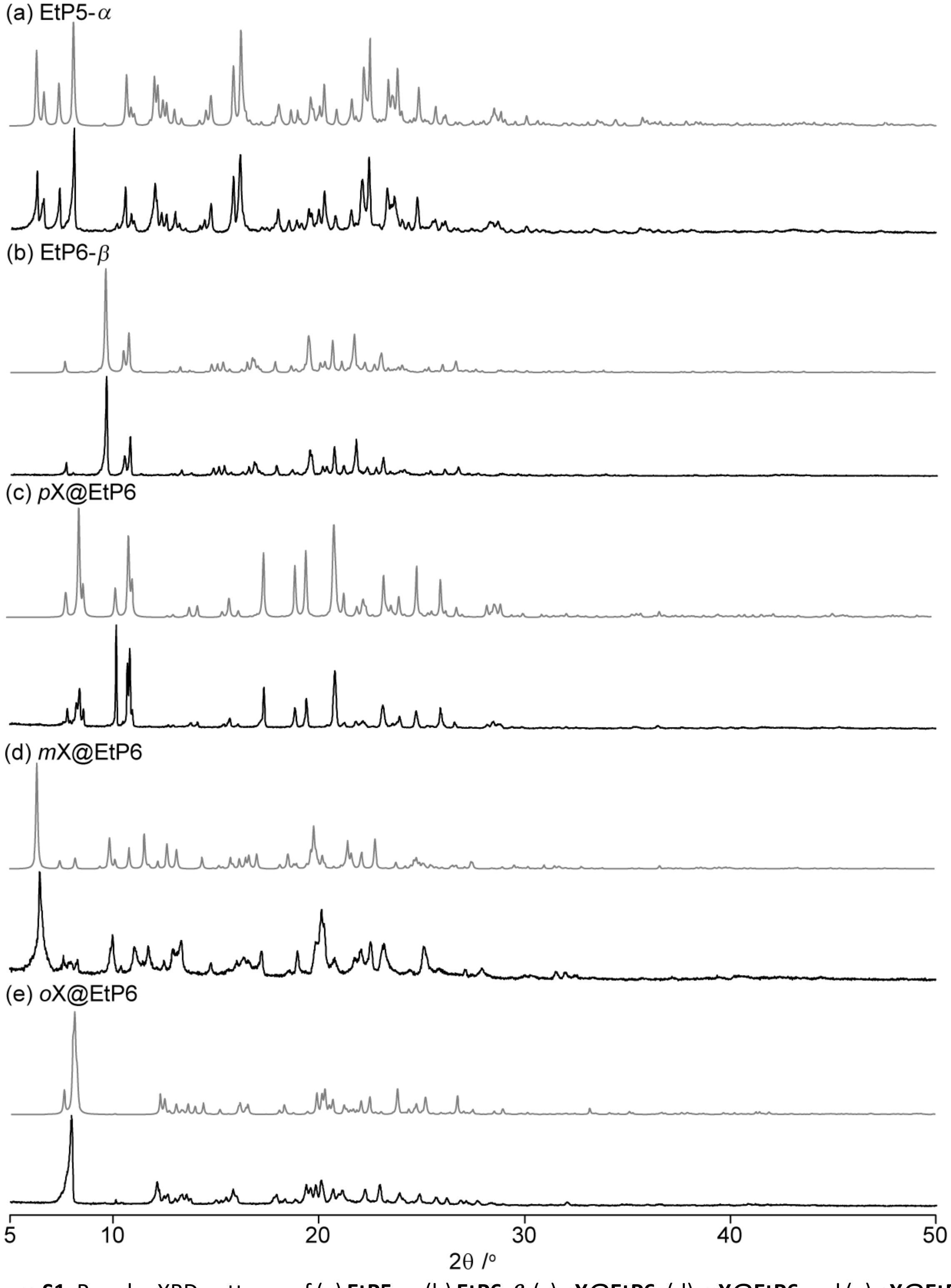

Figure S1. Powder XRD patterns of (a) **EtP5-*α***, (b) **EtP6-*β***, (c) ***p*X@EtP6**, (d) ***m*X@EtP6** and (e) ***o*X@EtP6**. Experimental and simulated data from single crystal structure data[2] are given in black and grey lines, respectively. Data were collected in transmission mode on samples held on thin Mylar film in aluminium well plates on a Panalytical X'Pert PRO MPD equipped with a high throughput screening XYZ stage, X-ray focusing mirror, and PIXcel detector, using Ni-filtered Cu Kα radiation of wavelength 1.5406 Å. All PXRD patterns are consistent with those previously published in the literature.[2]

S3. Differential Scanning Calorimetry (DSC) data

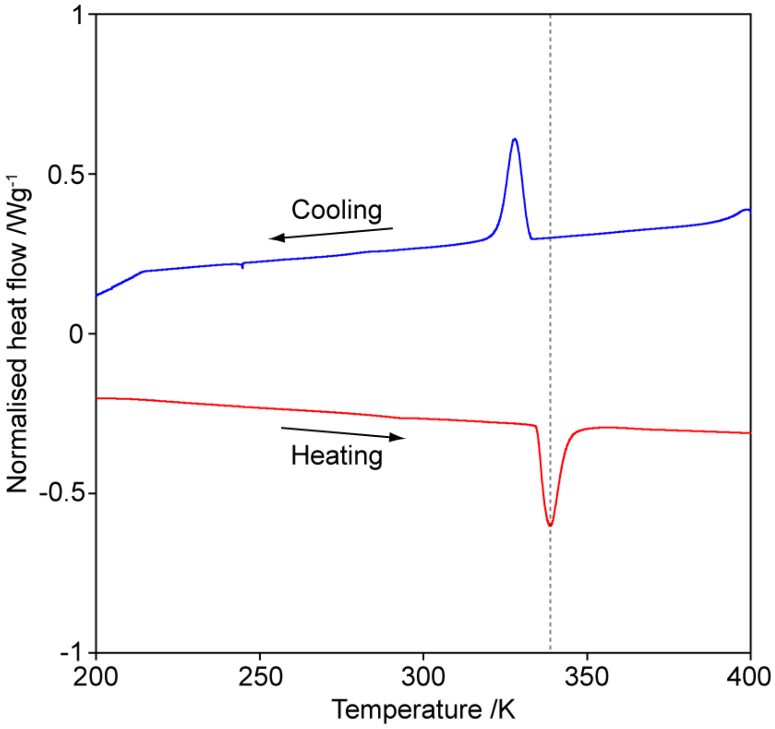

Figure S2. DSC thermogram of **EtP6-*β*** showing a phase transition from triclinic $P\bar{1}$ to a metastable, triclinic $P\bar{1}$ state with higher symmetry at 339 K (dashed line).[2] Data were collected using a TA Instruments Discovery DSC with the following heat treatment: equilibrate to 298 K, then ramp to 303 K at 10 K per minute, then cooled back to 188 K and ramped again to 303 K.

Figure S2 above shows a phase change of **EtP6-*β*** when being heated at 339 K. This confirms the phase change observed in the NMR data at 353 K.

S4. ThermoGravimetric Analysis (TGA) data

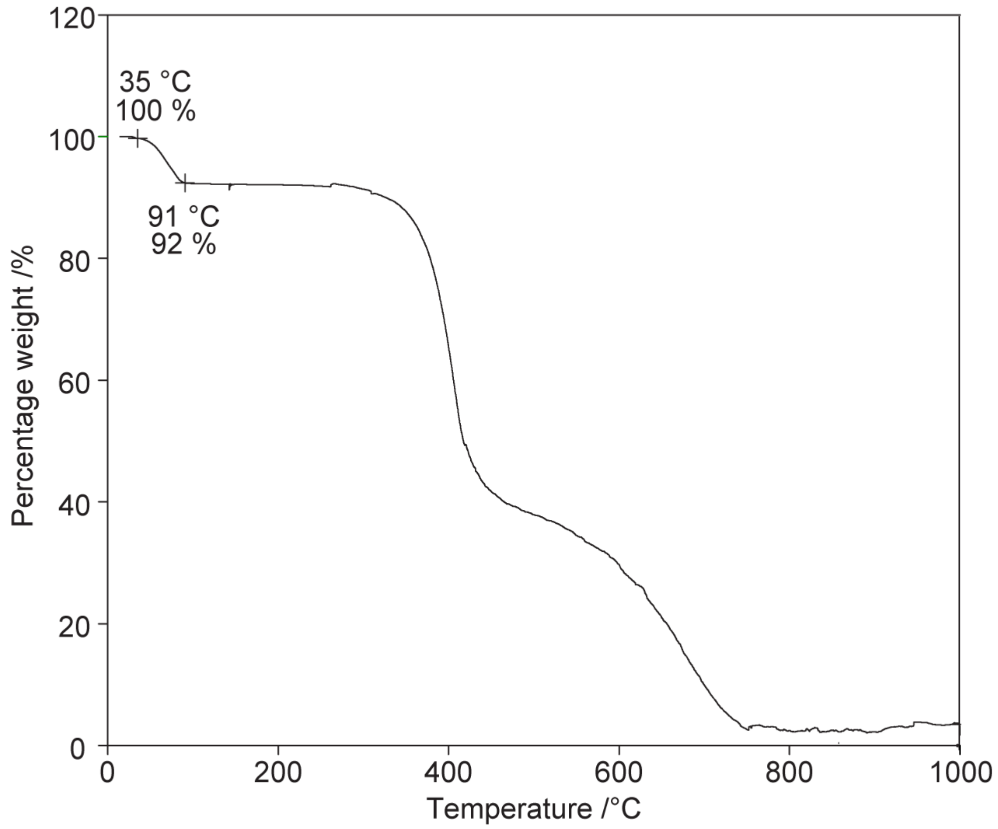

Figure S3. TGA data of ***m*X@EtP6**. The data showed a 8% weight loss that is consistent with the loss of one mole of *meta*-xylene between 35 and 91 °C.[2] Data were collected using a TA instrument Q5000IR analyzer with an automated vertical overhead thermobalance. ***m*X@EtP6** was heated at a rate of 10 °C/min, under a dry nitrogen gas flow.

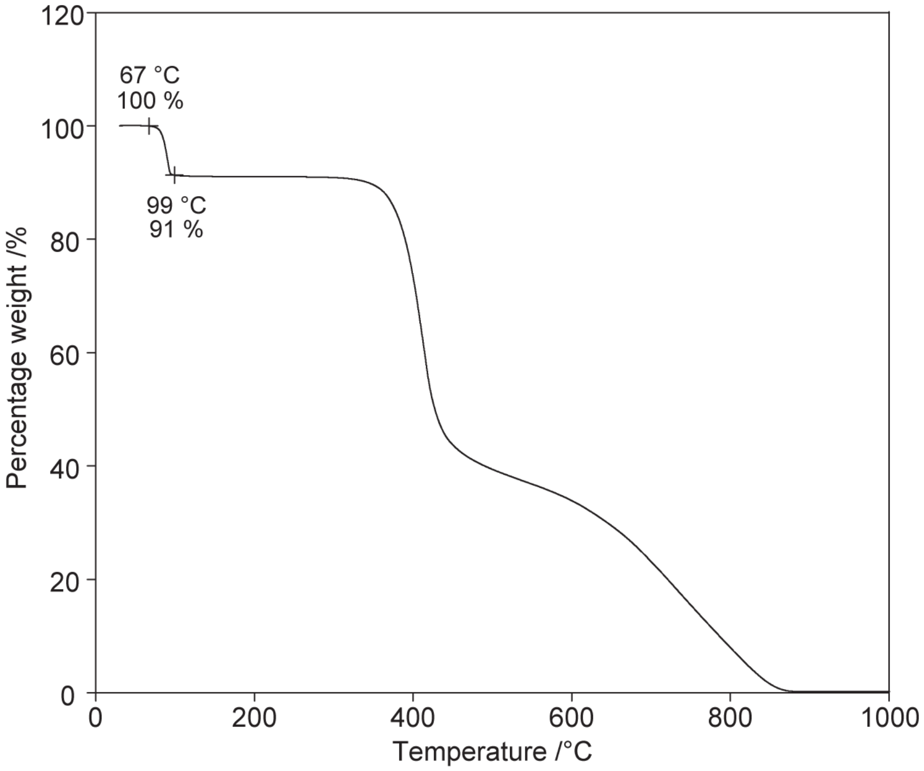

Figure S4. TGA data of ***o*X@EtP6**. The data showed a 9% weight loss that is consistent with the loss of one mole of *ortho*-xylene between 67 and 99 °C.[2] Data were collected using a TA instrument Q5000IR analyzer with an automated vertical overhead thermobalance. ***o*X@EtP6** was heated at a rate of 10 °C/min, under a dry nitrogen gas flow.

S5. NMR pulse programs

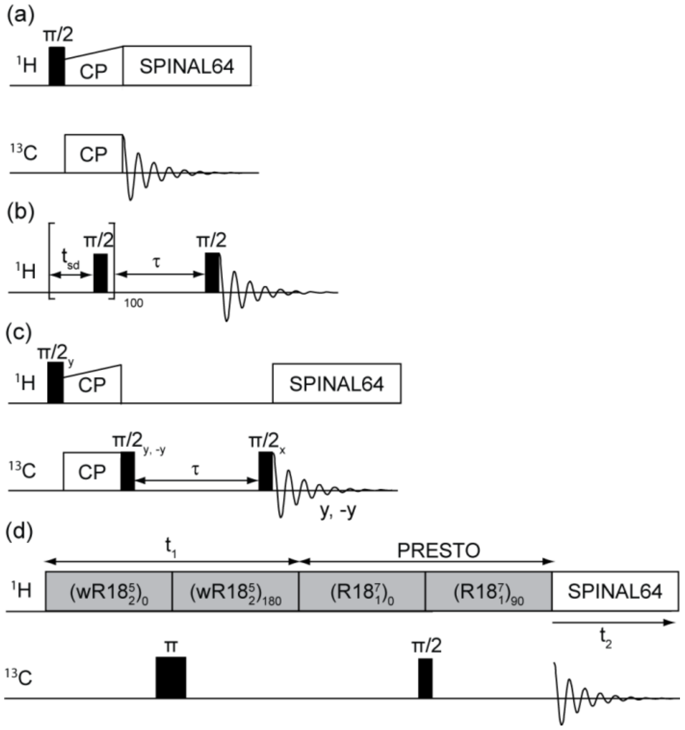

Figure S5. NMR pulse sequences used in this work. (a) Cross polarisation (CP) pulse sequence. (b) ^{1}H saturation recovery pulse sequence to measure ^{1}H T_1 times. t_{sd} is the saturation delay between $\pi/2$ pulses and was set to 10 ms at 9.4 T or 1 ms at 14.1 T. τ is the polarisation build-up time. (c) ^{13}C Torchia pulse sequence[25] to measure the ^{13}C T_1 times. τ is the polarisation recovery delay. (d) Proton Detected Local Field (PDLF) experiment using the windowed sequence (wPDLF)[3] that correlates the ^{13}C isotropic chemical shifts in ω_2 with the ^{13}C ^{1}H dipolar coupling spectra in ω_1. $\mathrm{wR}18^5_2$ recouples the heteronuclear dipolar interaction during the rotor synchronised evolution period t_1 (while suppressing ^{1}H ^{1}H homonuclear dipolar couplings) to enable observation of ^{13}C ^{1}H dipolar spectra. The $\mathrm{wR}18^7_1$ blocks also recouple the heteronuclear dipolar interaction to enable ^{1}H to ^{13}C polarisation transfer using the PRESTO sequence. SPINAL64 heteronuclear decoupling[4] was used during all ^{13}C acquisition.

S6. Scaling factor determination

Recoupling sequences to reintroduce heteronuclear dipolar coupling d_{CH} such as the R symmetry class[5–8] used in this work results in an effective dipolar coupling constant d_{CH}^{R} according to:

$$d_{CH}^{R} = \kappa_R d_{CH} \quad \textbf{(S1)}$$

where κ_R is the scaling factor of the recoupling sequences. Whilst this can often be determined numerically,[5] this is beyond the scope of this work for the $wR18_2^5$ block used and we have instead used a robust experimental method to determine κ_R from a $wR18_2^5$ PDLF experiment (**Figure S5(d)**) on model D-alanine as a reference. The corresponding ^{13}C CP NMR, 2D PDLF and site specific ^{13}C ^{1}H dipolar spectra are shown in **Figure S6** from which d_{CH}^{R} could be determined from the outer singularities (see Experimental Section) of the Cα carbon (d_{CH}^{R} = -7.55 kHz, **Table S1**). Comparison with the d_{CH} value (-23.14 kHz) obtained from **equation 1** in the main text and the α–carbon proton bond length (1.093 Å) determined from neutron diffraction[9] affords:

$$\kappa_R = \frac{d_{CH}^{R}}{d_{CH}} = \frac{-7550}{-23140} = 0.326 \quad \textbf{(S2)}$$

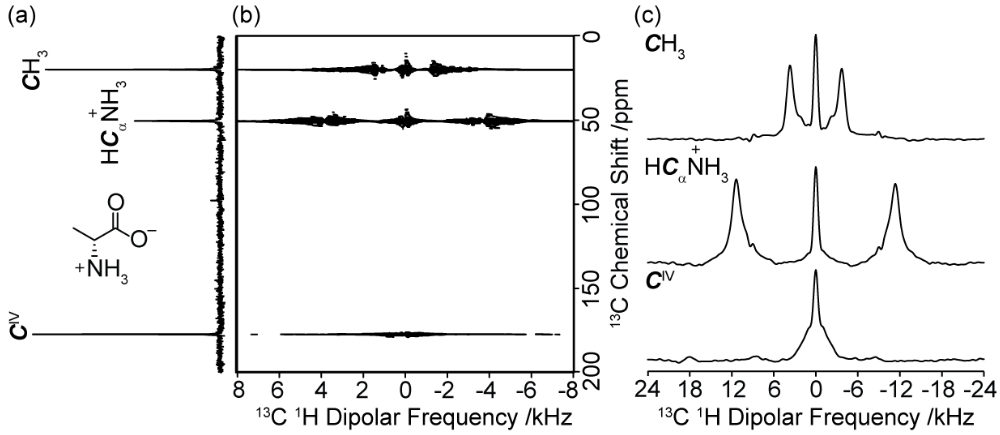

Figure S6. (a) ^{13}C CP MAS spectrum and chemical structure, (b) PDLF spectrum and (c) site-specific ^{13}C ^{1}H dipolar spectra of D-alanine. Spectral assignments are given in the figure. Data obtained at 298 K and 9.4 T. d_{CH}^{R} is measured using the outer singularities of the dipolar coupling spectra. Vertical light grey lines in the dipolar coupling spectra indicate the static limit dipolar coupling constants d_{CH}.

Table S1. ^{13}C NMR spectrum assignments, ^{13}C isotropic chemical shifts and the motional averaged dipolar coupling obtained from the 2D PDLF spectrum of D-alanine shown in **Figure S6** above.

Assignment	^{13}C δ_{iso}/ppm	d_{CH}^{R}/kHz	d_{CH}/kHz
CH3	20.7	-2.45	-7.50
H***C***αNH3	51.2	-7.55	-23.10
CIV	178.1	-0.65	-1.99

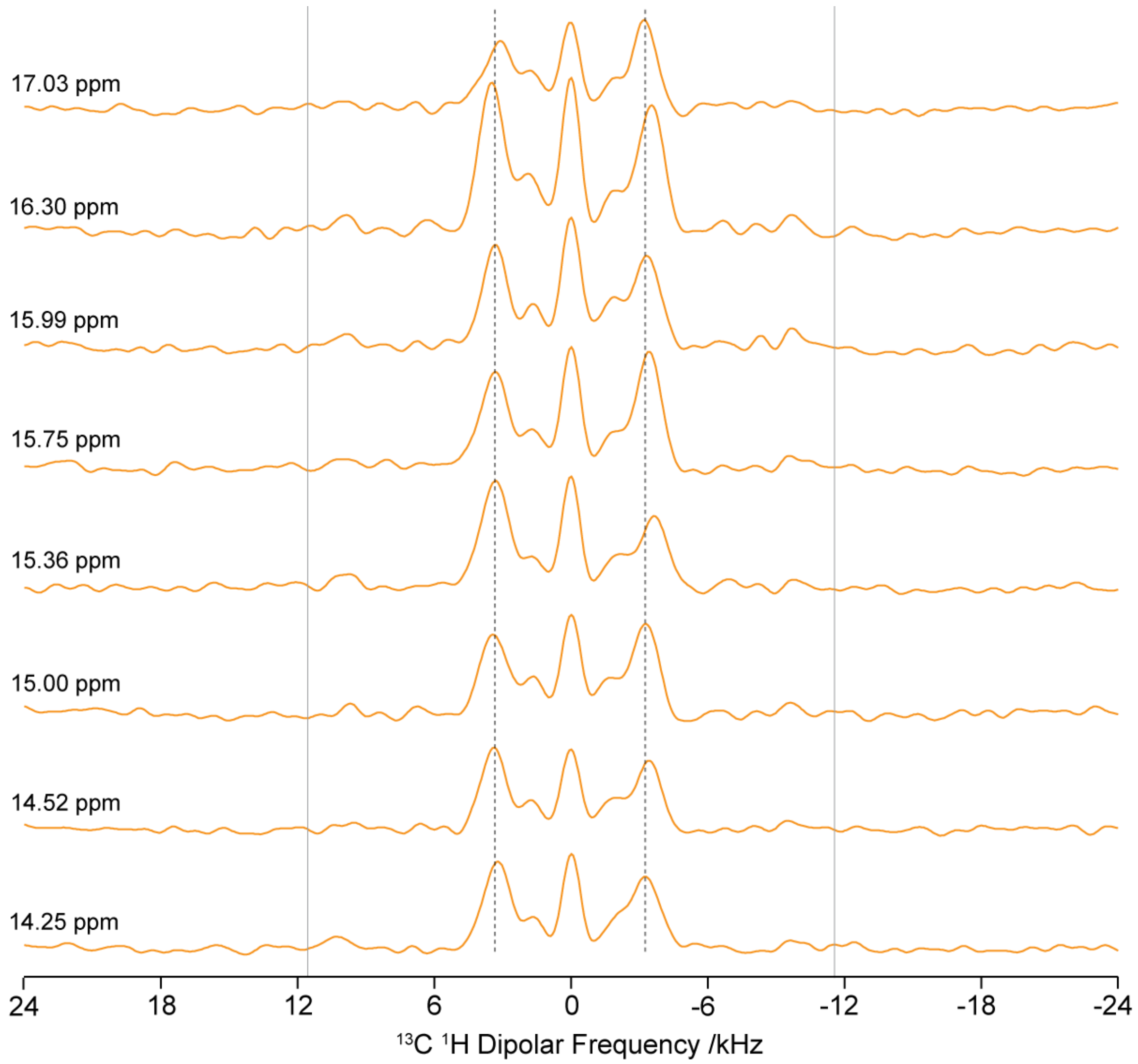

Figure S7. Comparison of the ^{13}C ^{1}H dipolar spectra for each resolved ***C***H_3 resonance in **EtP6-*β*.**[2] Data were obtained at 298 K and a magnetic field of 9.4 T. The corresponding ^{13}C isotropic chemical shift (δ_{iso}) is given next to each dipolar spectrum. Dashed lines indicate the average $<d_{CH}>$ = -7.2 kHz obtained from this data which also illustrate very small change between each dipolar spectrum ($\Delta<d_{CH}>$ = 1.0 kHz); hence, in this dataset $<d_{CH}>$ = -7.1 ± 0.5 kHz. Vertical light grey lines in the spectra indicate the static limit dipolar coupling constants d_{CH}.

S7. ^{13}C NMR spectra assignments

In ***p*X@EtP6**, each different carbon subgroups resonances in the EtP6 host consist of multiple peaks (**Figure 2(c)**, **Table S4**) which number and integration are in agreement with the number of carbons in asymmetric unit cell (*e.g.*, 6 for ***C***H_2, 12 for O***C***^{IV} etc),[2] while the *para*-xylene resonances can be mostly assigned based on their δ_{iso}. There are spectral overlaps in the 124 - 136 ppm region between the quaternary CH_2***C***^{IV} resonances of EtP6 host and the protonated ***C***Hs of *para*-xylene and the spectral assignment was performed with ^{13}C-edited CP MAS spectra. Two distinguishable ***C***H_3s, ***C***Hs and quaternary carbons resonances are observed for *para*-xylene in ***p*X@EtP6** which is in agreement with the asymmetric unit cell of this phase. Likewise, the ^{13}C NMR spectrum of ***m*X@EtP6** (**Figure S9** and **Table S5**) reveals one *meta*-xylene molecule per host in the asymmetric unit cell. We point out that the ^{13}C resonances for *meta*-xylene in ***m*X@EtP6** and *meta*-xylene in solution[10] are very close (less than 1.6 ppm), which strongly supports a limited effect on the chemical shielding of *meta*-xylene from EtP6 resulting from size exclusion (**Figure 1(d)**).

The ^{13}C NMR spectrum of ***o*X@EtP6** (**Figure 2(e)**) allows the observation of all different types of carbon in EtP6. However, the spectrum is more poorly resolved than those of ***p*X@EtP6** and ***m*X@EtP6**, likely due to the lower crystallinity of this material (PXRD data in **Figure S1**), we only provide a tentative assignment for overlapping NMR resonances between *ortho*-xylene and the EtP6 host (**Table S6**). Notably, the short contact time ^{13}C CP spectrum (**Figure S10**) only enables clear identification of the CHs originating from the *ortho*-xylene while its ***C***H_3 resonance is not directly attributable. This resonance is likely shifted to a lower frequency than in ***m*X@EtP6** and ***p*X@EtP6** due to the increased shielding effect of the *ortho*-xylene methyl groups located in the centre of the cavity (**Figure 1(e)**) and is therefore expected to overlap with the CH_3 resonances of the ethyl group around 16 ppm.

The room temperature PDLF data on the three xylene adducts revealed observable dipolar coupling for the methyl groups and ***C***Hs of the xylenes as expected (**Figures S20-S22** and Section S9 below), which combined with ^{13}C-edited CP MAS spectra (**Figures S8-S10**), were used to aid spectral assignments, in particular by differentiating these ***C***Hs from the CH_2***C***^{IV} resonances of EtP6 that appear in the same spectral region (**Figures 2** and **S16-S18**).

Table S2. ^{13}C NMR assignments for **EtP5-α** (**Figure 2(a)**) with isotropic chemical shifts δ_{iso} from deconvoluted data previously published,[2] spin lattice relaxation times T_1 obtained, motional averaged dipolar coupling constants $<d_{CH}>$ and order parameters $<S_{CH}>$. Data obtained at room temperature and 9.4 T.

Assignment	^{13}C δ_{iso}/ppm	T_1/s	$<d_{CH}>$/kHz	$<S_{CH}>$
CH$_3$	14.42<br>15.13<br>15.71<br>16.22<br>16.83	1.7 ± 0.1	-7.2 ± 0.5	0.31 ± 0.03
CH$_2$	28.01<br>28.67<br>29.63<br>35.07<br>37.53	166 ± 49	-23.3 ± 0.8	1.01 ± 0.03
O***C***H$_2$	61.16<br>62.50<br>63.22<br>64.15<br>64.45<br>64.73<br>67.42	44 ± 5	-18.4 ± 0.7	0.79 ± 0.03
CH	111.78<br>112.49<br>112.76<br>113.00<br>113.35<br>114.29<br>115.97<br>117.32<br>123.06	246 ± 27	-23.8 ± 0.8	1.03 ± 0.03
CH$_2$***C***^{IV}	127.12<br>127.39<br>127.75<br>128.49<br>129.44<br>130.22<br>130.65<br>135.36	262 ± 56	N/A[a]	N/A
O***C***^{IV}	146.51<br>148.30<br>149.33<br>150.12<br>150.31<br>151.16<br>151.53<br>152.56<br>154.12	217 ± 33	N/A	N/A

[a] Not Applicable.

Table S3. ^{13}C NMR assignments for **EtP6-*β*** (**Figure 2(b)**) with isotropic chemical shifts δ_{iso} from deconvoluted data previously published,[2] spin lattice relaxation times T_1 obtained *via* area integration, motional averaged dipolar coupling constants $\langle d_{CH} \rangle$ and order parameters $\langle S_{CH} \rangle$. Data obtained at room temperature and 9.4 T.

Assignment	^{13}C δ_{iso}/ppm	T_1/s	$\langle d_{CH} \rangle$/kHz	$\langle S_{CH} \rangle$
CH$_3$	14.25<br>14.52<br>15.00<br>15.36<br>15.75<br>15.99<br>16.30<br>17.03	2.4 ± 0.1	-7.2 ± 0.5	0.31 ± 0.03
CH$_2$	27.83<br>28.58<br>29.43<br>29.88<br>33.27<br>34.09	182 ± 34	-22.4 ± 0.8	0.97 ± 0.03
O***C***H$_2$	62.15<br>62.80<br>63.47<br>63.75<br>64.78<br>65.30<br>65.48<br>66.15	25 ± 2	-18.1 ± 0.7	0.78 ± 0.03
CH	111.68<br>112.01<br>112.98<br>113.95<br>115.20<br>116.11<br>116.55<br>117.28	262 ± 27	-23.9 ± 0.8	1.03 ± 0.03
CH$_2$***C***^{IV}	123.86<br>124.30<br>125.83<br>126.72<br>127.77<br>128.45<br>129.05<br>131.38<br>131.76	236 ± 26	N/A[a]	N/A
O***C***^{IV}	150.08<br>151.13<br>150.40<br>150.67<br>151.38<br>153.00<br>153.92	227 ± 14	N/A	N/A

[a] Not Applicable.

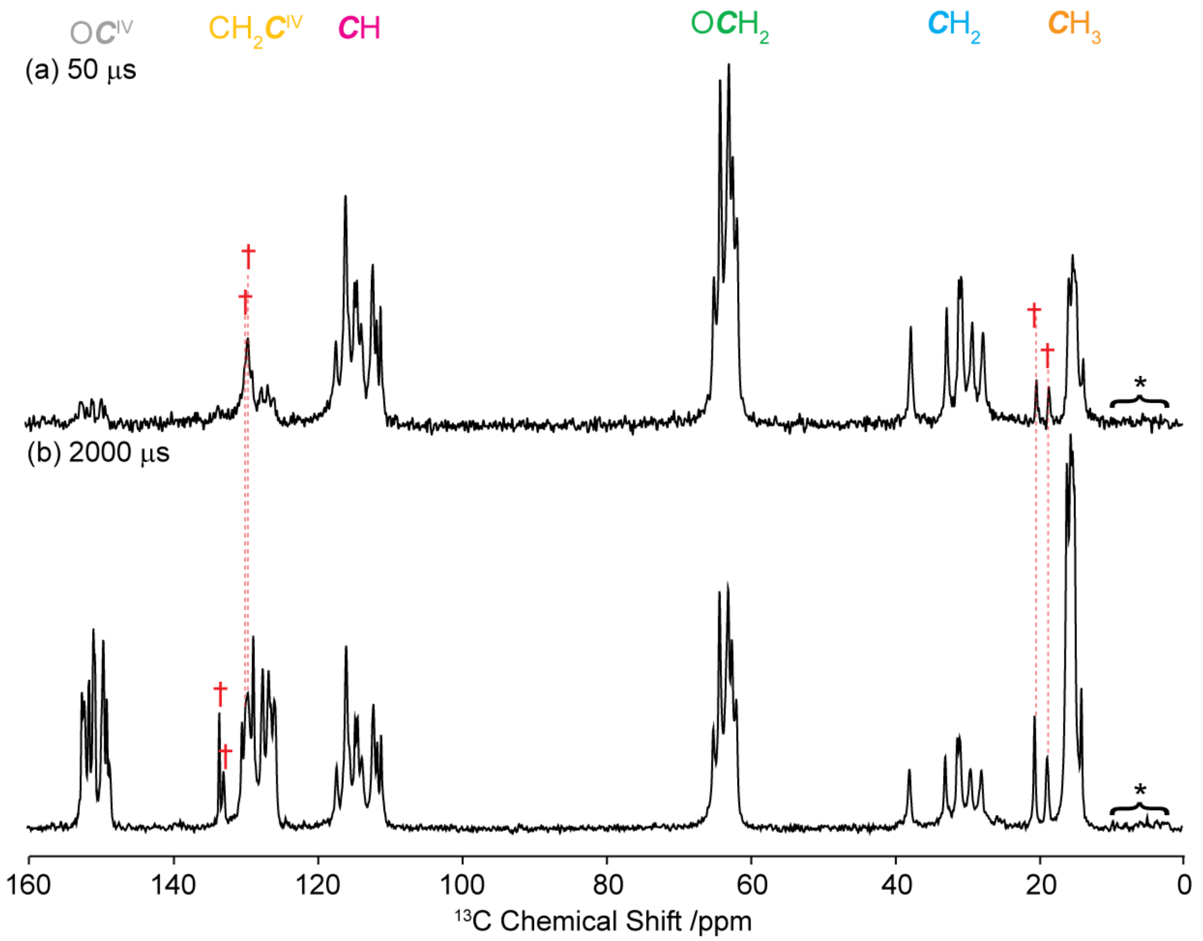

Figure S8. ^{13}C edited experiments of ***pX@EtP6*** obtained by comparing the ^{13}C CP MAS NMR spectra acquired with contact times of (a) 50 μs and (b) 2000 μs. Data were obtained at a magnetic field of 9.4 T. Spectral assignments are given in the figure (see **Figure 1**). The red daggers (†) (also shown in red in the spectral deconvolution in **Figure S11**) denote signals arising from the *para*-xylene guest. The ***C***H resonances of *para*-xylene are apparent in the spectrum obtained at a short contact time of 50 μs where the signal intensities of the quaternary carbons are largely reduced. Spinning sidebands are marked with asterisks (*).

Table S4. ^{13}C NMR assignments for ***pX@EtP6*** (**Figure 2(c)**) with isotropic chemical shifts δ_{iso} from spectral deconvolution in **Figure S11**, spin lattice relaxation times T_1 obtained *via* area integration, motional averaged dipolar coupling constants $\langle d_{CH} \rangle$ and order parameters $\langle S_{CH} \rangle$. Data obtained at room temperature and 9.4 T. Red daggers (†) denote signals arising from *para*-xylene guest.

Assignment	^{13}C δ_{iso}/ppm	T_1/s	$\langle d_{CH} \rangle$/kHz	$\langle S_{CH} \rangle$
CH$_3$	14.0<br>14.4<br>14.9<br>15.2<br>15.5<br>16.0<br>18.7 (†)<br>20.5 (†)	15.9 ± 0.1	-6.9 ± 0.5	0.30 ± 0.03
CH$_2$	27.9<br>29.4<br>30.8<br>31.2<br>32.8<br>37.8	86 ± 32	-21.6 ± 0.8	0.94 ± 0.03
O***C***H$_2$	61.8<br>62.4<br>62.9<br>63.3<br>6 3.9<br>64.1<br>64.8<br>65.0	39 ± 3	-18.4 ± 0.7	0.81 ± 0.03
CH	111.0<br>111.5<br>112.1<br>113.6<br>114.2<br>114.6<br>115.4<br>115.8<br>117.2	140 ± 10	-22.4 ± 0.8	0.94 ± 0.03
CH$_2$***C***^{IV}	125.8<br>126.3<br>126.6<br>127.4<br>127.6<br>128.7<br>129.4 (†)<br>129.8 (†)<br>130.3<br>132.8 (†)<br>133.4 (†)	140 ± 15	N/A[a]	N/A
O***C***^{IV}	148.6<br>149.0<br>149.5<br>149.7<br>150.6<br>150.9<br>151.5<br>152.1<br>152.5	127 ± 11	N/A	N/A

[a] Not Applicable.

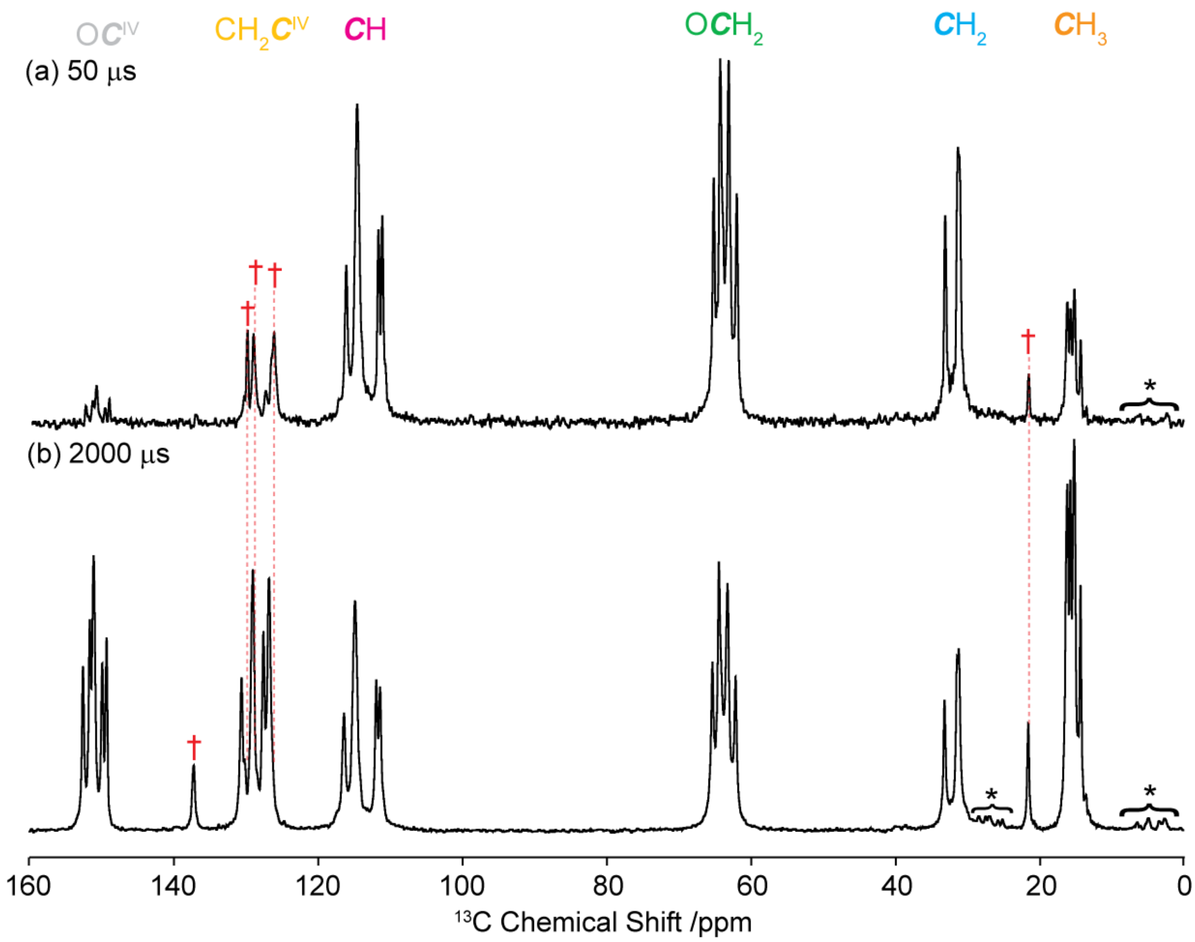

Figure S9. ^{13}C edited experiments of ***mX@EtP6*** obtained by comparing the ^{13}C CP MAS NMR spectra acquired with contact times of (a) 50 µs and (b) 2000 µs. Data were obtained at a magnetic field of 9.4 T. Spectral assignments are given in the figure (see **Figure 1**). The red daggers (†) (also shown in red in the spectral deconvolution in **Figure S12**) denote signals arising from the *meta*-xylene guest. The ***C***H resonances of *meta*-xylene are apparent in the spectrum obtained at a short contact time of 50 µs where the signal intensities of the quaternary carbons are largely reduced. Spinning sidebands are marked with asterisks (*).

Table S5. ^{13}C NMR assignments for ***mX@EtP6*** (**Figure 2(d)**) with isotropic chemical shifts δ_{iso} from spectral deconvolution in **Figure S12**, spin lattice relaxation times T_1 obtained *via* area integration, motional averaged dipolar coupling constants $<d_{CH}>$ and order parameters $<S_{CH}>$. Data obtained at room temperature and 9.4 T. Red daggers (†) denote signals arising from *meta*-xylene guest.

Assignment	^{13}C δ_{iso}/ppm	T_1/s	$<d_{CH}>$/kHz	$<S_{CH}>$
CH_3	14.4<br>15.2<br>15.3<br>15.8<br>16.2<br>16.4<br>21.6 (†)	2.8 ± 0.1	-6.9 ± 0.5	0.30 ± 0.03
CH_2	31.2<br>31.5<br>33.2	168 ± 17	-22.2 ± 0.8	0.97 ± 0.03
O***C***H_2	62.2<br>63.3<br>64.2<br>64.5<br>65.4	45 ± 3	-19.2 ± 0.7	0.84 ± 0.03
CH	111.4<br>111.9<br>114.9<br>116.4	158 ± 11	-23.5 ± 0.8	0.99 ± 0.03
CH_2***C***^{IV}	126.6 (†)<br>126.8<br>127.6<br>128.4 (†)<br>129.0<br>130.1 (†)<br>130.6<br>137.2 (†)	136 ± 9	N/A[a]	N/A
O***C***^{IV}	149.3<br>149.9<br>151.1<br>151.6<br>152.5	189 ± 11	N/A	N/A

[a] Not Applicable.

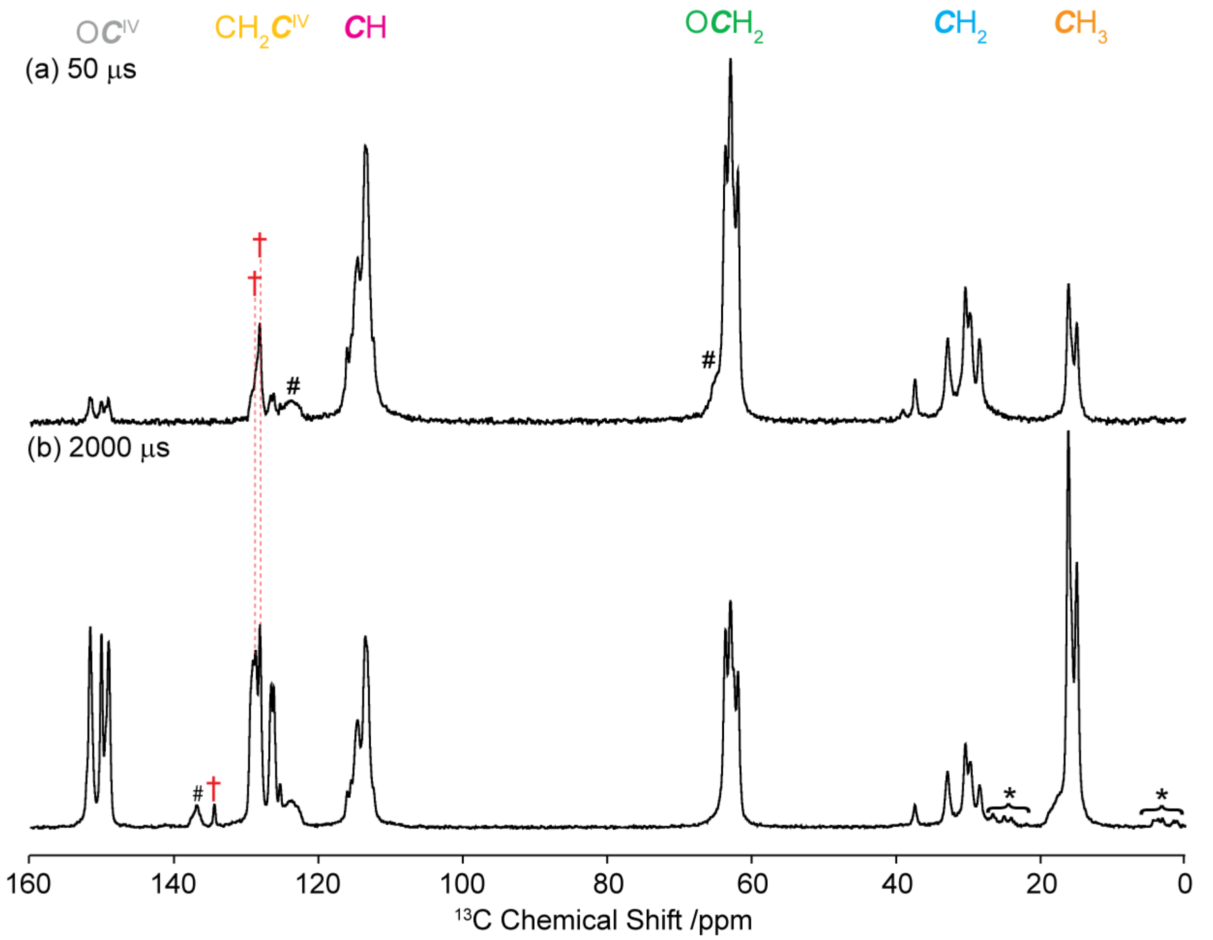

Figure S10. ^{13}C edited experiments of ***o*****X@EtP6** obtained by comparing the ^{13}C CP MAS NMR spectra acquired with contact times of (a) 50 μs and (b) 2000 μs. Data were obtained at a magnetic field of 9.4 T. Spectral assignments are given in the figure (see **Figure 1**). The red daggers (†) (also shown in red in the spectral deconvolution (**Figure S13**) denote signals arising from the *ortho*-xylene guest. The ***C***H resonances of *ortho*-xylene are apparent in the spectrum obtained at a short contact time of 50 μs where the signal intensities of the quaternary carbons are largely reduced. Spinning sidebands and signals arising from unknown minor phase(s) are marked with asterisks (*) and dashes (#), respectively.

Table S6. ^{13}C NMR assignments for ***oX@EtP6*** (**Figure 2(e)**) with isotropic chemical shifts δ_{iso} from spectral deconvolution shown in **Figure S13**, spin lattice relaxation times T_1 obtained *via* area integration, motional averaged dipolar coupling constants $\langle d_{CH} \rangle$ and order parameters $\langle S_{CH} \rangle$. Data obtained at room temperature and 9.4 T. Red daggers (†) denote signals arising from the aromatic carbons in *ortho*-xylene guest (the CH_3 is not resolved) and (#) denote amorphous signals. Overlapping resonances between the guest and the host in the in the 15-17 ppm and 123-137 ppm region of the ^{13}C CP MAS NMR spectrum prevents spectral assignment of the ***C***H_3 and only allow tentative assignment of the CH_2***C***^{IV} carbons.

Assignment	^{13}C δ_{iso}/ppm	T_1/s	$\langle d_{CH} \rangle$/kHz	$\langle S_{CH} \rangle$
CH_3	15.1<br>15.9<br>16.3	2.0 ± 0.1	-7.1 ± 0.5	0.31 ± 0.03
CH_2	28.6<br>29.8<br>30.6<br>33.0<br>37.6	65 ± 7	-23.8 ± 0.8	1.03 ± 0.03
O***C***H_2	62.1<br>62.7<br>63.1<br>63.8<br>65.4 (#)	26 ± 2	-18.3 ± 0.7	0.81 ± 0.03
CH	111.4<br>111.9<br>114.9<br>116.4	89 ± 4	-23.8 ± 0.8	1.00 ± 0.03
CH_2***C***^{IV}	124.0 (#)<br>125.4<br>126.3<br>126.7<br>128.2 (†)<br>128.8 (†)<br>129.2<br>129.5<br>134.6 (†)<br>137.0 (#)	52 ± 5	N/A[a]	N/A
O***C***^{IV}	149.2<br>149.6<br>150.2<br>151.8<br>151.5<br>152.1	88 ± 4	N/A	N/A

[a] Not Applicable.

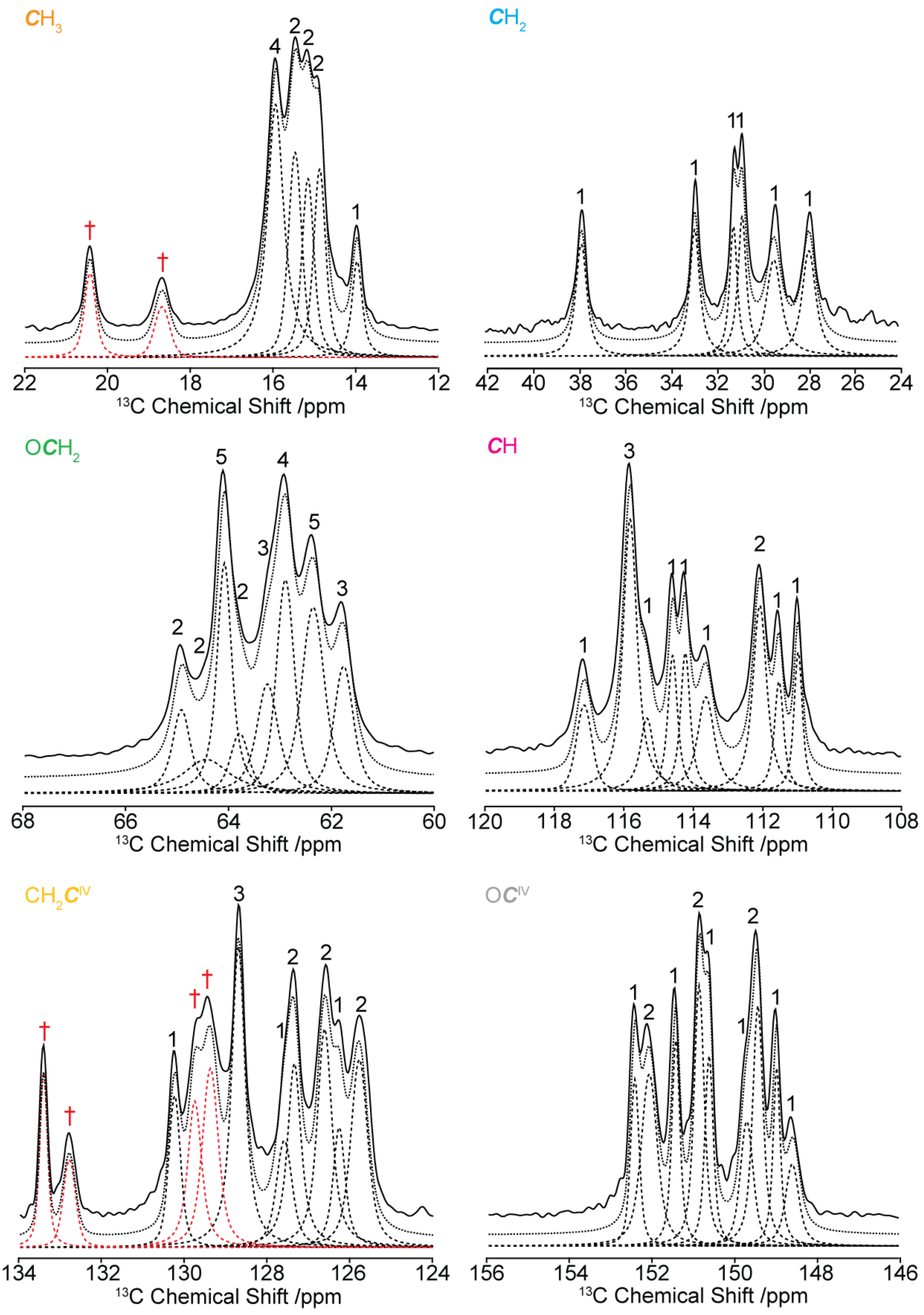

Figure S11. Experimental ^{13}C CP MAS NMR spectrum of ***pX@EtP6*** (full line) with the total fit and spectrum deconvolutions shown with dotted and dashed lines, respectively. Integration values for each carbon subgroup are given. Data obtained at 298 K and 9.4 T. Each panel shows chemically distinct carbons environments of the EtP6 host. Spectral assignments are given in the figure (see **Figure 1**) and are obtained from known isotropic chemical shifts, ^{13}C-edited experiments and 2D PDLF data (see manuscript). Note that the low resolution of some of the resonances results in uncertainty in the estimated integration. Red daggers (†) denote signals arising from the *para*-xylene guest.

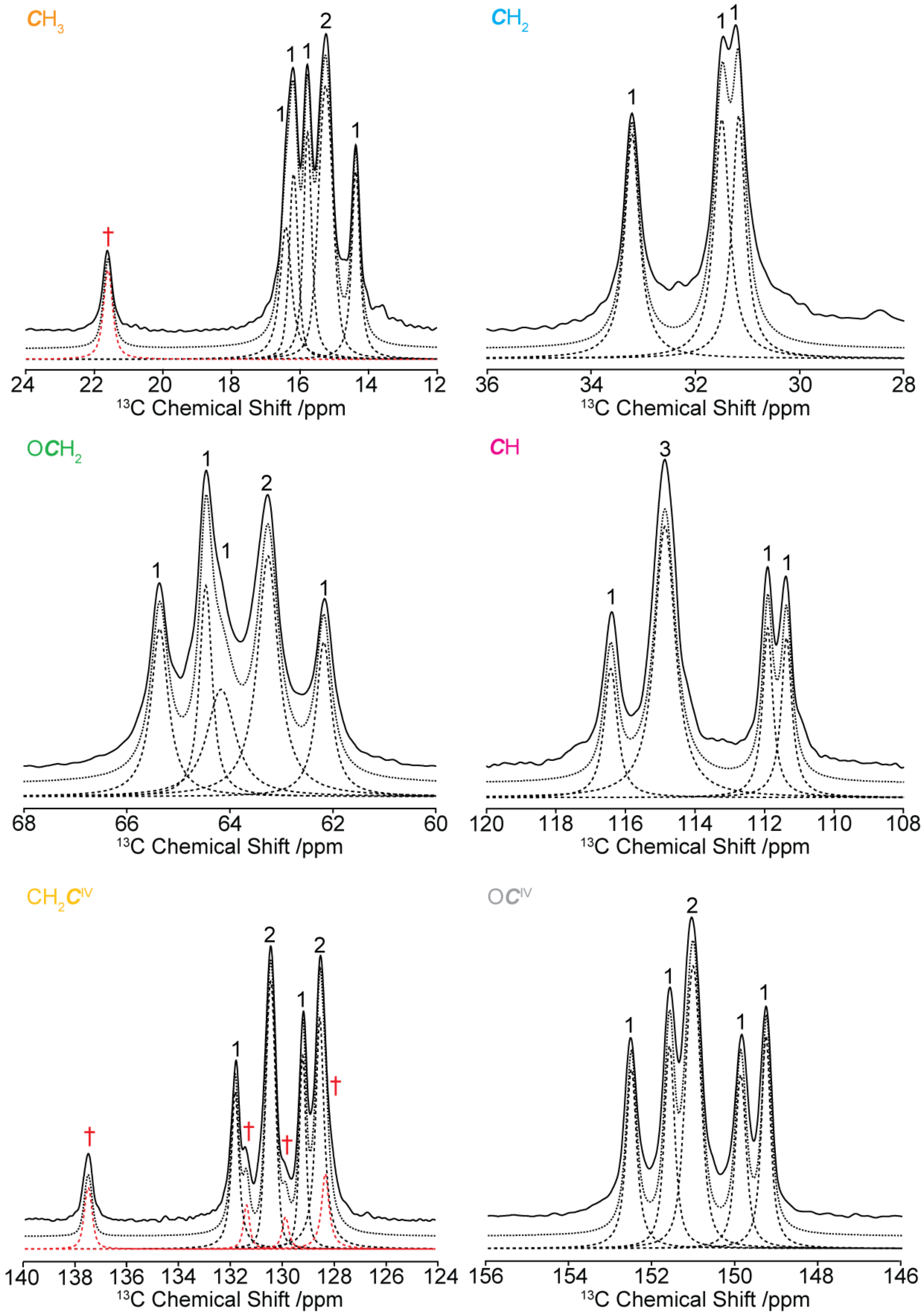

Figure S12. Experimental ^{13}C CP MAS NMR spectrum of ***mX@EtP6*** (full line) with the total fit and spectrum deconvolutions shown with dotted and dashed lines, respectively. Integration values for each carbon subgroup are given. Data obtained at 298 K and 9.4 T. Each panel shows chemically distinct carbons environments of the EtP6 host. Spectral assignments are given in the figure (see **Figure 1**) and are obtained from known isotropic chemical shifts, ^{13}C-edited experiments and 2D PDLF data (see manuscript). Red daggers (†) denote signals arising from the *meta*-xylene guest.

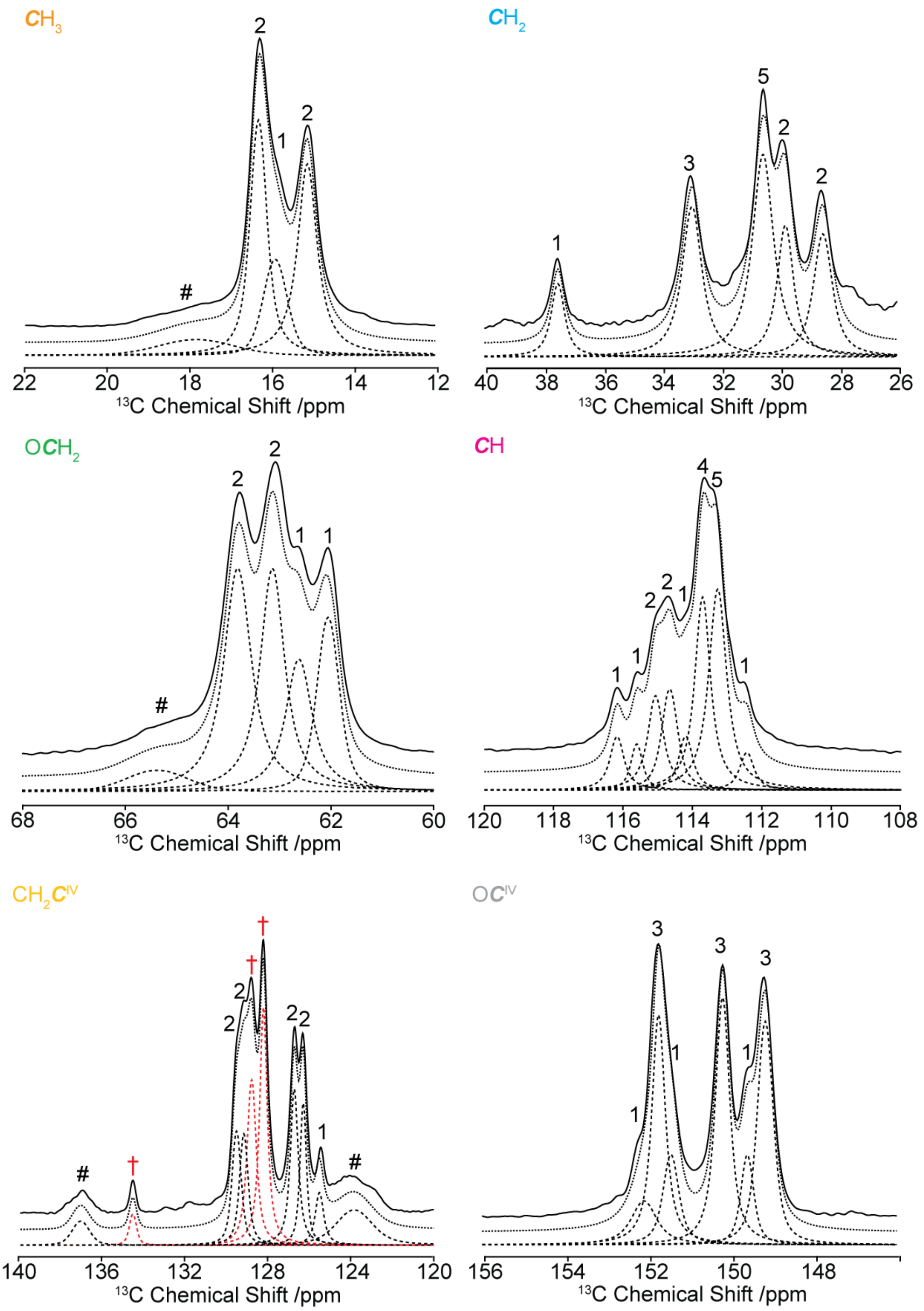

Figure S13. Experimental ^{13}C CP MAS NMR spectrum of ***o*X@EtP6** (full line) with the total fit and spectrum deconvolutions shown with dotted and dashed lines, respectively. Integration values for each carbon subgroup are given. Data obtained at 298 K and 9.4 T. Each panel shows chemically distinct carbons environments of the EtP6 host. Spectral assignments are given in the figure (see **Figure 1**) and are obtained from known isotropic chemical shifts, ^{13}C-edited experiments and 2D PDLF data (see manuscript). Note that the low resolution of some of the resonances results in uncertainty in the estimated integration. The overlapping peaks in the ***C***H_3 region do not allow for the ***C***H_3 of the *ortho*-xylene guest to be accurately identified. Broad signals tentatively assigned to some unknown amorphous phase(s) are marked with dashes (#). Red daggers (†) denote signals arising from the *ortho*-xylene guest.

S8. Variable temperature ^{13}C NMR spectra

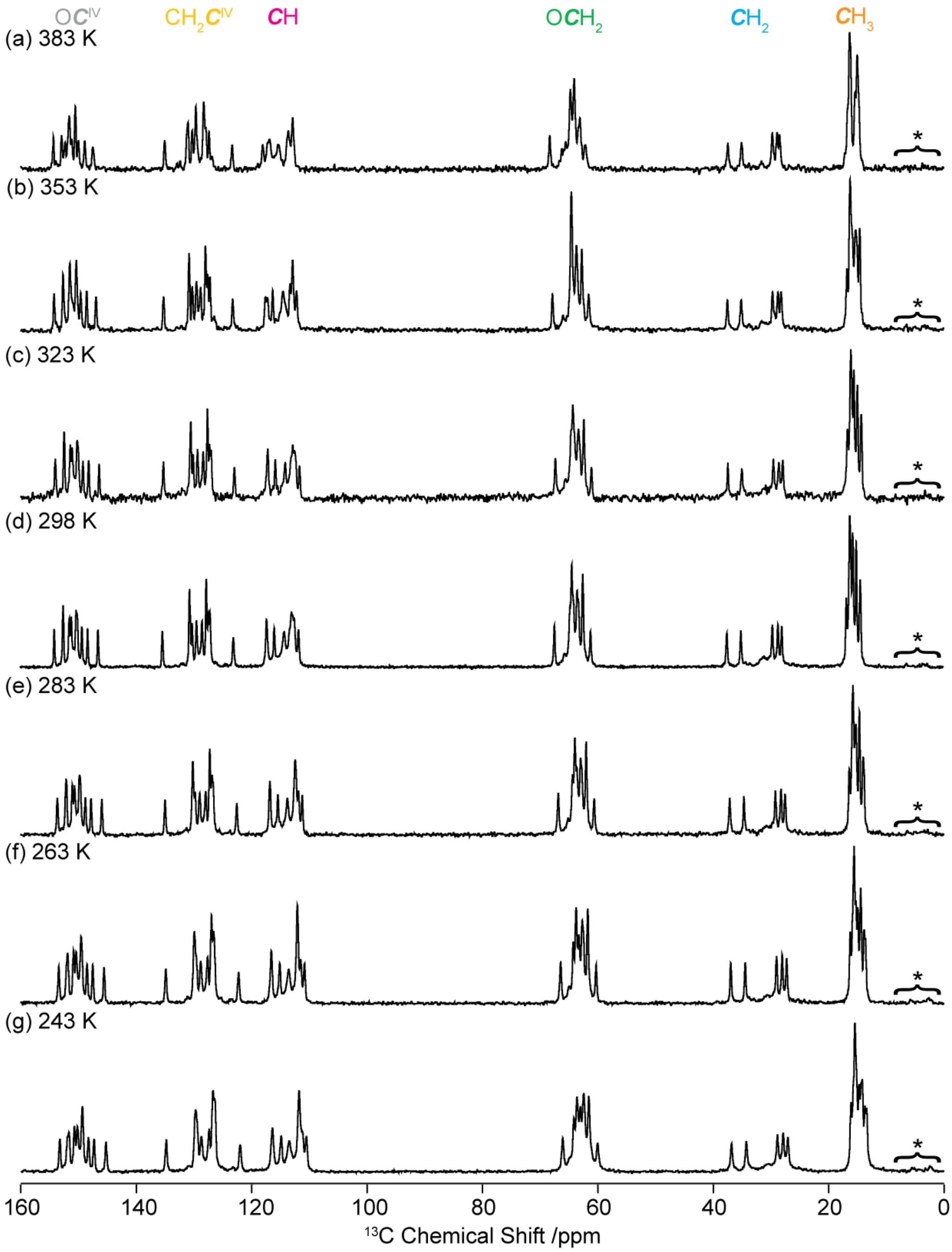

Figure S14. Variable temperature ^{13}C CP NMR spectra of guest-free **EtP5-α** collected at the temperatures given in the figure. Data was obtained at 9.4 T. Spectral assignments are given in the figure (see **Figure 1**) and correspond to those previously published.[1] Asterisks (*) denote spinning sidebands.

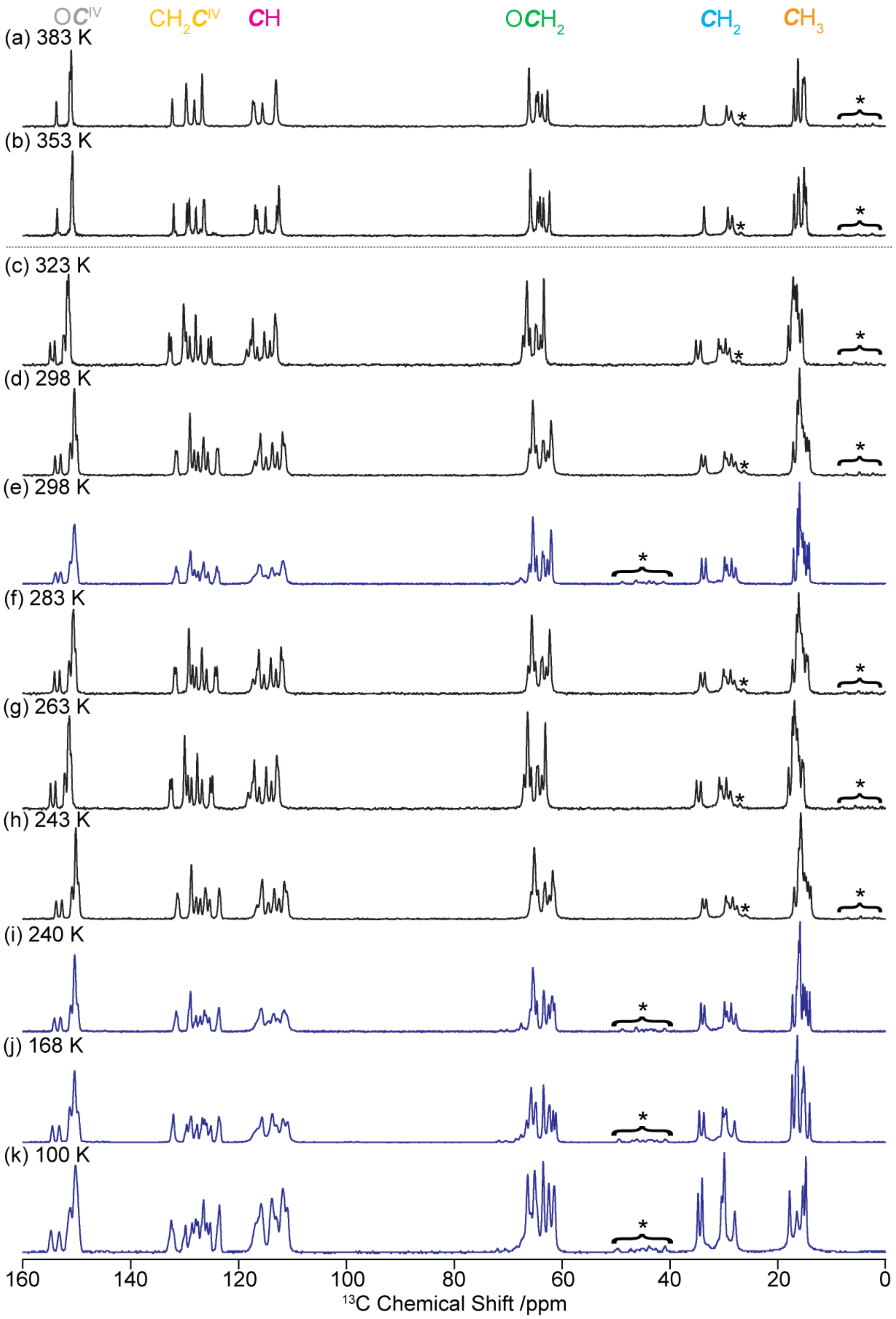

Figure S15. Variable temperature ^{13}C CP NMR spectra of guest-free **EtP6-β** collected at the temperatures given in the figure. Data shown in black and blue were obtained at 9.4 and 14.1 T, respectively. Spectral assignments are given in the figure (see **Figure 1**) and correspond to those previously published.[1] The dotted line indicates a polymorphic transition in **EtP6-β** (see DSC data in **Figure S2**). Asterisks (*) denote spinning sidebands.

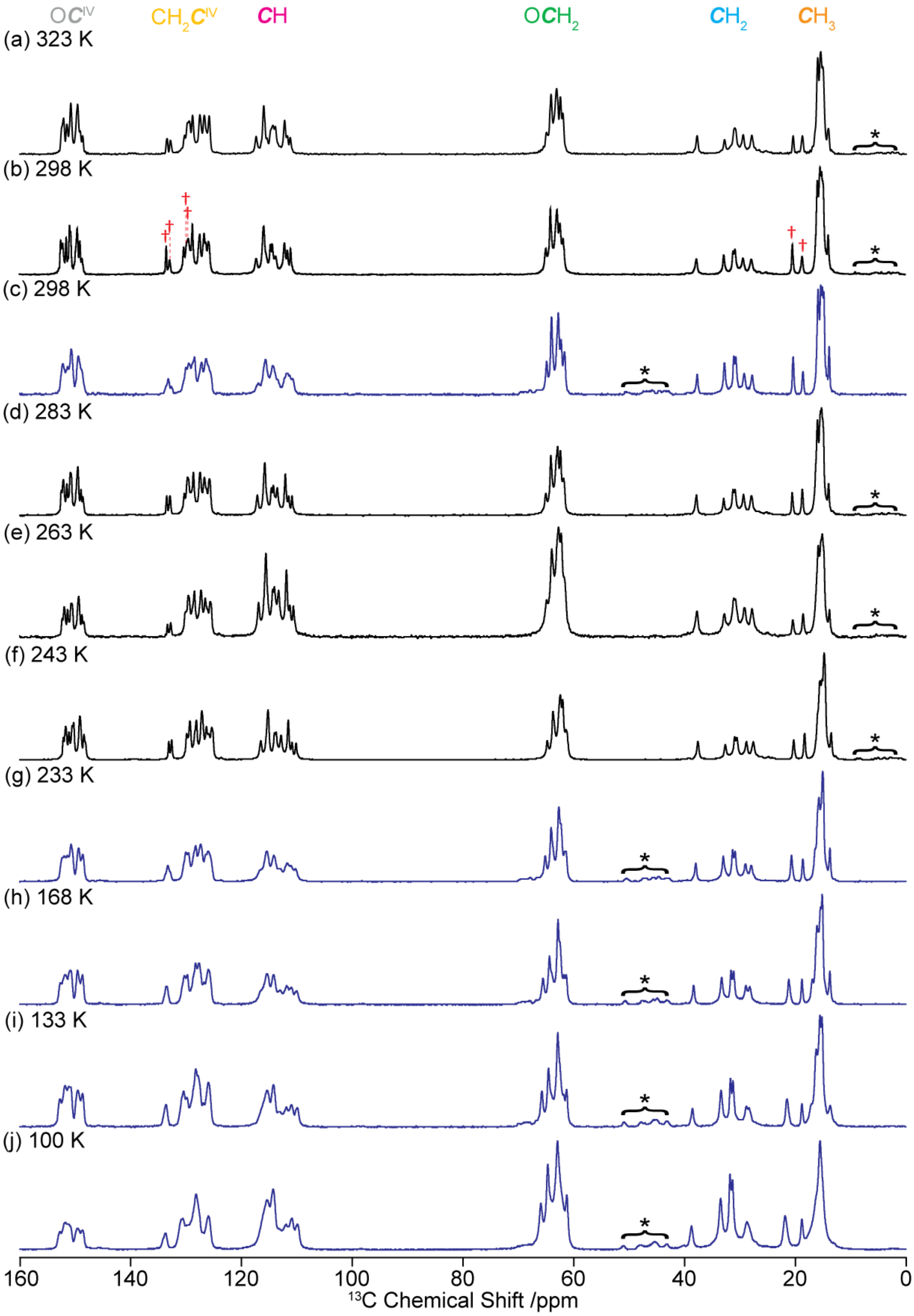

Figure S16. Variable temperature ^{13}C CP NMR spectra of ***pX@EtP6*** collected at the temperatures given in the figure. Data shown in black and blue were obtained at 9.4 T and 14.1 T, respectively. Spectral assignments are given in the figure (see **Figure 1**) and are derived from known isotropic chemical shifts,[10] ^{13}C-edited experiments (**Figure S8**) and PDLF data (see manuscript). Red daggers (†) and asterisks (*) denote signals arising from the *para*-xylene guest and spinning sidebands, respectively.

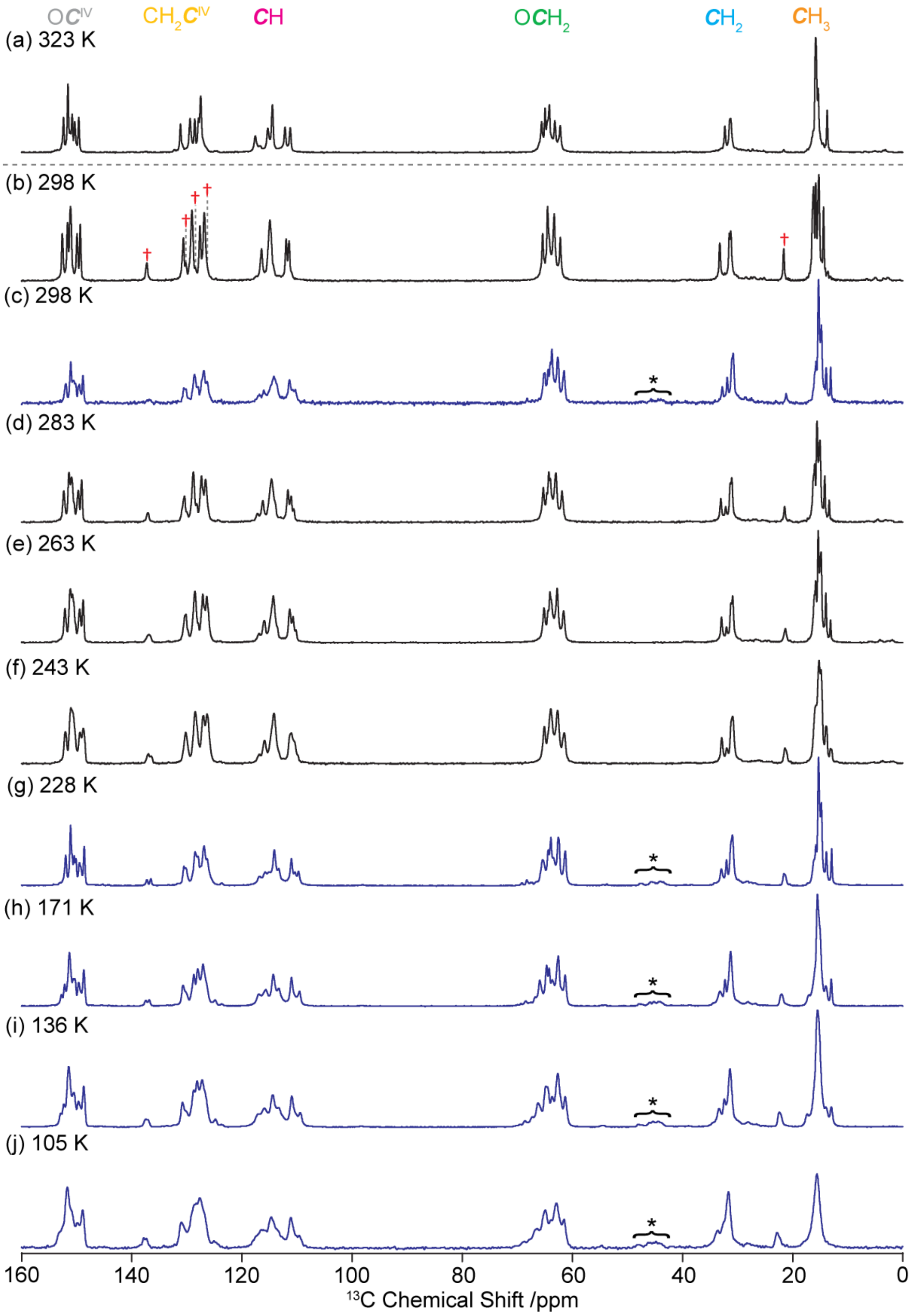

Figure S17. Variable temperature ^{13}C CP NMR spectra of ***m*X@EtP6** collected at the temperatures given in the figure. Data shown in black and blue were obtained at 9.4 T and 14.1 T, respectively. Spectral assignments are given in the figure (see **Figure 1**) and are derived from the known isotropic chemical shifts,[10] ^{13}C-edited experiments (**Figure S9**) and PDLF data (see manuscript). The dashed line indicates that *meta*-xylene signals are mostly lost between room temperature and 323 K, this temperature being in the range of the weight loss in the TGA data (**Figure S3**). It is likely that the *meta*-xylene remains in the rotor as a liquid phase that would not be detectable under the CP conditions used. Red daggers (†) and asterisks (*) denote signals arising from the *meta*-xylene guest and spinning sidebands, respectively.

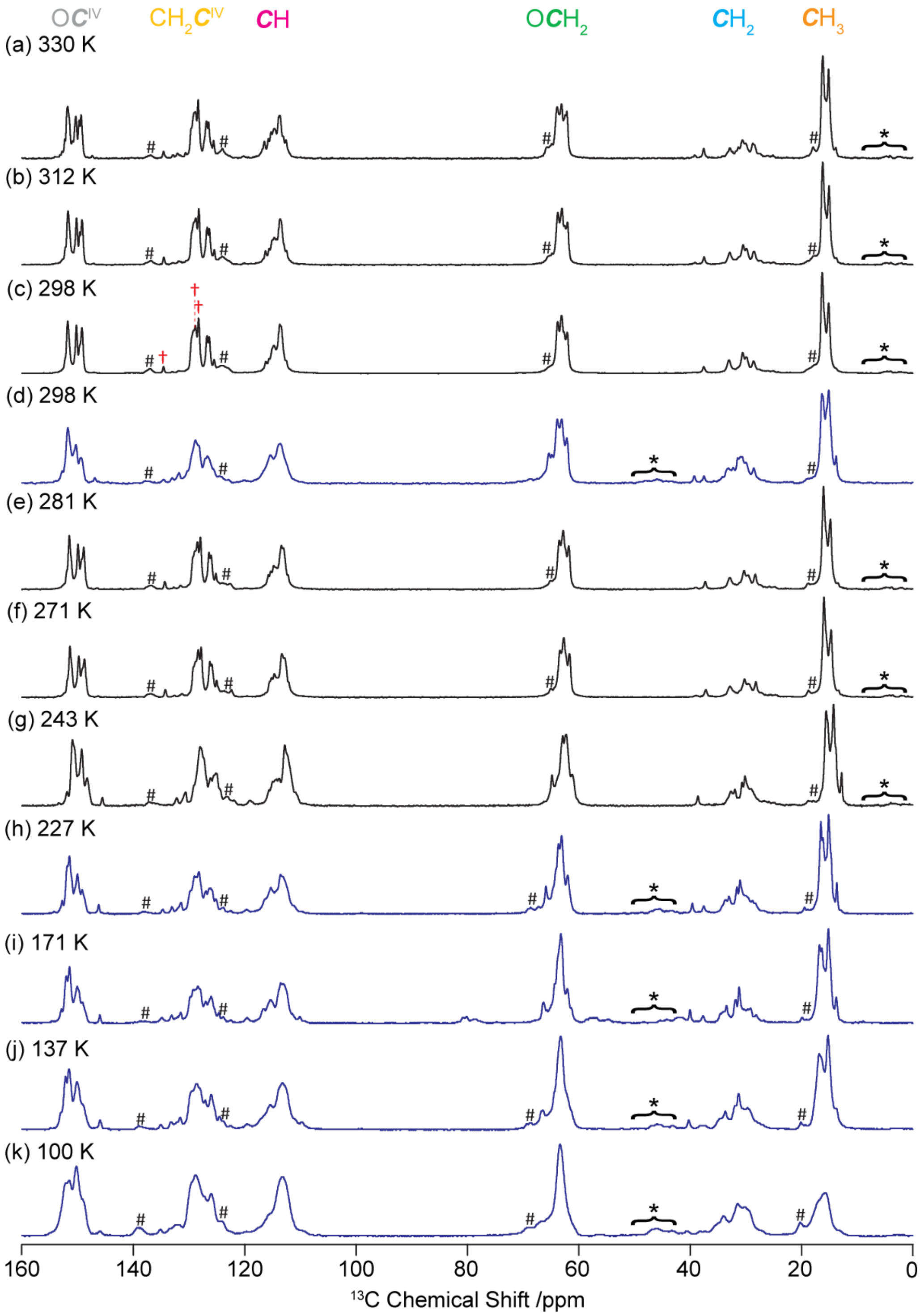

Figure S18. Variable temperature ^{13}C CP NMR spectra of ***oX@EtP6*** collected at the temperatures given in the figure. Data shown in black and blue were obtained at 9.4 T and 14.1 T respectively. Spectral assignments are given in the figure (see **Figure 1**) and are derived from known isotropic chemical shifts,[10] ^{13}C-edited experiments (**Figure S10**) and PDLF data (see manuscript). *Ortho*-xylene is not lost at 330 K as this process occurs above 340 K (see TGA data in **Figure S4**). Broad signals tentatively assigned to some unknown amorphous phase(s) are marked with dashes (#). Red daggers (†) and asterisks (*) denote signals arising from the *ortho*-xylene guest and spinning sidebands, respectively.

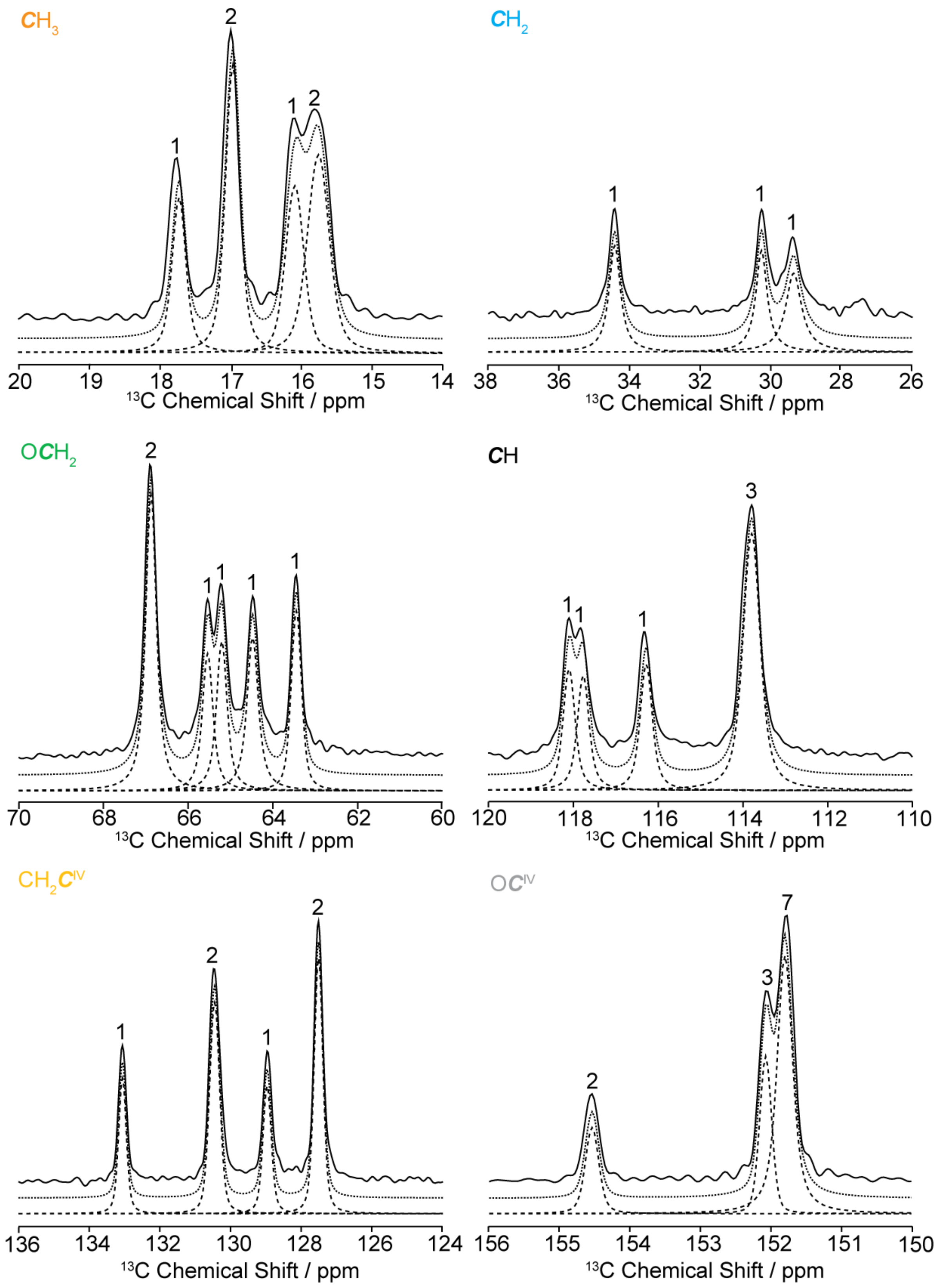

Figure S19. Experimental ^{13}C CP MAS NMR spectrum of **EtP6** (full line) obtained at 383 K and 9.4 T with the total fit and spectrum deconvolutions shown with dotted and dashed lines, respectively Integration values for each carbon subgroup are given. Each panel shows chemically distinct carbons environments of EtP6. Spectral assignments are given in the figure (see **Figure 1**). Integrations match with half the number of non-equivalent carbon atoms in the asymmetric unit cell of **EtP6-*β***.

S9. Additional proton detected local field NMR spectra

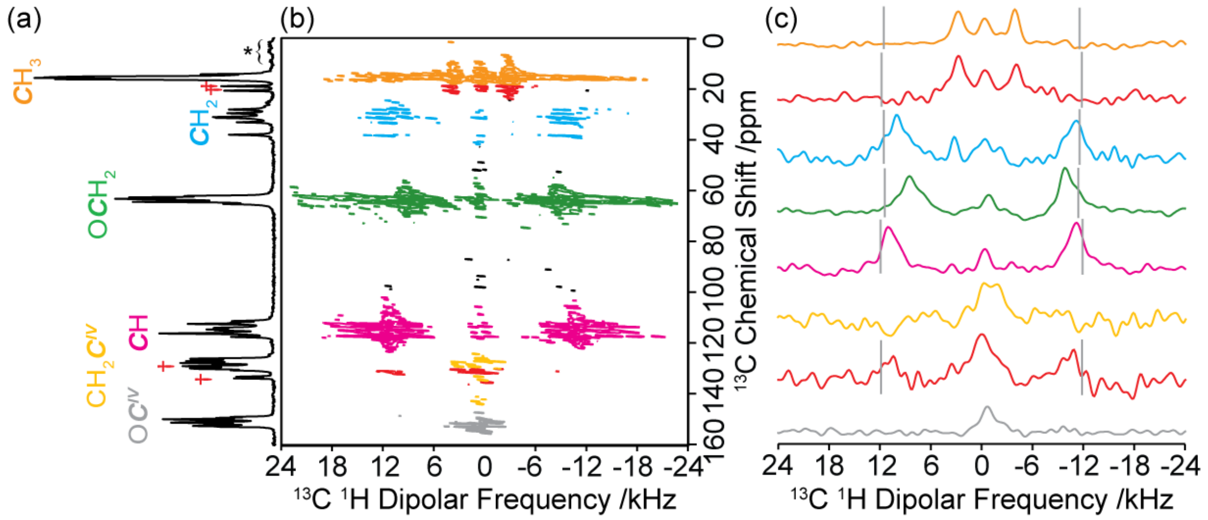

Figure S20. (a) ^{13}C CP MAS spectrum, (b) PDLF spectrum and (c) selected site-specific ^{13}C ^{1}H dipolar spectra for ***p*X@EtP6**. Spectral assignments are given in the figure and correspond to those previously published.[1] The data presented above was obtained at 298 K and 9.4 T. $\langle d_{CH} \rangle$ is measured using the outer singularities of the dipolar coupling spectra as highlighted in the experimental section. Vertical light grey lines indicate the static limit dipolar coupling constants d_{CH} calculated from **equation 1** and the computed CH distances obtained at the DFT level on the various conformers identified by CSP. Red daggers (†) and asterisks (*) denote signals arising from the *para*-xylene guest and spinning sidebands, respectively.

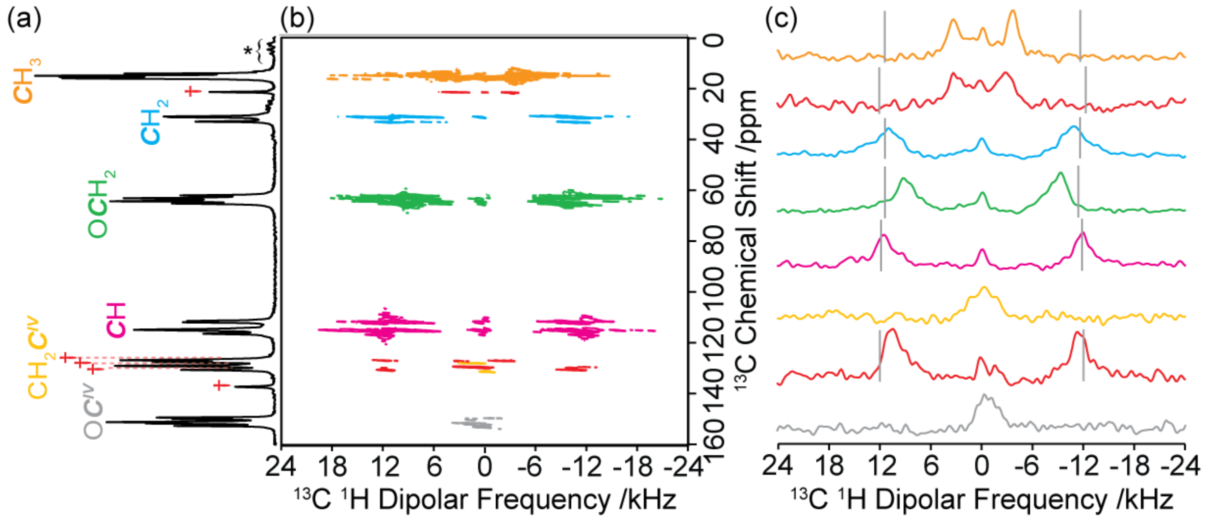

Figure S21. (a) ^{13}C CP MAS spectrum, (b) PDLF spectrum and (c) selected site-specific ^{13}C ^{1}H dipolar spectra for ***m*X@EtP6**. Spectral assignments are given in the figure and correspond to those previously published.[1] The data presented above was obtained at 298 K and 9.4 T. <d_{CH}> is measured using the outer singularities of the dipolar coupling spectra as highlighted in the experimental section. Vertical light grey lines indicate the static limit dipolar coupling constants d_{CH} calculated from **equation 1** and the computed CH distances obtained at the DFT level on the various conformers identified by CSP. Red daggers (†) and asterisks (*) denote signals arising from the *meta*-xylene guest and spinning sidebands, respectively.

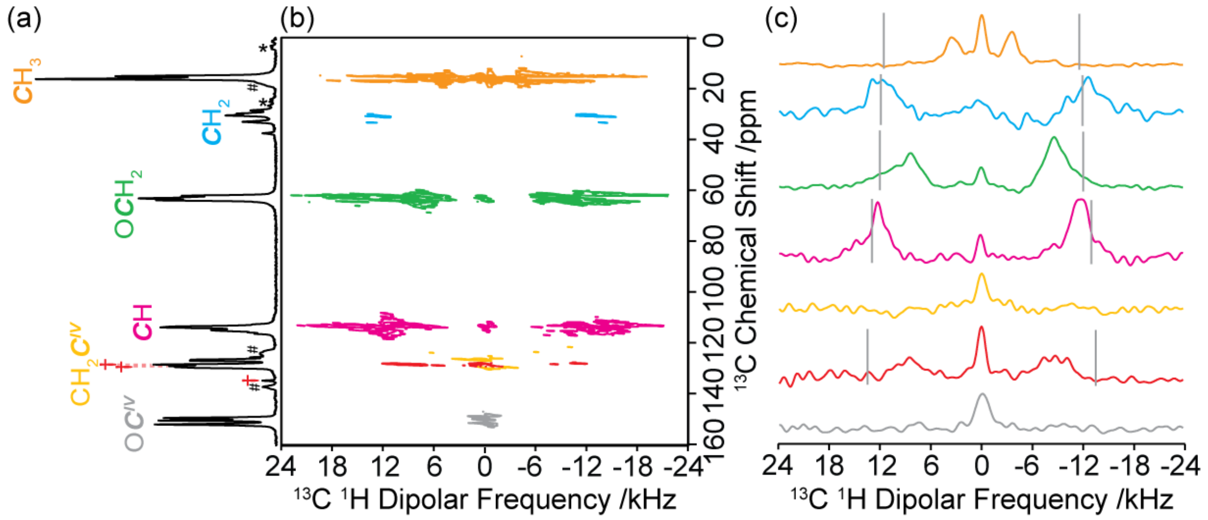

Figure S22. (a) ^{13}C CP MAS spectrum, (b) PDLF spectrum and (c) selected site-specific ^{13}C ^{1}H dipolar spectra for ***o*X@EtP6**. Spectral assignments are given in the figure and correspond to those previously published.[1] The data presented above was obtained at 298 K and 9.4 T. <d_{CH}> is measured using the outer singularities of the dipolar coupling spectra as highlighted in the experimental section. Vertical light grey lines indicate the static limit dipolar coupling constants d_{CH} calculated from **equation 1** and the computed CH distances obtained at the DFT level on the various conformers identified by CSP. Broad signals tentatively assigned to some unknown amorphous phase(s) are marked with dashes (#). Red daggers (†) and asterisks (*) denote signals arising from the *ortho*-xylene guest and spinning sidebands, respectively.

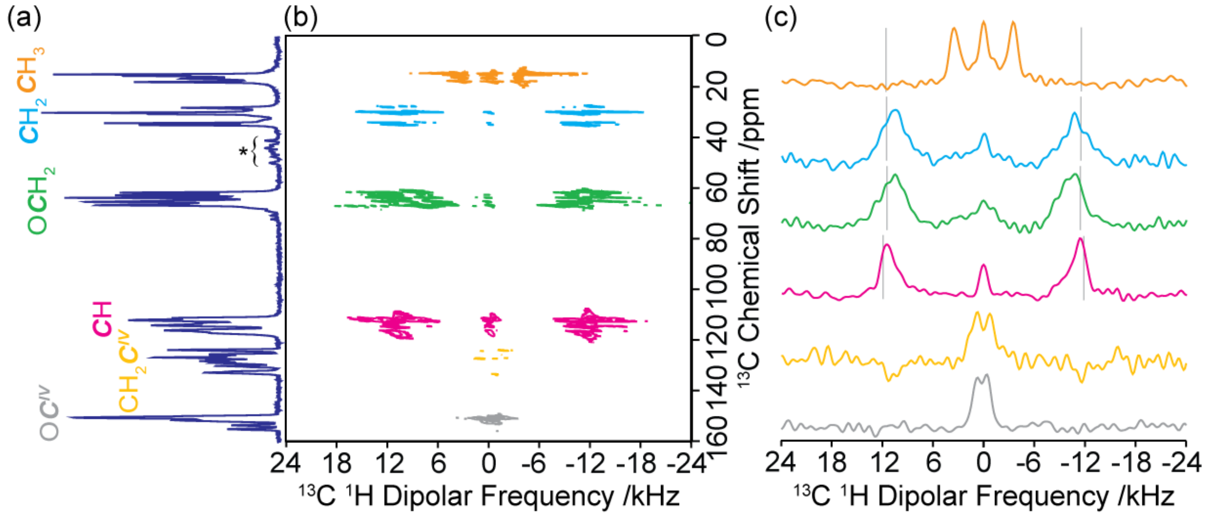

Figure S23. (a) ^{13}C CP MAS spectrum, (b) PDLF spectrum and (c) selected site-specific ^{13}C ^{1}H dipolar spectra for guest-free **EtP6-*β***. Spectral assignments are given in the figure and correspond to those previously published.[1] The data presented above was obtained at 100 K and 9.4 T. <d_{CH}> is measured using the outer singularities of the dipolar coupling spectra as highlighted in the experimental section. Vertical light grey lines indicate the static limit dipolar coupling constants d_{CH} calculated from **equation 1** and the computed CH distances obtained at the DFT level on the various conformers identified by CSP. Asterisks (*) denote spinning sidebands.

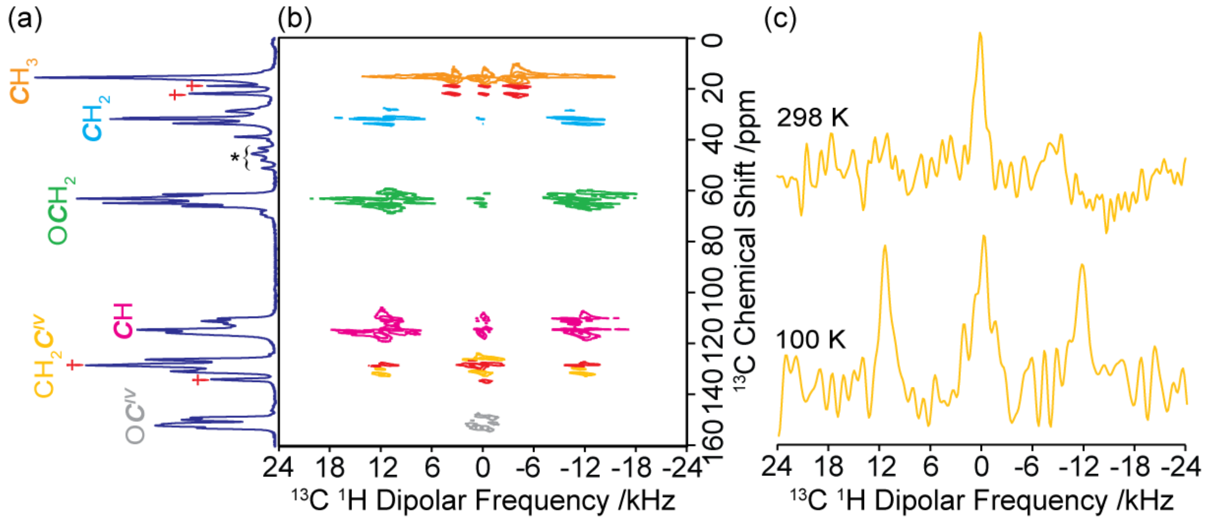

Figure S24. (a) ^{13}C CP MAS spectrum at 100 K, (b) PDLF spectrum at 100 K and (c) comparison of selected site-specific ^{13}C ^{1}H dipolar spectra for the host CH_2***C***^{IV} quaternary carbon in ***pX@EtP6*** obtained at 298 K and 100 K. Data presented above were obtained at 14.1 T. Spectral assignments are given in the figure. Signals arising from the adsorbed *para*-xylene guest are assigned with red daggers (†). Vertical light grey lines in the spectra indicate the static limit dipolar coupling constants d_{CH}. Asterisks (*) denote spinning sidebands.

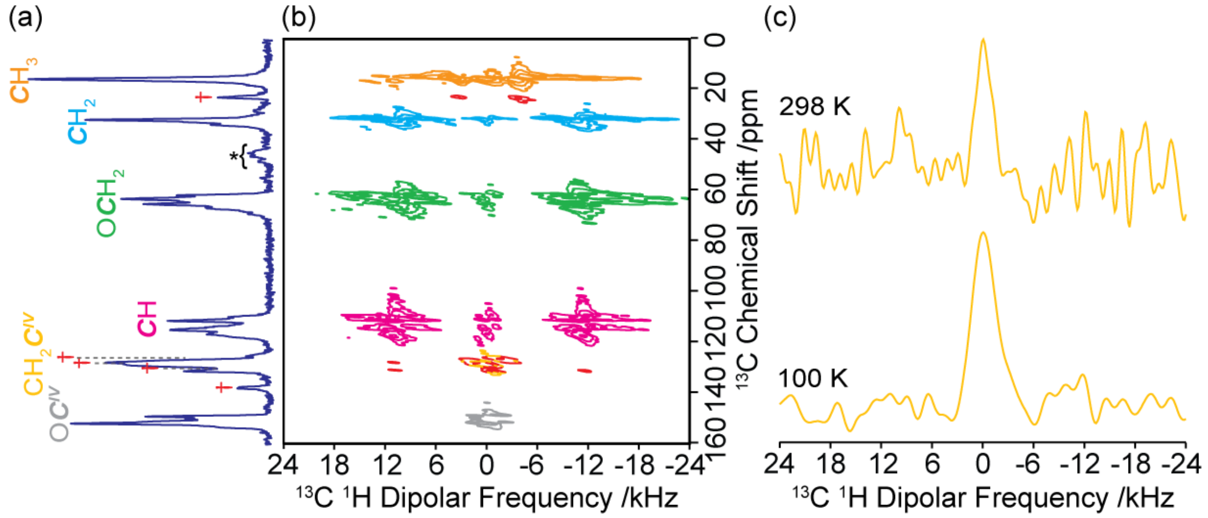

Figure S25. (a) ^{13}C CP MAS spectrum at 100 K, (b) PDLF spectrum at 100 K and (c) comparison of selected site-specific ^{13}C ^{1}H dipolar spectra for the host CH_2***C***^{IV} quaternary carbon in ***m*X@EtP6** obtained at 298 and 100 K. Data presented above were obtained at 14.1 T. Spectral assignments are given in the figure. Signals arising from the adsorbed *meta*-xylene guest are assigned with red daggers (†). No 2D PDLF signal is observed for the quaternary carbon of the *meta*-xylene due to poor signal to noise. Vertical light grey lines in the spectra indicate the static limit dipolar coupling constants d_{CH}. Asterisks (*) denote spinning sidebands.

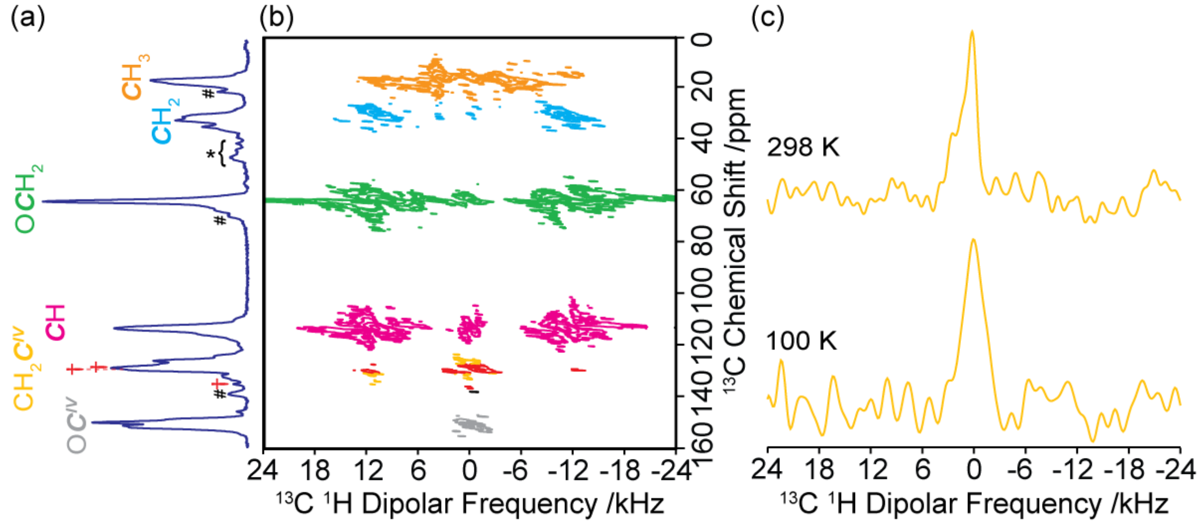

Figure S26. (a) ^{13}C CP MAS spectrum at 100 K, (b) PDLF spectrum at 100 K and (c) comparison of selected site-specific ^{13}C ^{1}H dipolar spectra for the host $CH_2\boldsymbol{C}^{IV}$ s quaternary carbon in ***o*****X@EtP6**. Data presented above were obtained at 14.1 T. Spectral assignments are given in the figure. Signals arising from the adsorbed *ortho*-xylene guest are assigned with red daggers (†), except the corresponding methyl resonance which is not directly attributable (see text). Broad signals tentatively assigned to some unknown amorphous phase(s) are marked with dashes (#). Vertical light grey lines in the spectra indicate the static limit dipolar coupling constants d_{CH}. Asterisks (*) denote spinning sidebands.

S10. Additional ^{1}H and ^{13}C spin lattice relaxation rates T_1^{-1} vs. temperatures

The ^{1}H spin lattice relaxation rates T_1^{-1}s have been obtained versus temperatures in the 383 - 243 K and 298 - 100 K temperature range at 9.4 T (**Figures S27(a)**, $\nu_0(^{1}H)$ = 400 MHz, $\nu_0(^{13}C)$ = 100 MHz) and 14.1 T (**Figures S28(a)**, $\nu_0(^{1}H)$ = 600 MHz, $\nu_0(^{13}C)$ = 150 MHz), respectively, for **EtP6-*β***, all xylene-adsorbed EtP6 adducts and **EtP5-*α*** (data only available at 9.4 T for this phase). The corresponding ^{1}H MAS NMR spectra of all materials are broadened by ^{1}H ^{1}H homonuclear dipolar coupling and are poorly resolved as discussed in Section S11 (^{1}H MAS spectra under various experimental conditions are given in **Figures S32-S36**) hence ^{1}H T_1 values are obtained from spectral integration of the entire spectra. Additionally, the ^{1}H T_1's are fairly long (3 s for **EtP6-*β*** at 298 K and 9.4 T) and ^{1}H ^{1}H spin diffusion results in an equilibration of the T_1 values for all resonances so that it is likely that the ^{1}H T_1s detect the same motional processes. At 14.1 T and for all materials, the ^{1}H T_1^{-1} values increase with increasing temperatures, reaching a maximum around 160 K for ***p*X@EtP6**, 170 K for ***m*X@EtP6** and 190 K for both **EtP6-*β*** and ***o*X@EtP6**, and then decreasing above these temperatures (**Figure S28(a)**). At these T_1^{-1} maxima, the motion is near the ^{1}H Larmor frequency $\omega_{0,H}$ (in rad.s^{-1}) with the following expression being satisfied:[11]

$$\omega_{0,H}\tau_c \approx 0.62 \quad \textbf{(S3)}$$

from which a τ_c value of 2.5 x10^{-10} s at the aforementioned temperatures for the named materials could be obtained for ^{1}H at 14.1 T. The ^{1}H T_1^{-1}s maxima are not observed in the 383 - 243 K temperature range accessible on the MAS probe at 9.4 T which is in agreement with the largely magnetic field independence of dipolar coupling driven T_1s values as illustrated in **Figures S28(a)** and **S29(a)**, and **equation S4** (see below).

Importantly, there is a marked dependency of the T_1^{-1} maxima temperature with the materials under consideration which decrease from ~190 K for **EtP6-*β*** and ***o*X@EtP6** to ~170 K for ***m*X@EtP6** and ~160 K for ***p*X@EtP6**. It is well known that the position of the T_1^{-1} maximum depends on τ_c with colder temperatures required to satisfy **equation S3** for larger molecular size resulting in slower tumbling.[11] The temperature dependency of the T_1^{-1} maximum therefore indicates decreasing tumbling rates for **EtP6-*β*** and ***o*X@EtP6** to ***m*X@EtP6** and to ***p*X@EtP6** that are associated with larger molecular assemblies. These suggest that *ortho*-xylene has little effect on the relaxation rates of the host due to its larger spatial freedom as observed above from xylene CH dipolar coupling experiments, whereas both *meta*-xylene and *para*-xylene shift this maximum towards lower temperatures (170 K and 160 K, respectively) suggesting that the filling of the pillar[6]arene void upon loading of *meta*-xylene and the adaptive adsorption of *para*-xylene results in an overall smaller conformation.

It is assumed that the ^{1}H relaxation is driven by ^{1}H-^{1}H homonuclear dipole-dipole coupling and the longitudinal relaxation rates T_1^{-1} can be related to τ_c with the following equation (in the approximation of isotropic motion):[11,12]

$$\frac{1}{T_1} = \frac{3}{10}\left(\frac{\mu_0}{4\pi}\right)^2 \gamma_H^4 \hbar^2 \left(\sum_i \frac{1}{r_i^6}\right)\left[\frac{\tau_c}{1+\omega_{0,H}^2\tau_c^2} + \frac{4\tau_c}{1+4\omega_{0,H}^2\tau_c^2}\right] \quad \textbf{(S4)}$$

by summing over all the effective ^{1}H-^{1}H internuclear distances r_i. The local magnetic fields fluctuation term of this expression is proportional to the square of the dipole-dipole coupling constant while the terms in squared bracket are the spectral density functions. All other possible relaxation mechanisms for a spin 1/2 nucleus such as those driven by CSA, spin-rotation or scalar coupling are likely quite small for ^{1}H to satisfy the frequencies for relaxation and have therefore been neglected.

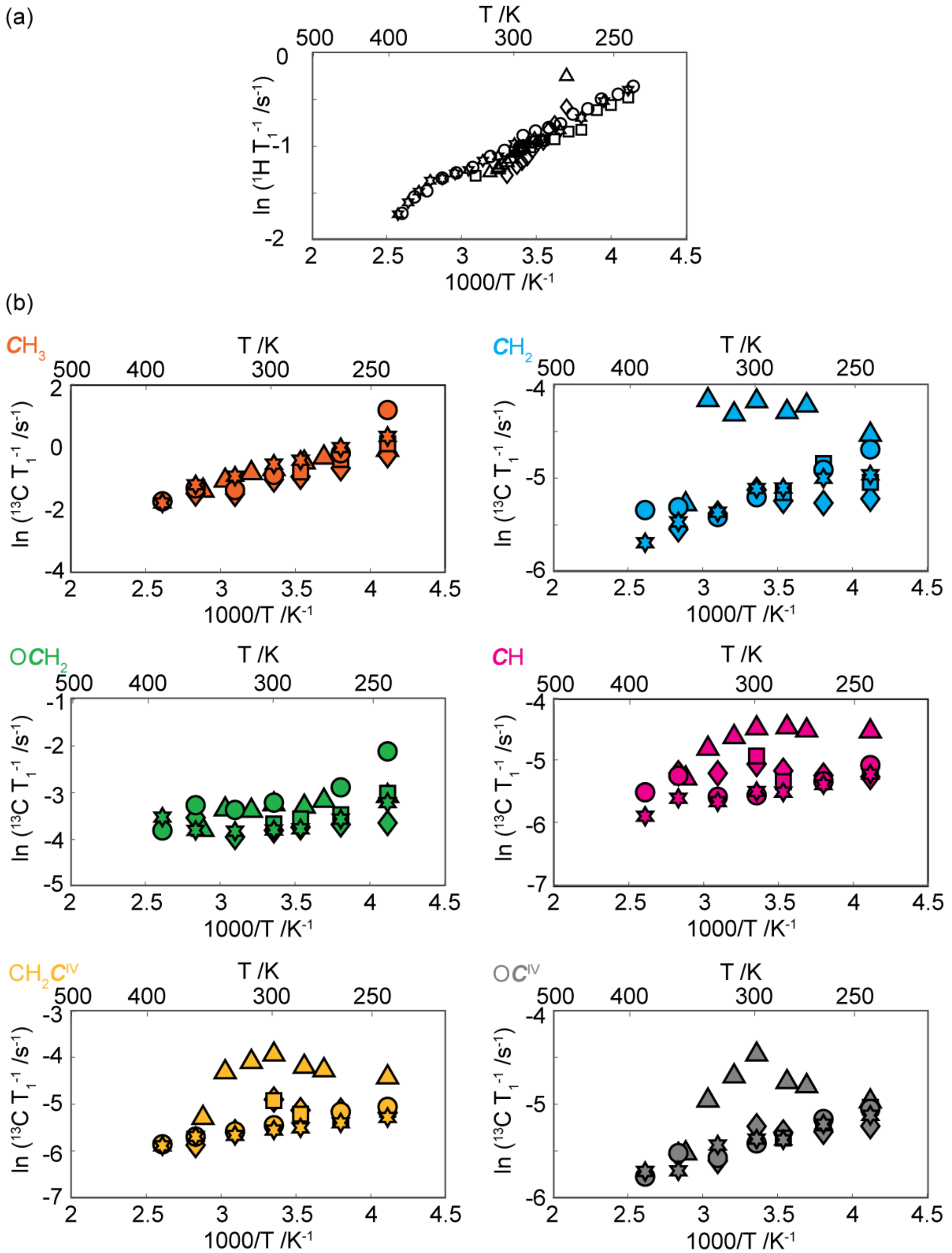

Figure S27. (a) ^{1}H and (b) ^{13}C spin–lattice relaxation rates (T_1^{-1}) Arrhenius plots. Data were obtained at 9.4 T for guest-free **EtP5-α** (stars), guest-free **EtP6-β** (circles), ***p*X@EtP6** (squares), ***m*X@EtP6** (diamonds) and ***o*X@EtP6** (triangles). The data were fitted to a stretch exponential function in the form of $1-\exp[-(\tau/T_1)^{\alpha}]$ and the errors associated are given to a 95% confidence level and are smaller than the symbol sizes.

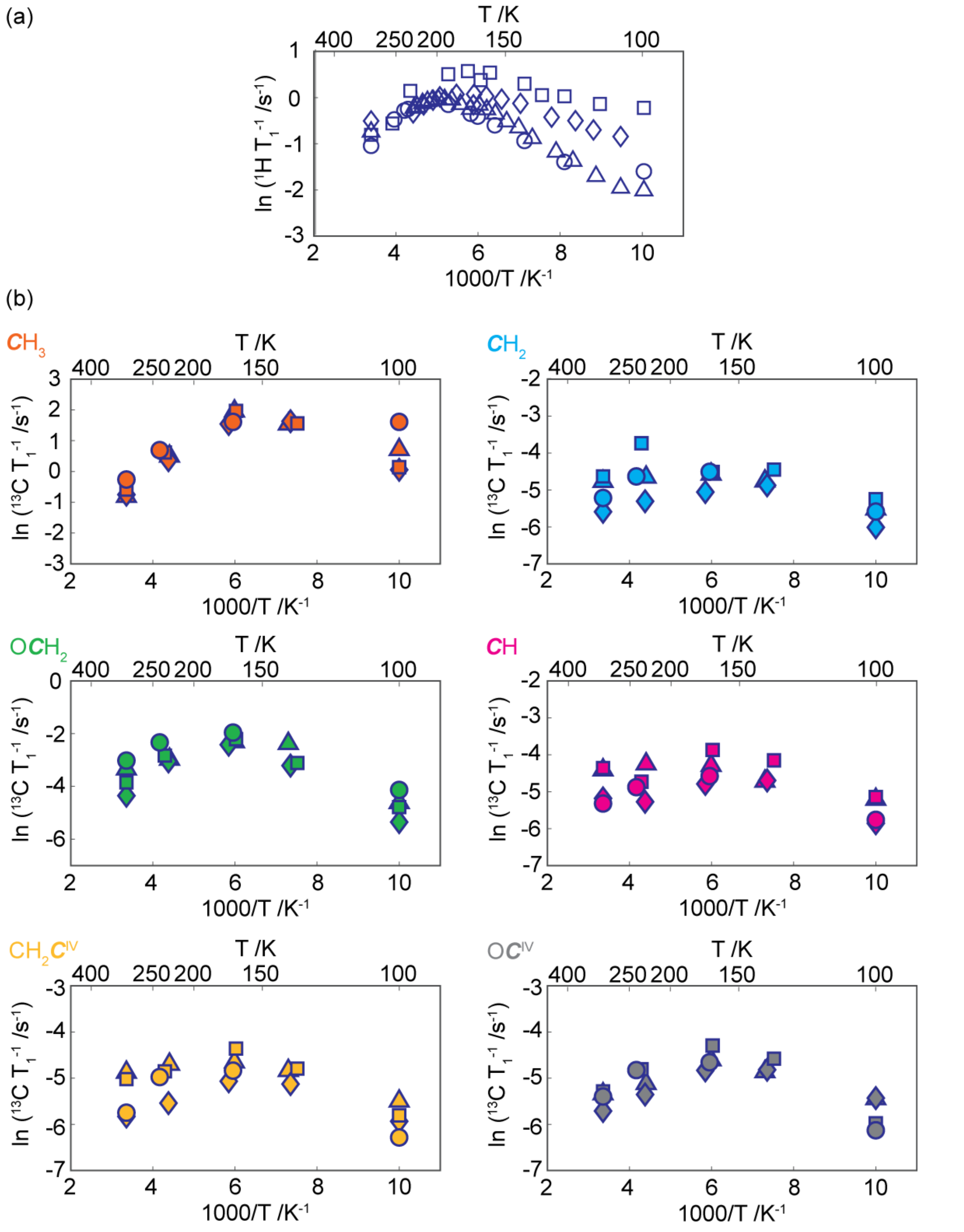

Figure S28. (a) ^{1}H and (b) ^{13}C spin–lattice relaxation rates (T_1^{-1}) Arrhenius plots. Data were obtained at 14.1 T for **EtP6-β** (circles), ***p*X@EtP6** (squares), ***m*X@EtP6** (diamonds) and ***o*X@EtP6** (triangles). The data were fitted to a stretch exponential function in the form of 1-exp[-(τ/T_1)$^{\alpha}$] and the errors associated are given to a 95% confidence level and are smaller than the symbol sizes.

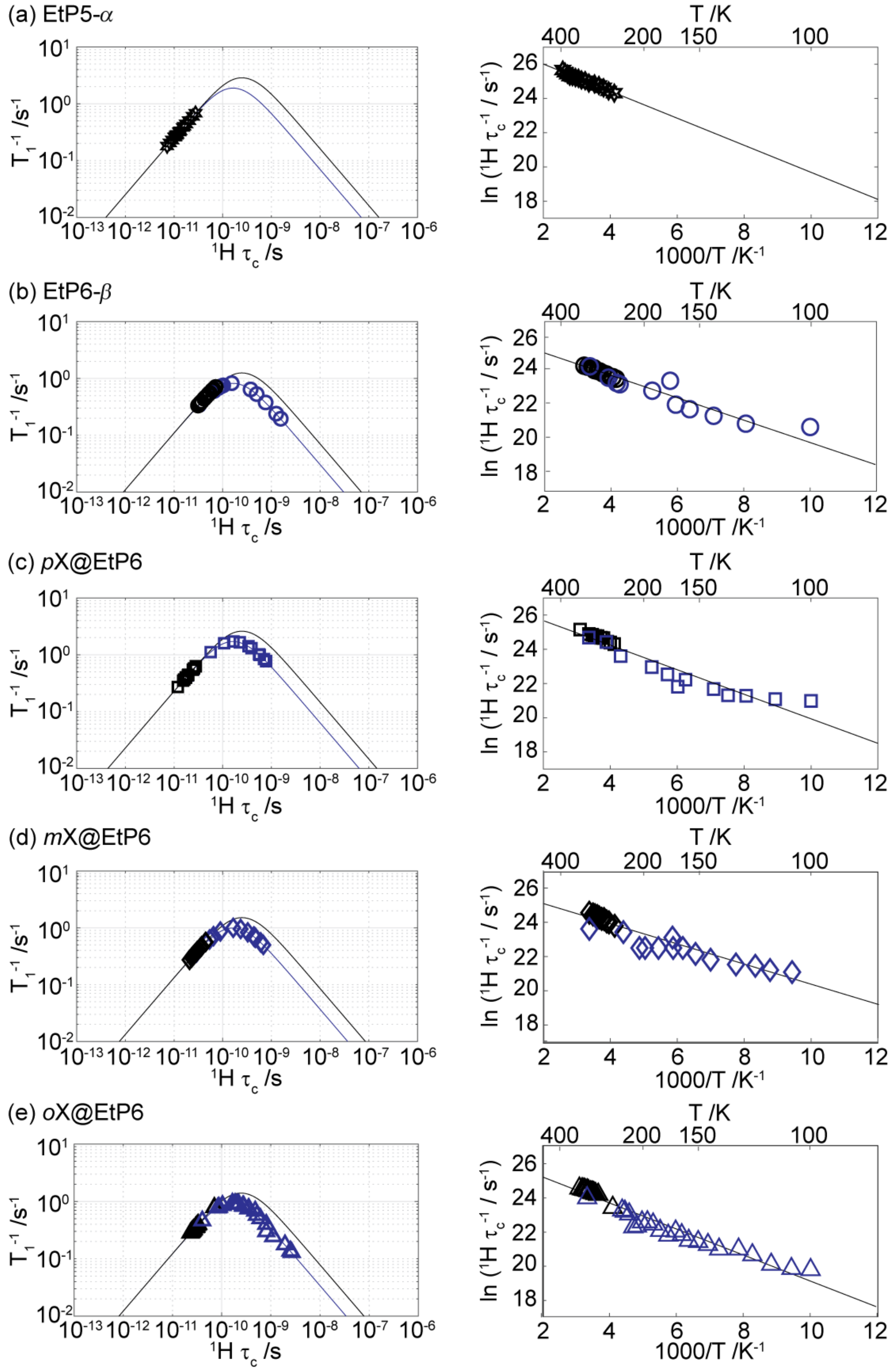

Figure S29. (Left) ¹H spin–lattice relaxation rates T_1^{-1} against correlation times τ_c and (right) corresponding ¹H correlation frequencies τ_c^{-1} Arrhenius plots. Data shown in black and blue were obtained at 9.4 T and 14.1 T, respectively, for (a) guest-free **EtP5-*α*** (stars), (b) guest-free **EtP6-*β*** (circles), (c) ***p*X@EtP6** (squares), (d) ***m*X@EtP6** (diamonds) and (e) ***o*X@EtP6** (triangles). The associated errors are smaller than the symbol sizes. The solid (-) lines in the left panels are those obtained from a dipolar coupling relaxation mechanism (**equation S4**) at both fields using experimentally determined local field fluctuations terms of 2 $x10^9$, 5 $x10^9$, 3 $x10^9$ and 2 $x10^9$ s^{-2} for **EtP6-*β***, ***p*X@EtP6**, ***m*X@EtP6** and ***o*X@EtP6**, respectively (A calculated value of 5 $x10^9$ s^{-2} from the ¹H-¹H distances computed by CSP was used for **EtP5-*α*** as no T_1 minimum was found in the temperature range studied) and in the right panels are fit to the Arrhenius equation.

Combining **equations S3** and **S4** above at the temperatures of the ^{1}H T_1 minimum allows the determination of the local dipolar magnetic fields fluctuation term to be determined. Experimental values of 2 x10^{9} s^{-2} for **EtP6-*β*** and ***oX*@EtP6**, 3 x10^{9} s^{-2} for ***mX*@EtP6** and 5 x10^{9} s^{-2} for ***pX*@EtP6** were obtained which compares extremely well with the value of 5 x10^{9} s^{-2} calculated for all materials from the explicit expression of the local fields and using the shortest ^{1}H-^{1}H distances in the known CSP data of these phases.[2] No T_1 minimum is observed for **EtP5-*α*** in the temperature range studied preventing the local field term to be determined so this later calculated value was used.

Figure S29 displays the corresponding ^{1}H T_1^{-1} versus τ_c logarithmic plots for all samples at 9.4 T (black data) and 14.1 T (blue data). The T_1^{-1} and τ_c values extracted from this approach show no field dependence in the fast motion limit where τ_c are much shorter than the Larmor frequency *i.e.* $\omega_{0,H}\tau_c$ << 1 (left hand side of the T_1^{-1} maxima) as expected from **equation S4**. The τ_c's extracted from this approach do not vary significantly between the materials and suggest the same similar motional process. The temperature dependence of the correlation frequencies τ_c^{-1} was subsequently modelled with the Arrhenius equation (**equation 3** in the main text). Assuming a single activation energy, fit to τ_c^{-1} versus reciprocal temperatures (**Figure S29**) is satisfactory and the extracted E_as (**Table S7**) are small, suggesting similar facile motion of the pillar[n]arene assemblies. Upon adsorption of *para*-xylene and *ortho*-xylene into **EtP6-*β***, the activation barriers very slightly increase by *ca*. 0.5-0.8 kJ mol^{-1} while it decreases by 0.6 kJ mol^{-1} in the case of ***m*X@EtP6** material. The assumed isotropic motion and associated errors with these values imply that these E_as should simply be taken as an indication of possible motional effects of xylenes into **EtP6-*β***. In the case of ***p*X@EtP6** and ***o*X@EtP6**, it is postulated that their larger E_as *vs.* the one for **EtP6-*β*** capture increase in the steric hindrance of the assemblies upon adsorption of *para*-xylene and *ortho*-xylene (**Figures 1(c)** and **1(e)**), resulting in overall larger conformation of these materials and therefore slower overall dynamics when xylenes occupy (***p*X@EtP6**) or partially occupy (***o*X@EtP6**) the pillar[6]arene void. In contrast, the smaller E_a obtained for ***m*X@EtP6** could be potentially due to folding of the pillar[6]arene void upon *meta*-xylene adsorption (**Figure 1(d)**) and associated with the position of the *meta*-xylene on top of the pillar[6]arene core.

Table S7. Comparison of the attempt frequencies $\tau_{c,0}^{-1}$ and activation energy barriers E_a of guest-free **EtP5-*α***, guest-free **EtP6-*β***, ***p*X@EtP6**, ***m*X@EtP6** and ***o*X@EtP6** obtained from the Arrhenius plots of the ^{1}H correlation frequencies τ_c^{-1}.

Pillar[n]arenes	$\tau_{c,0}^{-1}$/s^{-1}	E_a/kJ mol^{-1} [a]
**EtP5-*α*** [b]	9 x10^{11}	7
**EtP6-*β***	2 x10^{11}	6
***p*X@EtP6**	6 x10^{11}	6
***m*X@EtP6**	2 x10^{11}	5
***o*X@EtP6**	4 x10^{11}	6

[a] Errors are in the order of 1 kJ mol^{-1}. [b] Data in the 383 - 243 K temperature range only available.

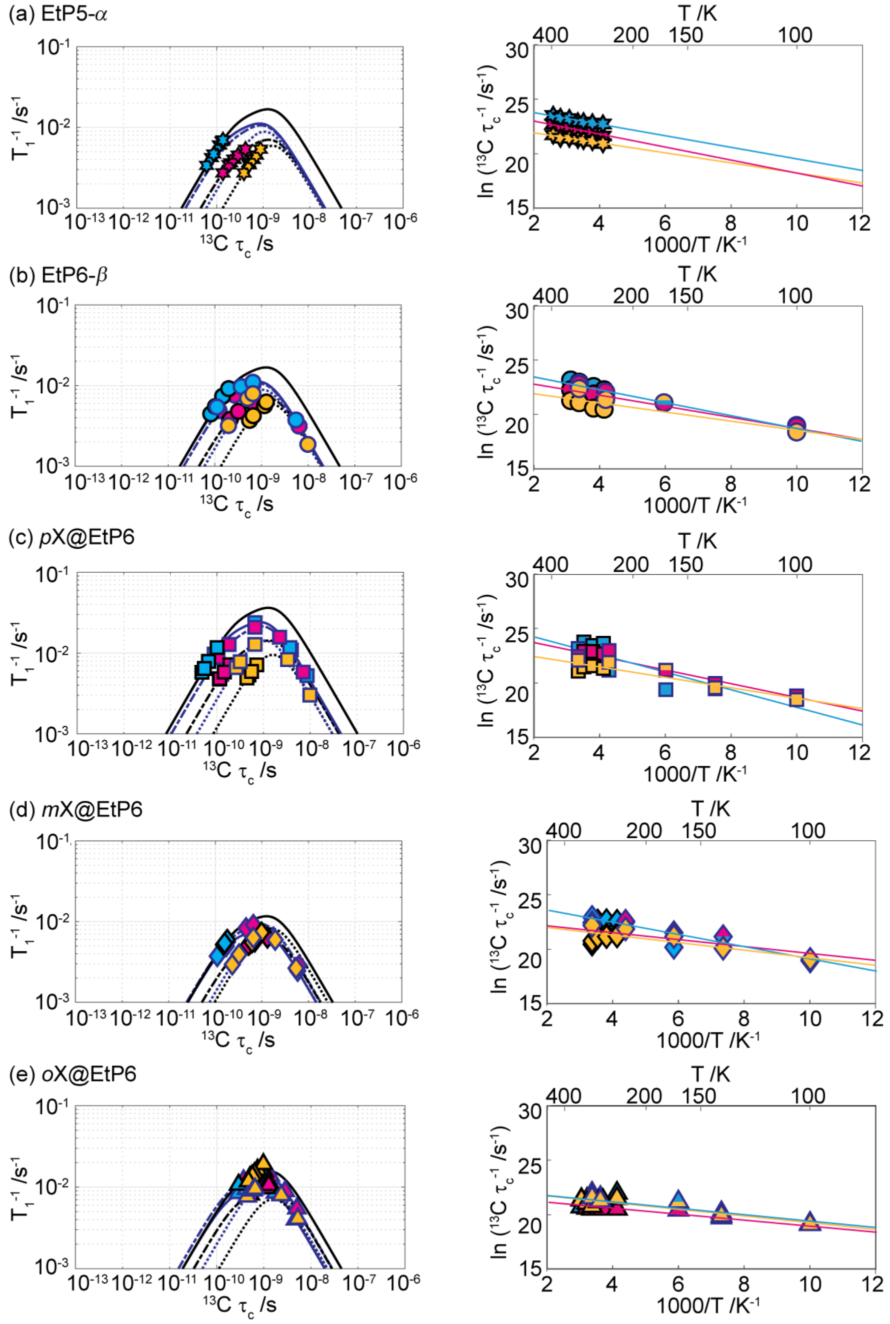

Figure S30. (Left) ^{13}C spin–lattice relaxation rates T_1^{-1} against correlation times τ_c and (right) corresponding ^{13}C correlation frequencies τ_c^{-1} Arrhenius plots. Data shown in black and blue outlines were obtained at 9.4 T and 14.1 T, respectively, for (a) guest-free **EtP5-*α*** (stars), (b) guest-free **EtP6-*β*** (circles), (c) ***p*X@EtP6** (squares), (d) ***m*X@EtP6** (diamonds) and (e) ***o*X@EtP6** (triangles). Selected carbon subgroups have been plotted here with the following colour coding for ***C***H_2 (light blue), ***C***H (pink) and CH_2***C***^{IV} (yellow) (**Figure 1**) whilst plot giving the three other carbons are given in **Figure 7**. The associated errors are smaller than the symbol sizes. In the left panels, the dashed (-·) lines are those obtained from dipolar coupling and CSA relaxation mechanisms (**equation 4**) for ***C***H (pink), the solid lines (-) from a dipolar coupling relaxation mechanism (**equation 5**) for ***C***H_2 (light blue) and the dotted (··) lines from a CSA relaxation mechanism (**equation 6**) for CH_2***C***^{IV} (yellow) at both fields, using the experimentally determined local magnetic fields terms (values from **EtP6-*β*** were used for **EtP5-*α*** as no T_1 minimum was found in the temperature range studied). A T_1 minima was found for O***C***^{IV} in ***o*X@EtP6** in the temperature range studied at 9.4 T, therefore this data was used to extract correlation times and is plotted for this series. In the right panels, the lines are fit to the experimental data using the Arrhenius equation.

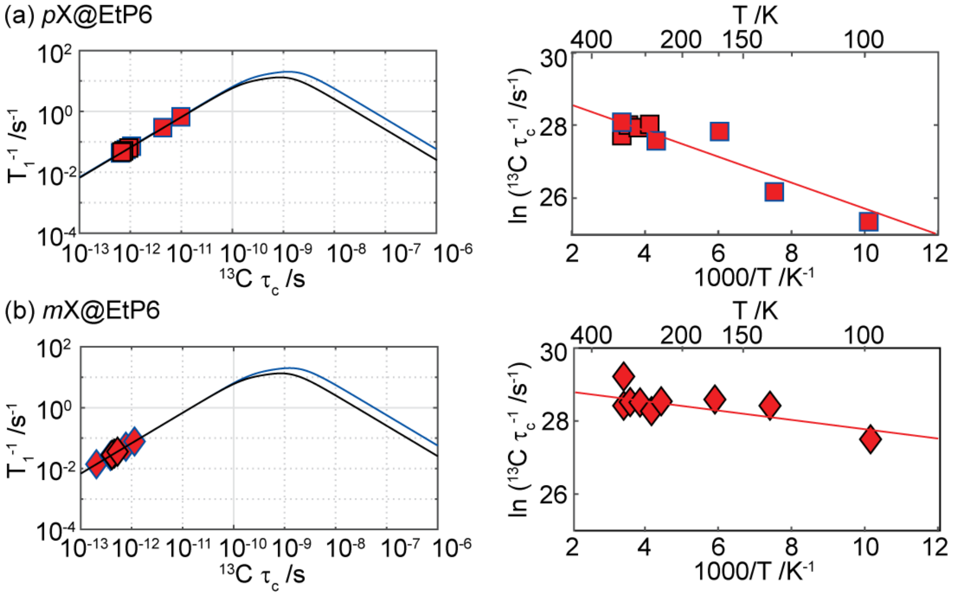

Figure S31. (Left) ^{13}C spin−lattice relaxation rates T_1^{-1} against correlation times τ_c and (right) corresponding ^{13}C correlation frequencies τ_c^{-1} Arrhenius plots. Data shown in black and blue outlines were obtained at 9.4 T and 14.1 T, respectively, for the xylene methyl groups in (a) ***pX@EtP6*** (squares) and (b) ***mX@EtP6*** (diamonds). Overlaps of the *ortho*-xylene methyl group with the host ***C***H_3 in ***oX@EtP6*** (**Figure S18**) prevent there T_1 to be measured. The associated errors are smaller than the symbol sizes. In the left panels, the solid (-) lines are those obtained from a dipolar coupling relaxation mechanism (**equation 5**) for ***C***H_3 at both fields, using the calculated local magnetic fields term and distances available in literature from diffraction data.[13] In the right panels, the lines are fit to the experimental data using the Arrhenius equation.

S11. Very high field and variable temperature ^{1}H NMR spectra

^{1}H one pulse spectra were obtained at 9.4 T for **EtP5-α**, **EtP6-β**, ***p*X@EtP6**, ***m*X@EtP6** and ***o*X@EtP6** as shown in panel (a) in **Figures S32-S36** respectively. Limited resolution allows for identification of very chemical distinct sites. Upon increasing the field to 20 T and increasing the MAS rate to 60 kHz, resolution of the ^{1}H one pulse spectra increases, to allow further sites to be identified (panel (b) in **Figures S32-S36**) however, due to the significant broadening due to ^{1}H ^{1}H dipolar coupling within ^{1}H spectra within the solid state, only limited information can be obtained. The ^{1}H one pulse spectra obtained upon variable temperature at 14.1 T are shown in panel (c) of **Figures S32-S36**. The T_1 values extracted from the corresponding variable temperature saturation recovery experiments are shown in **Figure S28(a)** and used to extract corresponding correlation times τ_c as discussed in the manuscript.

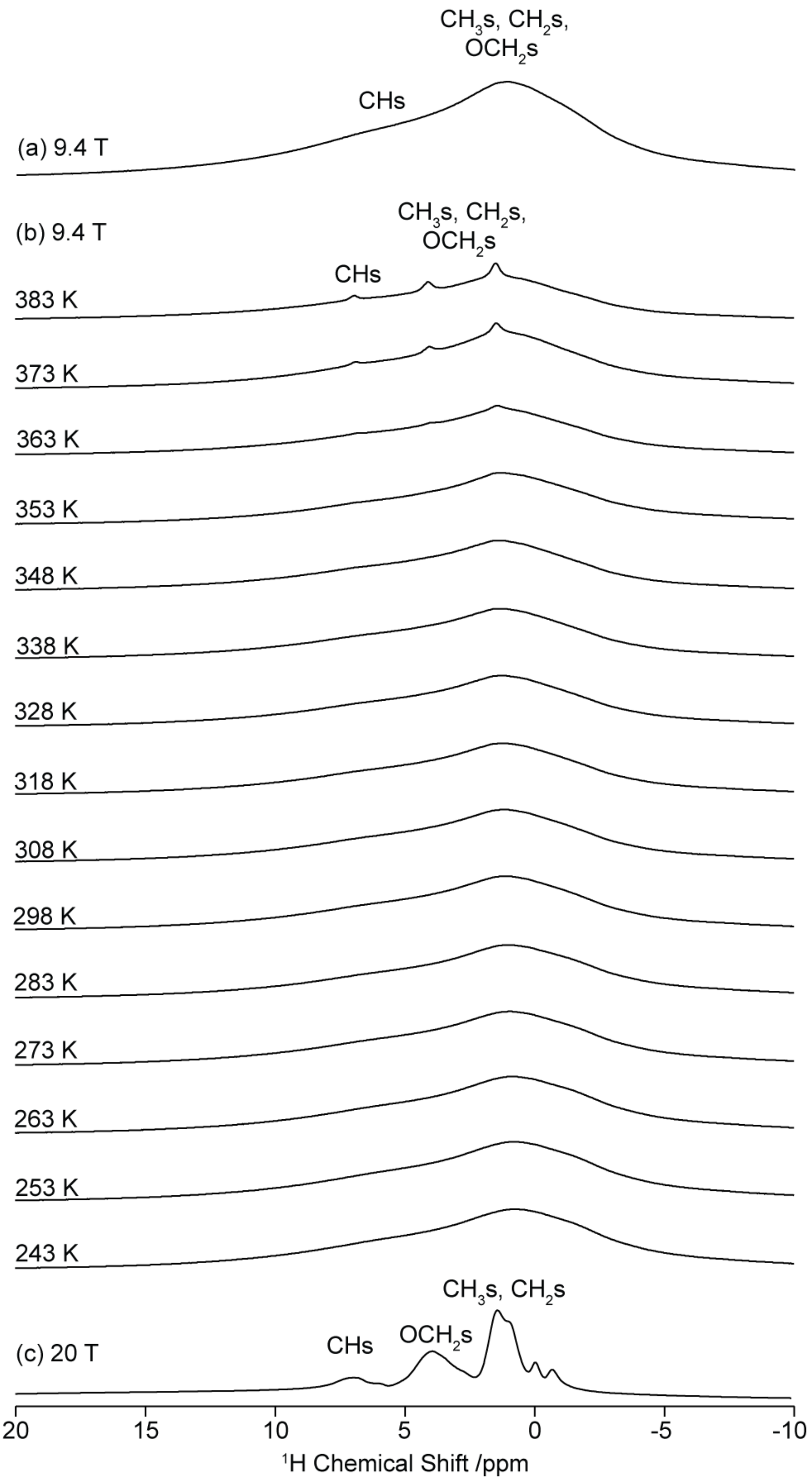

Figure S32. ^{1}H one pulse MAS NMR spectra of **EtP5-α** obtained (a) under MAS at ν_r = 12.5 kHz and 9.4 T, (b) under variable temperature conditions at ν_r = 12.5 kHz and 9.4 T and (c) at ν_r = 60 kHz at 20 T. Spectral assignments are given in the figure.

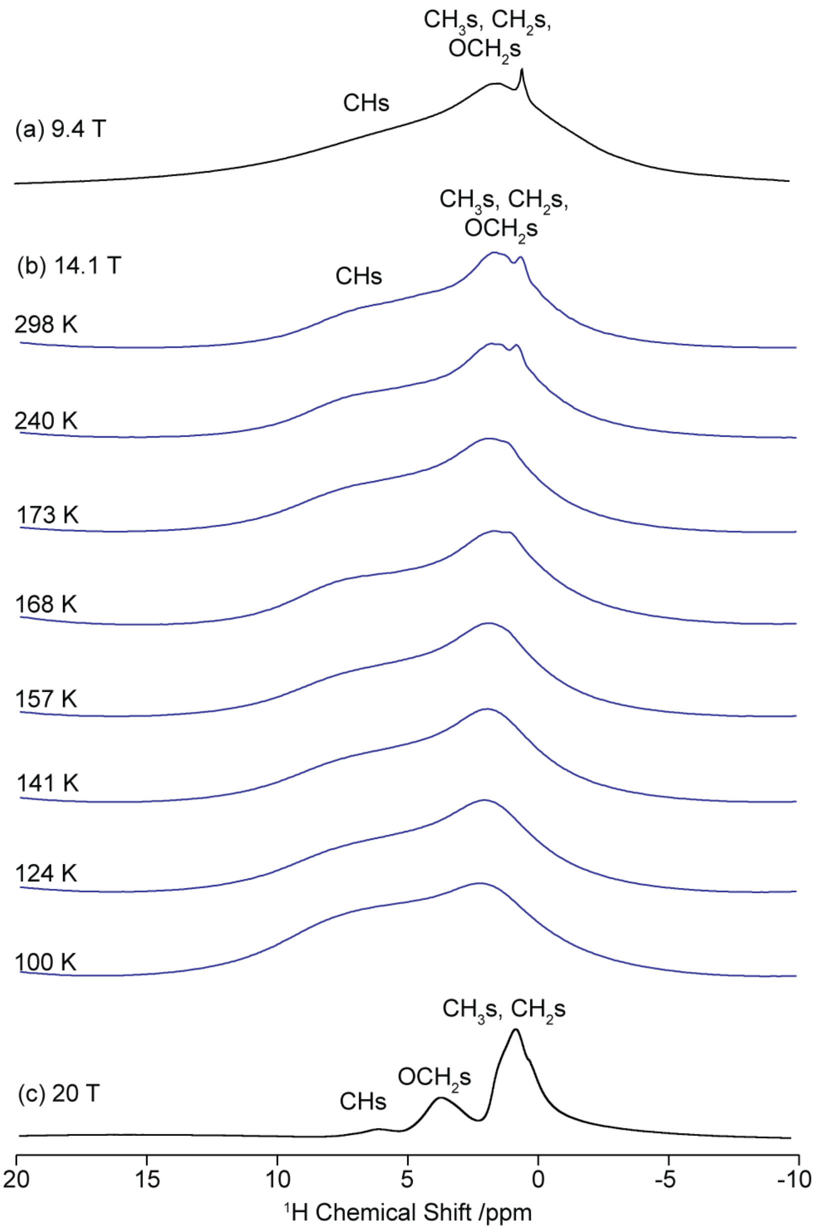

Figure S33. ^{1}H one pulse NMR spectra of **EtP6-β** (a) obtained at ν_r = 12.5 kHz and 9.4 T (b) obtained under variable temperature conditions at ν_r = 12.5 kHz and 9.4 T and (c) obtained at ν_r = 60 kHz at 20 T. Spectral assignments are given in the figure.

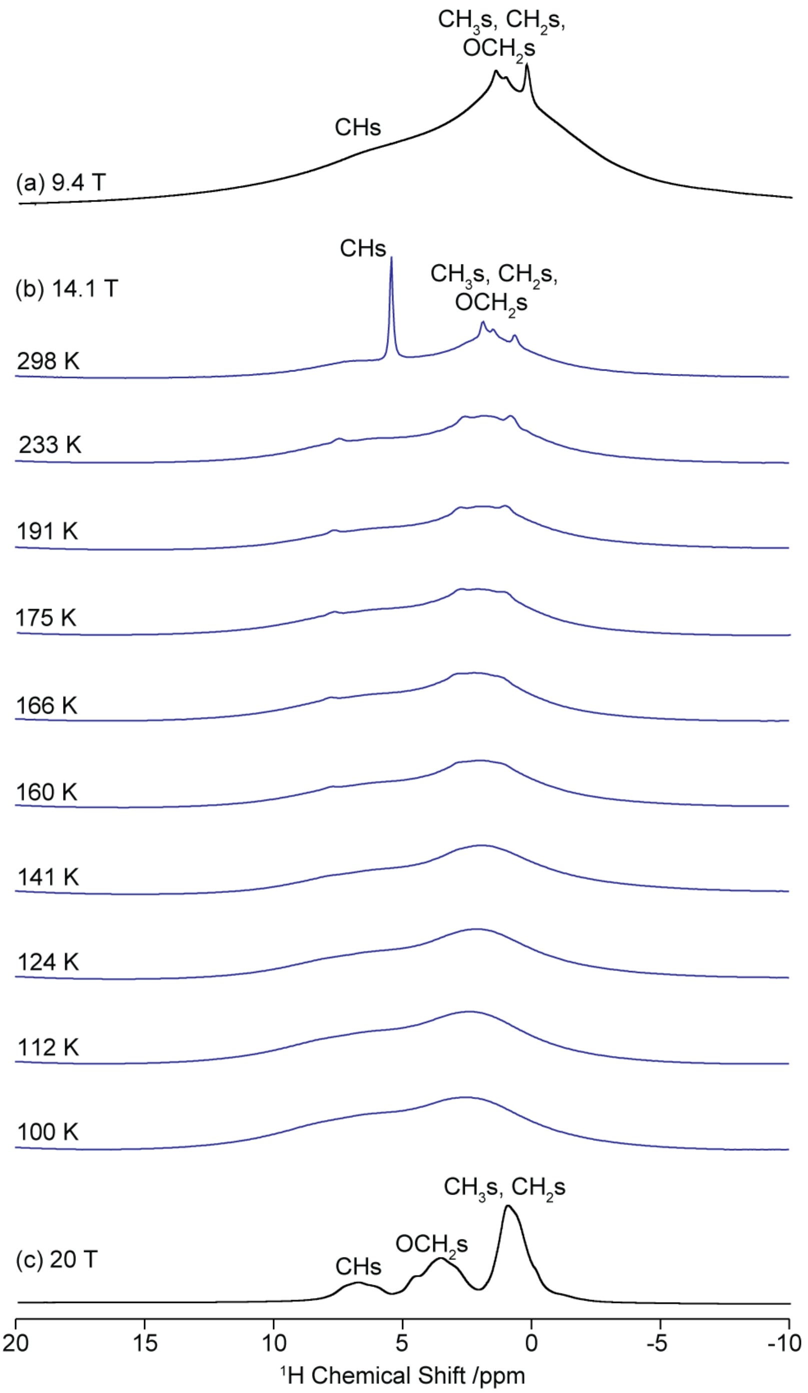

Figure S34. ^{1}H one pulse NMR spectra of ***pX@EtP6*** (a) obtained at ν_r = 12.5 kHz and 9.4 T (b) obtained under variable temperature conditions at ν_r = 12.5 kHz and 9.4 T and (c) obtained at ν_r = 60 kHz at 20 T. Spectral assignments are given in the figure.

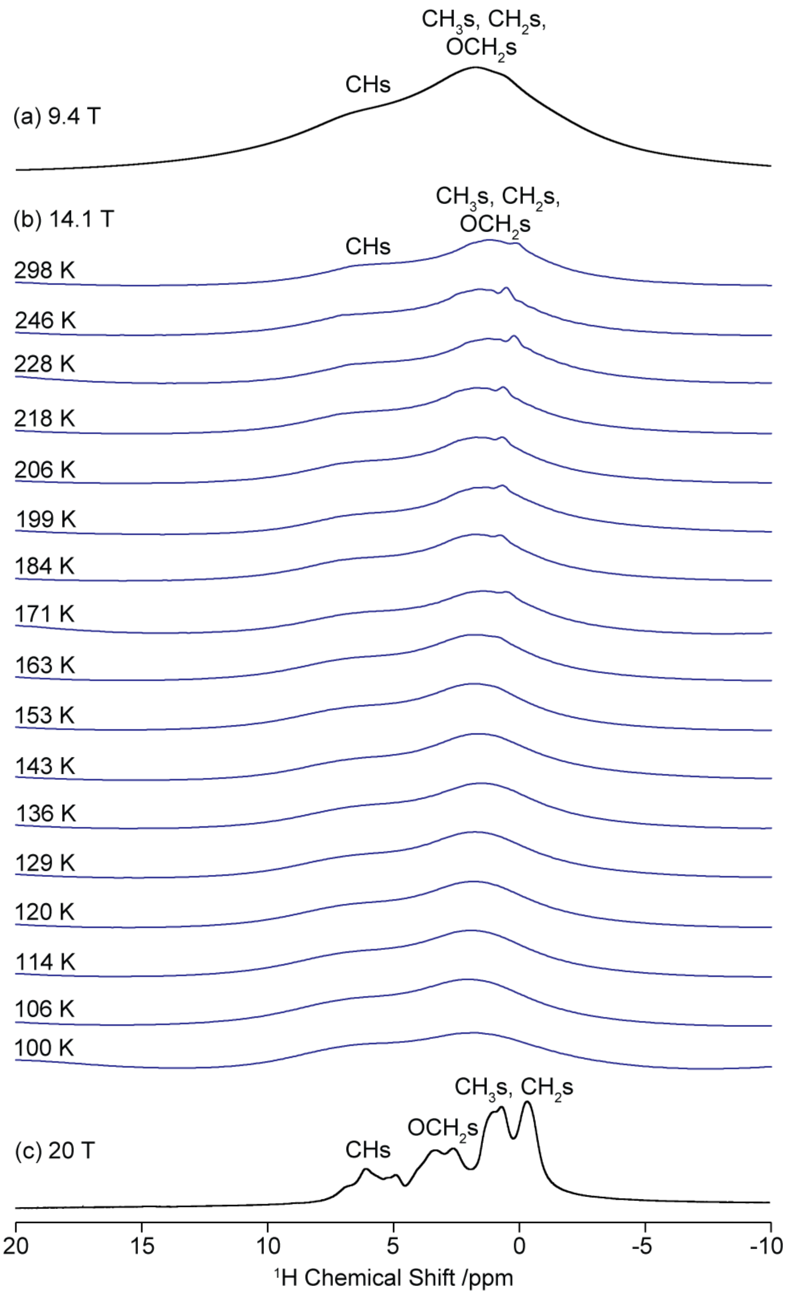

Figure S35. ^{1}H one pulse NMR spectra of ***mX@EtP6*** (a) obtained at ν_r = 12.5 kHz and 9.4 T (b) obtained under variable temperature conditions at ν_r = 12.5 kHz and 9.4 T and (c) obtained at ν_r = 60 kHz at 20 T. Spectral assignments are given in the figure.

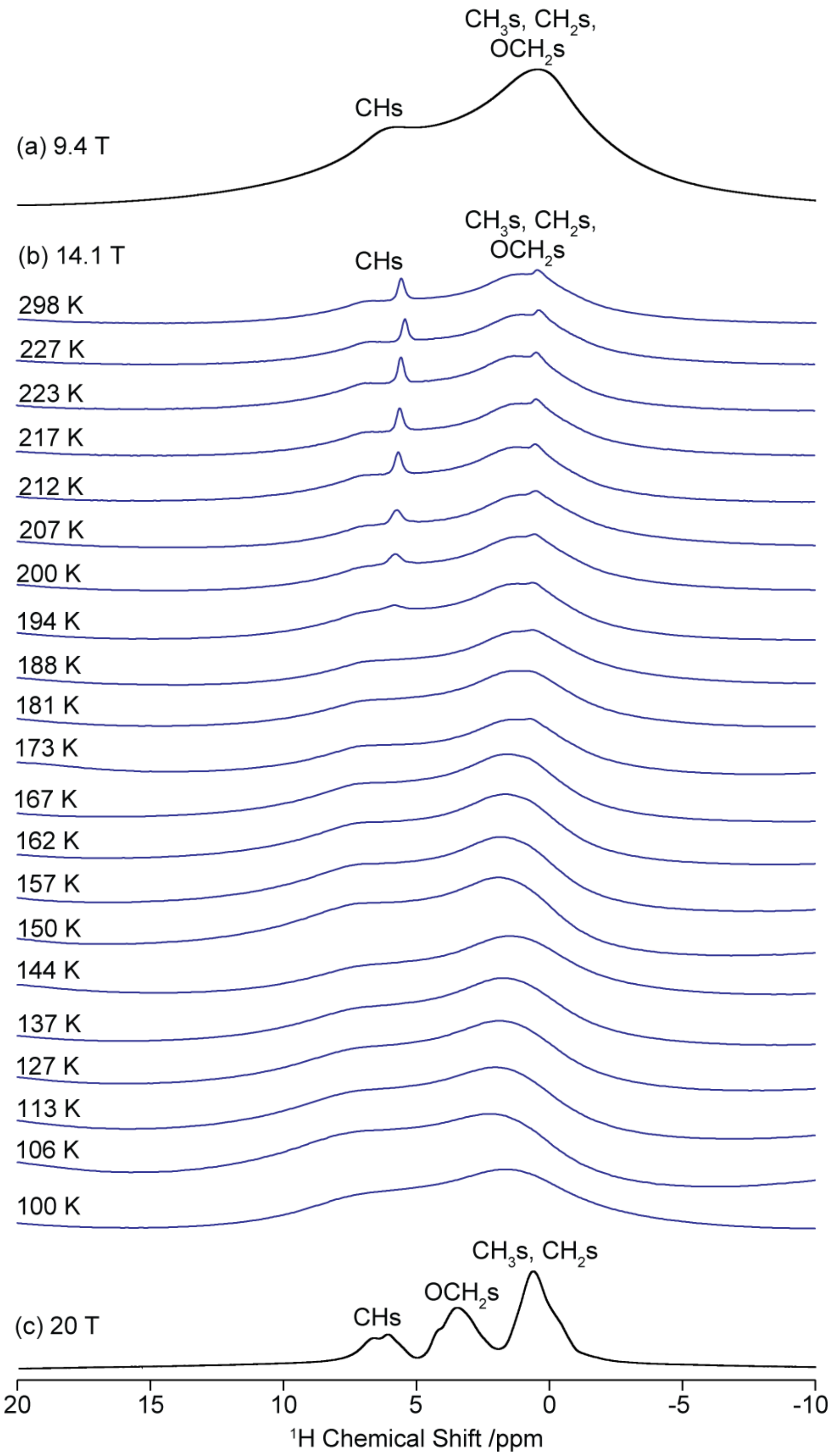

Figure S36. ^{1}H one pulse NMR spectra of ***oX*@EtP6** (a) obtained at ν_r = 12.5 kHz and 9.4 T (b) obtained under variable temperature conditions at ν_r = 12.5 kHz and 9.4 T and (c) obtained at ν_r = 60 kHz at 20 T. Spectral assignments are given in the figure.

S12. Expression of the ^{13}C chemical shift tensors

Using the Haeberlen convention,[14] these are defined as:

$$\Delta\delta_C = \delta_{33} - \frac{\delta_{11} + \delta_{22}}{2} \quad \textbf{(S5)}$$

and

$$\eta_C = \frac{\delta_{22} - \delta_{11}}{\delta_{33} - \delta_{iso}} \quad \textbf{(S6)}$$

with δ_{iso} the isotropic chemical shift (trace of the tensor):

$$\Delta\delta_C = \delta_{33} - \frac{\delta_{11} + \delta_{22}}{2} \quad \textbf{(S7)}$$

S13. References

(S1) Hu, X.-B.; Chen, Z.; Chen, L.; Zhang, L.; Hou, J.-L.; Li, Z.-T. Pillar[n]Arenes (n = 8–10) with Two Cavities: Synthesis, Structures and Complexing Properties. *Chem. Commun.* **2012**, *48*, 10999–11001.

(S2) Jie, K.; Liu, M.; Zhou, Y.; Little, M. A.; Pulido, A.; Chong, S. Y.; Stephenson, A.; Hughes, A. R.; Sakakibara, F.; Ogoshi, T. et al. Near-Ideal Xylene Selectivity in Adaptive Molecular Pillar[n]Arene Crystals. *J. Am. Chem. Soc.* **2018**, *140*, 6921–6930.

(S3) Perras, F. A.; Wang, Z.; Naik, P.; Slowing, I. I.; Pruski, M. Natural Abundance ^{17}O DNP NMR Provides Precise O−H Distances and Insights into the Brønsted Acidity of Heterogeneous Catalysts. *Angew. Chemie - Int. Ed.* **2017**, *56*, 9165–9169.

(S4) Fung, B. M.; Khitrin, A. K.; Ermolaev, K. An Improved Broadband Decoupling Sequence for Liquid Crystals and Solids. *J. Magn. Reson.* **2000**, *142*, 97–101.

(S5) Brinkmann, A.; Levitt, M. H. Symmetry Principles in the Nuclear Magnetic Resonance of Spinning Solids: Heteronuclear Recoupling by Generalized Hartmann-Hahn Sequences. *J. Chem. Phys.* **2001**, *115*, 357–384.

(S6) Carravetta, M.; Edén, M.; Zhao, X.; Brinkmann, A.; Levitt, M. H. Symmetry Principles for the Design of Radiofrequency Pulse Sequences in the Nuclear Magnetic Resonance of Rotating Solids. *Chem. Phys. Lett.* **2000**, *321*, 205–215.

(S7) Edén, M. Enhanced Symmetry-Based Dipolar Recoupling in Solid-State NMR. *Chem. Phys. Lett.* **2003**, *378*, 55–64.

(S8) Zhao, X.; Edén, M.; Levitt, M. H. Recoupling of Heteronuclear Dipolar Interactions in Solid-State NMR Using Symmetry-Based Pulse Sequences. *Chem. Phys. Lett.* **2001**, *342*, 353–361.

(S9) Lehmann, M. S.; Koetzle, T. F.; Hamilton, W. C. Precision Neutron Diffraction Structure Determination of Protein and Nucleic Acid Components. I. The Crystal and Molecular Structure of the Amino Acid L-Alanine. *J. Am. Chem. Soc.* **1972**, *94*, 2657–2660.

(S10) Krüger, T.; Vorndran, K.; Linker, T. Regioselective Arene Functionalization: Simple Substitution of Carboxylate by Alkyl Groups. *Chem. - A Eur. J.* **2009**, *15*, 12082–12091.

(S11) Abragam, A. *Principles of Nuclear Magnetism*; Oxford University Press: New York, 1963.

(S12) Steigel, A.; Spiess, H. W. *Dynamic NMR Spectroscopy*, 1st ed.; Springer-Verlag: Berlin Heidelberg, 1978.

(S13) Ibberson, R. M.; David, W. I. F.; Parsons, S.; Prager, M.; Shankland, K. The Crystal Structure of M-Xylene and p-Xylene, C_8D_{10}, at 4.5 K. *J. Mol. Struct.* **2000**, *524*, 121–128.

(S14) Haeberlen, U. *High Resolution NMR in Solids. Selective Averaging*; Academic Press: New York, 1968.
